# Comparative Performance of High-Throughput Methods for Protein p*K*_a_ Predictions

**DOI:** 10.1021/acs.jcim.3c00165

**Published:** 2023-08-08

**Authors:** Wanlei Wei, Hervé Hogues, Traian Sulea

**Affiliations:** Human Health Therapeutics Research Centre, National Research Council Canada, 6100 Royalmount Avenue, Montreal, Quebec H4P 2R2, Canada

## Abstract

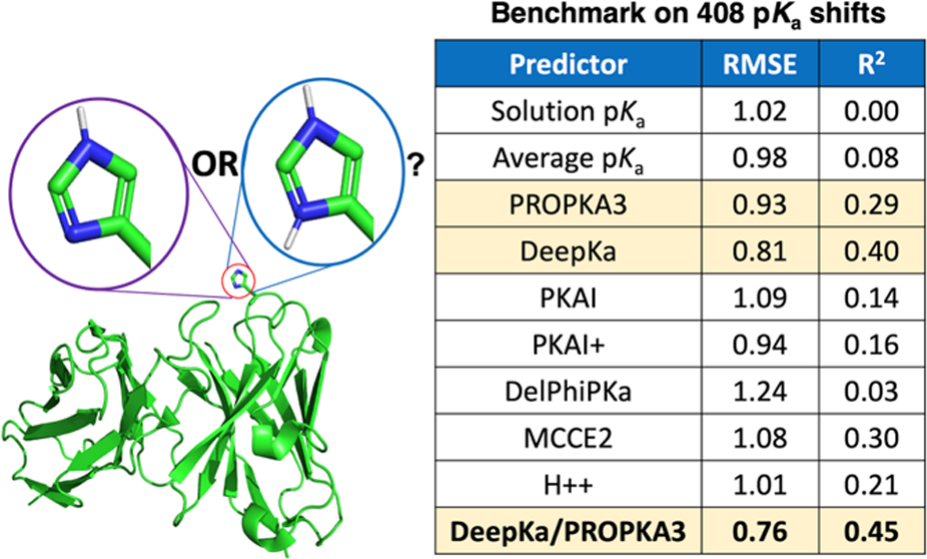

The medically relevant field of protein-based therapeutics has triggered a demand for protein engineering in different pH environments of biological relevance. *In silico* engineering workflows typically employ high-throughput screening campaigns that require evaluating large sets of protein residues and point mutations by fast yet accurate computational algorithms. While several high-throughput p*K*_a_ prediction methods exist, their accuracies are unclear due to the lack of a current comprehensive benchmarking. Here, seven fast, efficient, and accessible approaches including PROPKA3, DeepKa, PKAI, PKAI+, DelPhiPKa, MCCE2, and H++ were systematically tested on a nonredundant subset of 408 measured protein residue p*K*_a_ shifts from the p*K*_a_ database (PKAD). While no method outperformed the null hypotheses with confidence, as illustrated by statistical bootstrapping, DeepKa, PKAI+, PROPKA3, and H++ had utility. More specifically, DeepKa consistently performed well in tests across multiple and individual amino acid residue types, as reflected by lower errors, higher correlations, and improved classifications. Arithmetic averaging of the best empirical predictors into simple consensuses improved overall transferability and accuracy up to a root-mean-square error of 0.76 p*K*_a_ units and a correlation coefficient (*R*^2^) of 0.45 to experimental p*K*_a_ shifts. This analysis should provide a basis for further methodological developments and guide future applications, which require embedding of computationally inexpensive p*K*_a_ prediction methods, such as the optimization of antibodies for pH-dependent antigen binding.

## Introduction

The accurate determination of ionization constants (p*K*_a_’s) of titratable groups of biomolecules is central to the understanding of chemical processes that occur in living systems.^1−4^ Biochemical phenomena, such as protein–ligand binding,^1,4,5^ enzyme catalysis,^2,3,6^ solubility,^7,8^ membrane permeability,^9,10^ and protein folding,^11,12^ are all strongly coupled to and impacted by variations in protonation states.

Recently, the medically relevant field of protein-based therapeutics has triggered a demand for protein engineering in different pH environments of biological relevance.^13^ For example, in the case of antibody-based therapeutics, of particular interest are the selective engagement of antigens of Fcγ receptors within the acidic microenvironment of solid tumors for reduced off-tumor toxicity,^14−17^ and the selective release of antigen in the acidic endosomes for lysosomal routing and degradation.^18−21^ Structure-based engineering of pH selectivity requires reliable computational methods for the assignment of protonation states of titratable protein side chains, mainly His, Glu, and Asp, at various locations within the antibody–antigen complex. Molecular engineering of site-directed antibody–drug conjugates (ADCs) is another example that requires prediction of ionization states for other titratable protein side chains, mainly Lys and Cys, which affect their cross-linking reactivities.^22,23^ Operationally, *in silico* engineering workflows typically employ virtual high-throughput screening (vHTS) campaigns that require evaluating large sets of protein residues and point mutations that cover at least the complementarity determining regions (CDRs) of antibodies.^14,16,23−25^ Hence, speed and accuracy are both key attributes of p*K*_a_ prediction algorithms intended for screening applications.

A plethora of p*K*_a_ prediction methods that are computationally tractable have been presented over the past decades and up until very recently.^26−34^ Unfortunately, their accuracies are unclear due to the lack of any recent comprehensive benchmarking study. Although blind tests such as the “p*K*_a_ Cooperative” provided unbiased evaluation of various p*K*_a_ prediction methods, the small number of systems and titrated residues used in these contests limited the scope of their conclusions.^35,36^ Meanwhile, experimental p*K*_a_ measurements of ∼1500 residues from ∼200 proteins, mainly performed through low-throughput NMR or IR titrations,^37−39^ have been collected into the p*K*_a_ Database (PKAD),^40^ thus offering the opportunity for a more comprehensive assessment of p*K*_a_ prediction methods.

Here, we benchmarked the accuracy of the following seven popular and fast p*K*_a_ predictors, DelPhiPKa, MCCE2, H++, PROPKA3, DeepKa, PKAI, and PKAI+, on a curated PKAD subset. These methods, which are based on different paradigms ranging from physics-based, classical electrostatics to empirical and machine-learning models, are briefly described in the Methods section. For comparison, we also included published predictions on the same dataset from much slower but possibly more accurate p*K*_a_ predictions, based on constant-pH molecular dynamics (cpHMD) simulations.^33^ In addition to execution time, user friendliness of the programs was also considered, (e.g., Rosetta-pKa could not be benchmarked due to it refusing to finish computations in our tests).^31^ Other programs, which depended on the installation of many different program packages, were also not benchmarked. In addition to execution of pre-existing packages, a simple consensus approach for computing p*K*_a_’s, by averaging the best-performing methods, was also explored.

It is hoped that this benchmark study will assist other researchers in making an informed selection of p*K*_a_ and protonation state prediction methods suitable for virtual screening campaigns in protein and antibody engineering.

## Methods

### Overview of Contemporary p*K*_a_ Prediction Methods

Current structure-based p*K*_a_ prediction methods fall broadly into one of several different categories: (1) microscopic physics-based, (2) macroscopic physics-based, and (3) empirical.

Microscopic physics-based methods consider proteins and the surrounding solvents dynamically at the atomic resolution. This category includes the use of all-atom, constant-pH molecular dynamics (cpHMD) simulations and quantum mechanical (QM) methods. In cpHMD, virtual titrations of multiple protein side chains are performed simultaneously.^41−44^ This method, like free energy perturbation (FEP) and thermodynamic integration (TI), uses a λ-coupling constant to alchemically drive changes in protonation states. However, in cpHMD, λ is influenced by the environmental energetics. To compute the p*K*_a_, the relative ratio of species at various pHs could be determined and solved using the Henderson-Hasselbalch equation. Its main advantage is its consideration and simultaneous treatment of multiple titratable sites, which could influence each other. Another microscopic method is the use of QM calculations, based on computing the free energy difference between distinct protonation states.^45^ Alternatively, it could also be applied to extract QM descriptors that could be fit empirically to experimental p*K*_a_ values.^46^ While a pure QM approach is suitable for small molecules, the larger size of proteins compels the use of hybrid quantum mechanics/molecular mechanics (QM/MM) methods. The latter treats the main region of interest using the more detailed QM within the framework of faster but less accurate molecular mechanics (MM).^47^ Unfortunately, microscopic methods are computationally intractable for some desired applications, such as vHTS of small molecules and engineering of antibody mutants.

Macroscopic classical physics-based methods have been the most widely employed category of protein p*K*_a_ predictors.^26,28−30^ In these approaches, the total energy is calculated by employing MM and treating surrounding solvent water molecules implicitly as an electrostatic continuum of high dielectric constant of approximately 80. Simultaneously, a low value of between 4 and 8 is usually applied to proteins. The linearized Poisson–Boltzmann (PB) equation is often employed in implicit solvation calculations, whereby the formalism has the added benefit of considering the concentration of charged salt species. It should be emphasized that the dependence of the dielectric constant on the protein local environment and/or different media is difficult to estimate and generally not implemented in p*K*_a_ prediction methods. Popular protein p*K*_a_ predictors applying PB-based approaches include H++ (v3),^26,27^ MCCE2,^28^ DelPhiPKa,^29^ and Karlsberg2+.^30^ As these are static methods, each program has a specific flavor for treating protein conformational dynamics. For example, DelPhiPKa and H++ employ softened Gaussian-smoothed and smeared potentials, respectively, while MCCE2 performs Monte Carlo (MC) side chain rearrangements. Of note, both DelPhiPKa and MCCE2 employ the Delphi PB solver while H++ uses MEAD. Overall, PB-based methods are computationally efficient and capable of rapid p*K*_a_ predictions.

Empirical approaches approximate p*K*_a_’s based on local chemical descriptors in the form of scoring functions and effective potentials within the region of interest. PROPKA3 is the most popular and widely used of these methods, which owes its success to its ease of use and speed.^32^ More recently, machine-learning (ML) methods, which perform automated training of an expanded set of chemical descriptors, are gaining traction in the field.^33,34^ While ML could be revolutionary for p*K*_a_ prediction, it often requires a large experimental dataset for training, which is currently lacking. Unfortunately, many empirical methods developed to-date have used all available experimental pK_a_’s in the PKAD dataset for training and validation which, in this study, are likely to bias their performance due to overtraining.^32,48^ In contrast, the two promising and novel ML approaches that were selected were developed by training on thousands of simulated p*K*_a_ data for systems not included in PKAD. These are DeepKa^33^ and PKAI family^34^ of predictors, which were trained on p*K*_a_ data generated by cpHMD simulations and PB-based calculations, respectively. The former approach offered accuracies rivaling those of cpHMD simulations but at a fraction of the computational cost.^33^ Similarly, the latter family of approaches could be decomposed into PKAI and PKAI+, which differed slightly in that PKAI+, in contrast to PKAI, employed a dropout regularization term. According to the authors, this had the effect of penalizing large shifts in p*K*_a_’s, which is akin to slightly increasing the dielectric constants (ε_r_) by PB-solvers to compensate for the lack of explicit protein conformational sampling.

### Curation of Protein Dataset

Protein structures were obtained from the PDB,^49^ by cross-referencing with the protein p*K*_a_ database (PKAD).^40^ While the PKAD contained 1350 p*K*_a_’s from 157 wild-type proteins and 232 p*K*_a_’s from 45 mutant proteins, further curation was conducted to remove duplicates. The dataset also contained entries of mutant proteins, for which only wild-type structures were available. For this reason, these entries were considered separately. To ensure an unbiased comparison of different p*K*_a_ predictors, a common set of protein structures were selected based on the successful execution of all methods. This was necessary because many p*K*_a_ predictors failed to produce outputs for certain protein structures and/or side chains. For example, p*K*_a_’s of N-terminal ammonium and C-terminal carboxylate groups were excluded as they were not reported by DeepKa, DelPhiPKa, PKAI, and PKAI+. To gauge p*K*_a_ predictions in the simplest of cases, proteins containing ligands, nucleic acids, and structural metal ions were discarded. Consequently, a final list of 408 p*K*_a_’s from 56 proteins was produced (Table S1).

Two data sets of experimentally measured p*K*_a_’s and experimental protein structures were generated, which were denoted as the Large Set and Small Set. The Large Set incorporated all curated 408 p*K*_a_’s, including 141 Asp, 177 Glu, 41 His, and 49 Lys titrated residues (Table S1). Other titratable residue types (e.g., Cys, Tyr) were not included due to few representations and some p*K*_a_ predictors not reporting them (e.g., DeepKa). We noted that unshifted p*K*_a_’s were overrepresented in the Large Set. To evaluate the possible effects of this uneven distribution of p*K*_a_ shifts, a subset of 112 more evenly distributed p*K*_a_ shifts was created and termed the Small Set, which contained 34 Asp, 45 Glu, 11 His, and 22 Lys titratable residues (Table S2). This followed a similar procedure to that of Cai et al.^33^ A third dataset of experimentally measured p*K*_a_’s for single- and double-point mutants of parental proteins with experimental structures was also generated and called the Mutant Set. It consisted of curated 90 p*K*_a_’s including Glu and His titrated residues (Table S3). Structures of proteins in the Mutant Set were modeled by performing point mutations in the parental crystal structures using the program SCWRL 4.0.^50^

Water molecules were removed from all protein structures. All NMR-based protein conformations, as well as different crystal structures of identical proteins, if available (Table S4), were preserved and used for p*K*_a_ calculations. Subsequently, p*K*_a_’s obtained from various structural models were averaged and the sensitivities of each prediction method to protein conformational changes were evaluated.

### pKa Calculations

All p*K*_a_ predictors were installed and run locally. PROPKA3, DeepKa, PKAI, and PKAI+ were employed using default settings. Triplicate calculations were run for MCCE2 due to the stochastic nature of its Monte Carlo algorithm. MCCE2, H++ (v3), and DelPhiPKa were run using several different protein ε_r_ and salt concentrations (sc). More specifically, the *sc* was varied between 0 to 0.3 M in increments of 0.05 M. The ε_r_ was set to 2, 4, and 8 for MCCE and to 1, 2, 4, 8, 20, and 40 for H++. In the case of DelPhiPKa, dielectric constants of 2, 4, 8, 20, and 40 were used in combination with different force fields (FF), namely, Amber, CHARMM, GROMOS and Parse. The different choices of *sc* and ε_r_ across methods were due to characteristic differences in the parametrizations and configurations of these programs. For example, MCCE2 was parametrized using ε_r_ of 2, 4, and 8. Consequently, calculations at other ε_r_ values were nonstandard and would require further reparametrization, which was beyond the scope of this study. DelPhiPKa was also subjected to calculations of additional residue types by invoking the “Calculate More Residues: Yes” keyword option. The solvent-accessible surface area (SASA) of the titrated residue was also calculated using the software Biopython.^51^

### Performance Indices

The following error metrics were used to compare the accuracy of predictions: mean unsigned error (MUE), mean signed error (MSE), root-mean-square error (RMSE), median unsigned error (MeUE), and median signed error (MeSE) (eq 1–5).
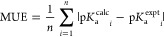
1
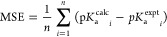
2
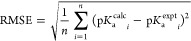
3
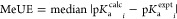
4
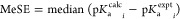
5The squared Pearson correlation coefficient, *R*^2^, was also computed between calculated p*K*_a_ shifts, Δp*K*_a*i*_^calc^, and experimental p*K*_a_ shifts, Δp*K*_a*i*_^expt^. These p*K*_a_ shifts were obtained as in eq 6, by taking as reference p*K*_a_^solution^, the experimentally measured p*K*_a_ value for the side chains of each natural amino acid type in isolation in water at 25 °C (Asp: 3.90, Glu: 4.07, His: 6.04, and Lys: 10.54).^52^
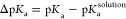
6Statistical bootstrapping was performed 10,000 times with replacement for all error metrics and the *R*^2^ using Python 3.9.

## Results and Discussion

### Global Accuracies of p*K*_a_ Predictors

**Figure 1:**
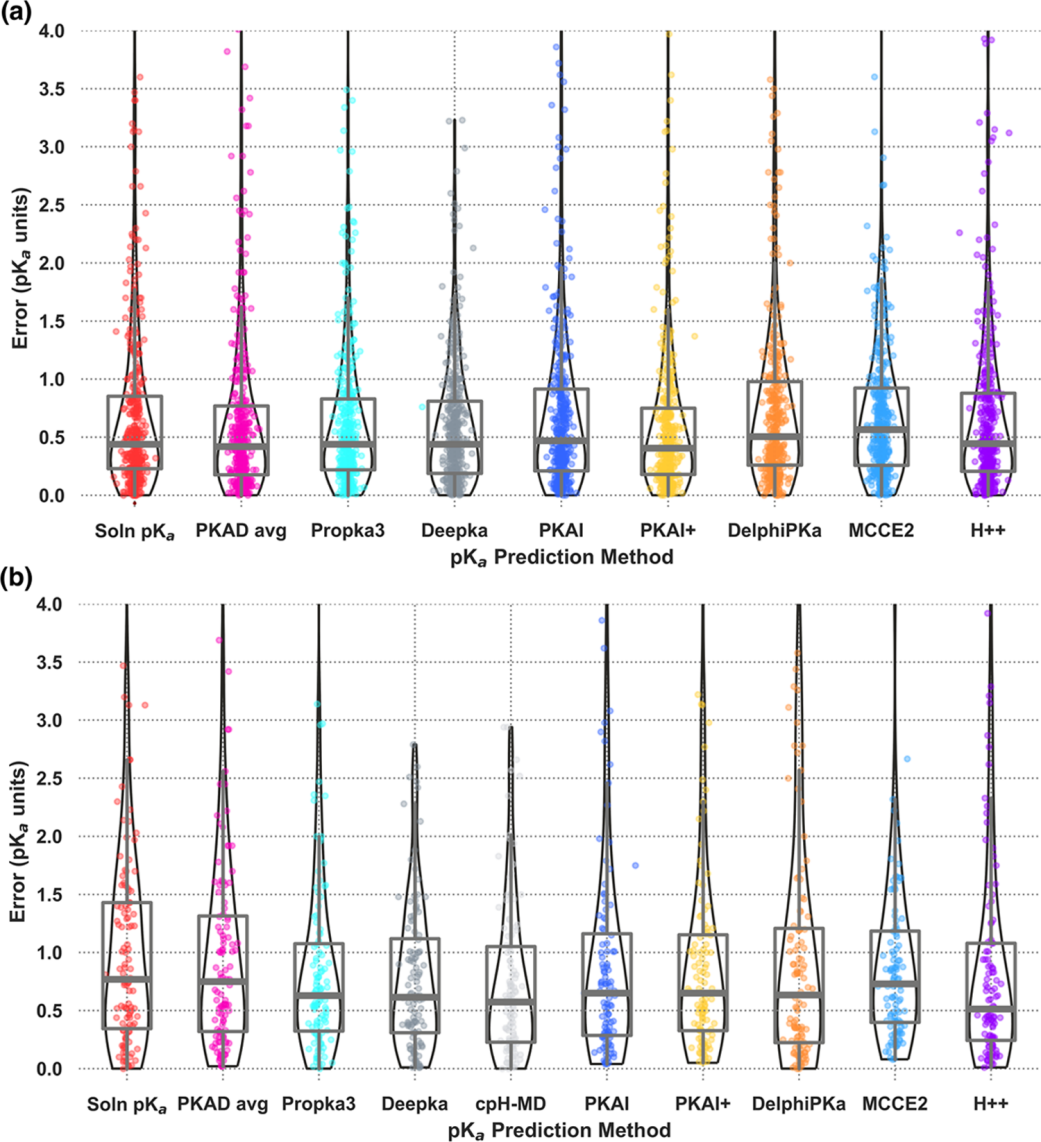
Unsigned p*K*_a_ errors reported by various predictors for all amino acid residue types. (a) Large Set (*n* = 408). (b) Small Set (*n* = 112). Solution p*K*_a_ values of isolated amino acids (null-1) and average p*K*_a_’s for each amino acid residue type in the protein context (null-2) were included as controls. Data obtained by cpHMD simulations on the Small Set were taken from Cai et al (2021).^33^

The accuracies of seven p*K*_a_ predictors were first assessed globally for all four titratable amino acid residue types included in this study: Asp, Glu, His, and Lys, in terms of unsigned error distributions for the Large Set (Figure 1A) and Small Set (Figure 1B). All experimental and predicted p*K*_a_ values for the Large Set and Small Set are listed in the Supplemental Tables S1 and S2, respectively. For the three physics-based methods: DelPhiPKa, MCCE2, and H++, data corresponding to the optimal combinations of electrostatic parameters (internal dielectric constant and salt concentration; see also Supplemental Text and Figure S1) affording the smallest mean unsigned-errors (MUE) were plotted. To gauge the robustness of each method, the experimentally measured solution p*K*_a_ values for the side chains of each natural amino acid residue type in isolation (Asp: 3.90, Glu: 4.07, His: 6.04, and Lys: 10.54)^52^ were used as a baseline and included in the benchmarking (null-1). In addition, the average experimental p*K*_a_’s for each amino acid residue type in protein environment (*i.e.*, based on the curated, nonredundant PKAD data) were also included (Asp: 3.68, Glu: 4.28, His: 6.57, and Lys: 10.51) as a comparison (null-2). For a given p*K*_a_ predictor to be useful, it should outperform both these null metrics. On the Large Set and Small Set, the Null-1 baseline gave MUEs of 0.68 and 1.02, median unsigned errors (MeUEs) of 0.44 and 0.78 and root-mean-squared errors (RMSEs) of 1.02 and 1.37, respectively. Comparably, null-2 yielded MUEs of 0.64 and 0.97, MeUEs of 0.42 and 0.73 and RMSEs of 0.98 and 1.33, on the Large Set and Small Set, respectively.

**Figure 2:**
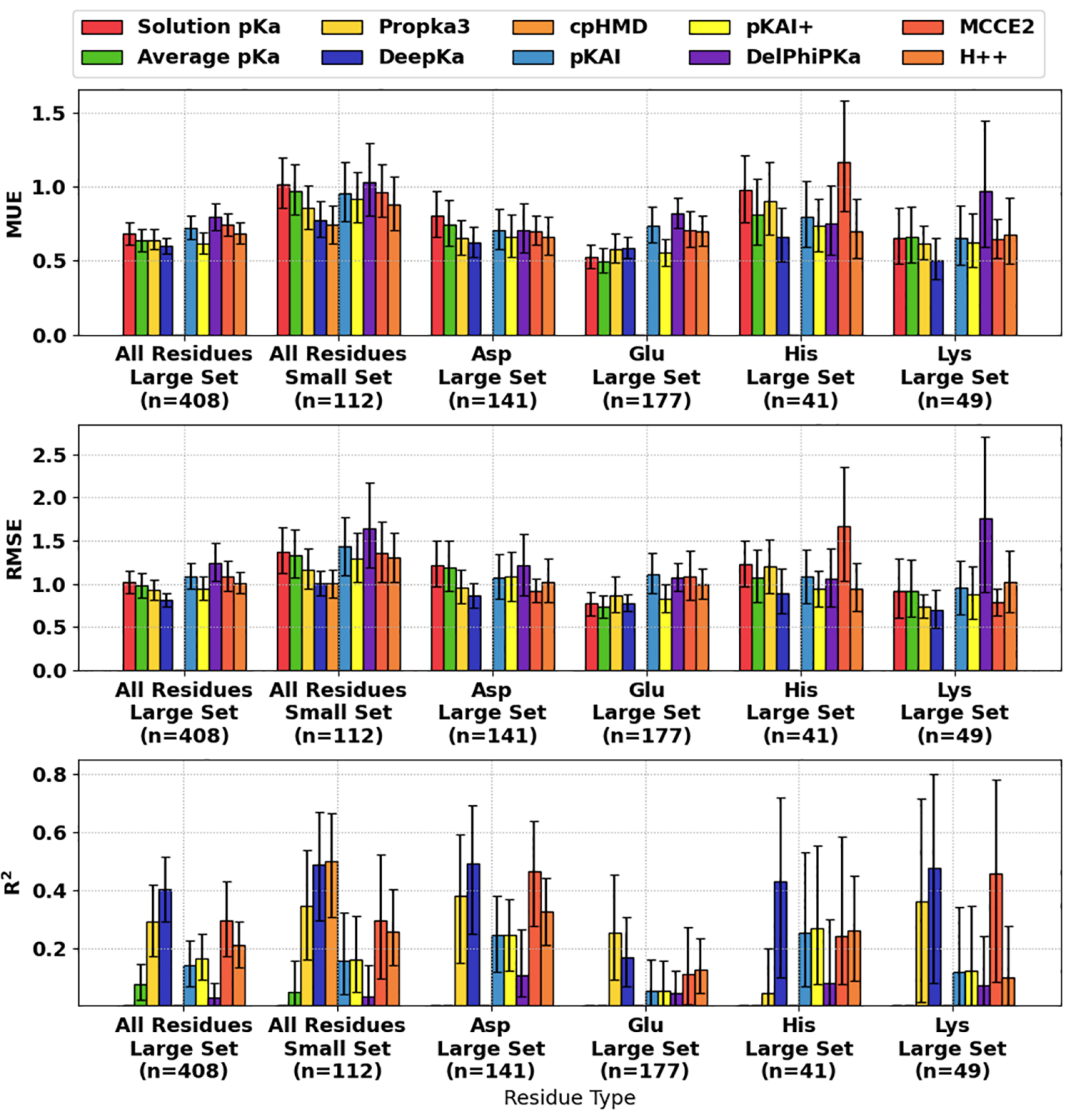
Bootstrapped performance metrics for tested p*K*_a_ predictors on various sets and subsets of p*K*_a_ data. Error bars denote 95% confidence intervals. Null models were estimated using solution p*K*_a_ and average p*K*_a_.

Five error metrics (MUE, MeUE, RMSE, MSE and MeSE; see the Methods section) were calculated on the Large and Small Sets for each prediction method. Correlation coefficients, R^2^, between experimental and predicted p*K*_a_ shifts were also obtained (*i.e.*, the offsets in absolute intrinsic p*K*_a_’s of different amino acid residue types were eliminated using null-1 as a reference). The MUE, RMSE, and *R*^2^ metrics are shown in Figure 2, whereas MeUE, MSE, and MeSE are given in Supplemental Figure S2. A summary of the main metrics, RMSE and *R*^2^, was also listed in Table 1 for all predictors, whereas all performance metrics were given in Supplemental Table S5. Computation on the Large Set indicated that DeepKa achieved a slightly higher accuracy compared to other methods, with MUE, MeUE, and RMSE of 0.60, 0.45, and 0.81, respectively. Although useful, these error metrics should be interpreted with care due to the presence of experimental errors in p*K*_a_ measurements, which have an uncertainty of approximately ± 0.1.^40^ In addition, the concentration and type of salt used during NMR measurements have been found to have a larger effect and alter experimental p*K*_a_ measurements.^53^

**Table 1:** Performance of Various p*K*_a_ Predictors and Consensus Combinations on the Overall Set and Several Subsets

		all residues large set (*n* = 408)			all residues small set (*n* = 112)			Asp large set (*n* = 141)			Glu large set (*n* = 177)			His large set (*n* = 41)			Lys large set (*n* = 49)	
predictor		RMSE	*R*^2^		RMSE	*R*^2^		RMSE	*R*^2^		RMSE	*R*^2^		RMSE	*R*^2^		RMSE	*R*^2^
solution p*K*_a_		1.02	0.00		1.37	0.00		1.22	0.00		0.77	0.00		1.23	0.00		0.91	0.00
average p*K*_a_		0.98	0.08		1.33	0.05		1.19	0.00		0.74	0.00		1.08	0.00		0.92	0.00
PROPKA3		0.93	0.29		1.16	0.34		0.96	0.38		0.86	0.25		1.20	0.05		0.74	0.36
DeepKa		0.81	0.40		1.00	0.49		0.86	0.49		0.78	0.17		0.89	0.43		0.70	0.48
PKAI		1.09	0.14		1.43	0.16		1.08	0.25		1.12	0.05		1.08	0.25		0.95	0.12
PKAI+		0.94	0.16		1.29	0.16		1.08	0.24		0.83	0.05		0.94	0.27		0.87	0.12
DelPhiPKa		1.24	0.03		1.64	0.03		1.21	0.11		1.08	0.04		1.06	0.08		1.75	0.07
MCCE2		1.08	0.30		1.35	0.30		0.92	0.47		1.09	0.11		1.66	0.24		0.79	0.46
H++		1.01	0.21		1.30	0.26		1.02	0.32		1.00	0.12		0.94	0.26		1.02	0.10
consensus-1^a^		0.82	0.40		1.10	0.45		0.91	0.54		0.75	0.18		0.82	0.44		0.77	0.42
consensus-2^b^		0.76	0.45		0.98	0.49		0.83	0.51		0.67	0.29		0.90	0.44		0.67	0.45
consensus-3^c^		0.83	0.34		1.11	0.37		0.93	0.42		0.69	0.23		1.02	0.18		0.74	0.36
consensus-4^d^		0.77	0.46		1.03	0.49		0.87	0.53		0.66	0.27		0.88	0.42		0.71	0.44

a DeepKa/PKAI+.

b DeepKa/PROPKA3.

c PROPKA3/PKAI+.

d DeepKa/PKAI+/PROPKA3.

**Figure 3:**
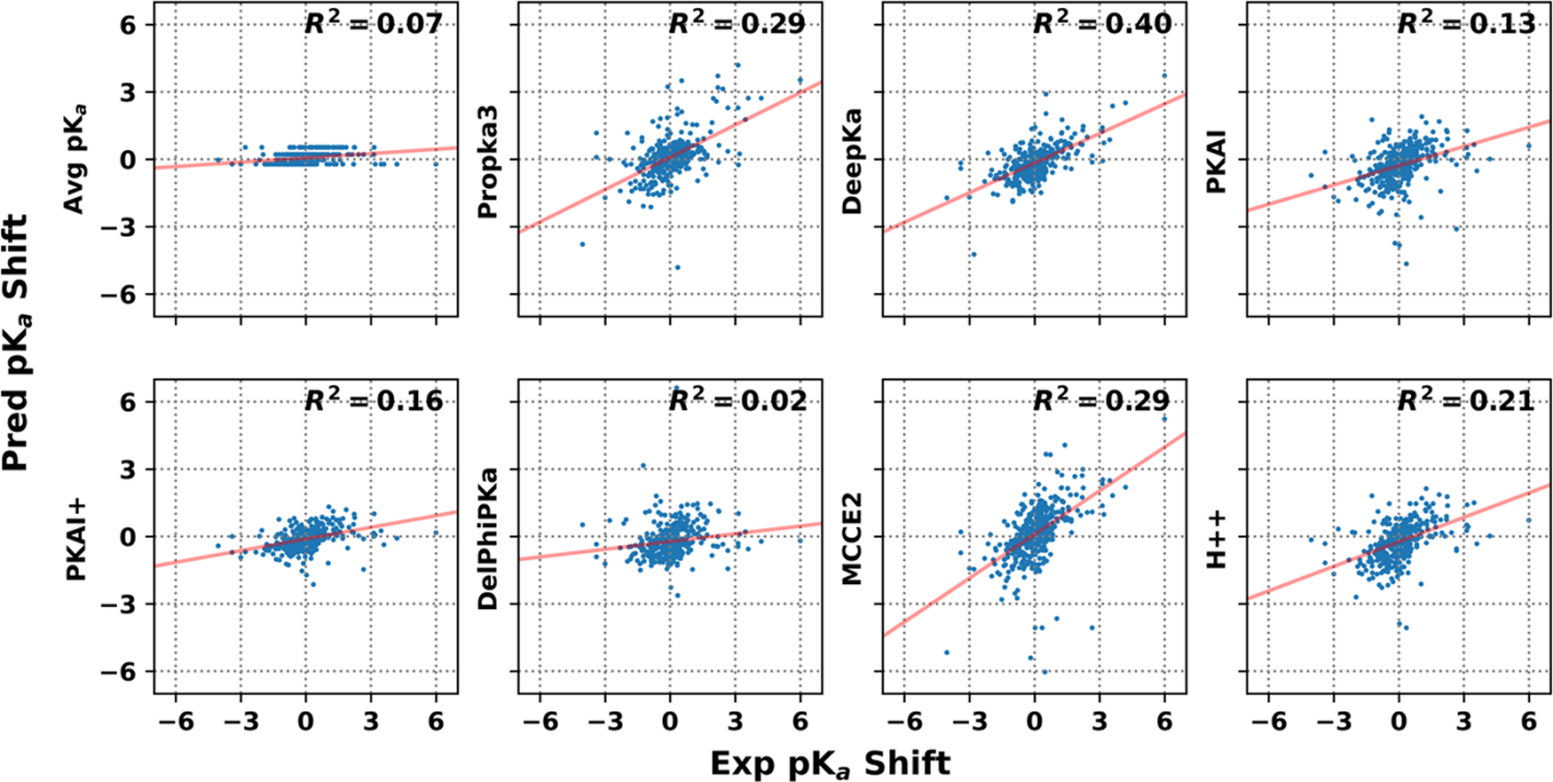
Correlation plots between predicted and experimental p*K*_a_ shifts. Least-square fit correlation line was indicated in red, and the associated squared Pearson correlation coefficient was labeled for each method.

Compounded to this issue is the relatively small size of the dataset (*n* = 408). To address the latter, statistical bootstrapping was performed to approximate the median value and assign a 95% confidence interval to all error measurements. Results on the Large Set showed that no method outperformed the null-2 model convincingly. In fact, the error bars of the null-2 model overlapped with the best-performing method, DeepKa, which also occurred for the Small Set. Nevertheless, DeepKa achieved the highest *R*^2^ of 0.40 for p*K*_a_ shifts, which was more convincingly superior to null models and other predictors. Correlations between predicted and experimental p*K*_a_ shifts were given for all methods as scatter plots in Figure 3. A third source of possible errors was the quality and accuracy of the structural models which were used. As many methods rely upon the calculation of electrostatic and van der Waals energies, differences in atomic positions could significantly affect the predicted p*K*_a_’s (see the Sensitivity of p*K*_a_ Predictors to Structural Variations section).

Despite the above challenges, comparisons of median error metrics were conducted. It was found that PKAI+ and PROPKA3 attained similar levels of accuracy when evaluated on the Large Set. PROPKA3 showed similar performance to PKAI+ when measured by RMSE (0.93 *vs* 0.94), MeUE (0.44 *vs* 0.40), and MUE (0.64 *vs* 0.63). Across all error metrics, the performances of these two methods were comparable to that of null-2. Interestingly, PROPKA3 slightly outperformed null-2 when measured by RMSE with a difference of 0.05. As RMSE is more sensitive to outliers, where a few predictions with large error gaps could greatly influence the error metric, it suggested that PROPKA3 could produce more consistent accuracy than null-2. The relatively higher *R*^2^ of 0.29 for PROPKA3 compared to 0.16 for PKAI+ indicated the former’s good underlying correlation and resolving power. Interestingly, the top three performers were all empirical methods.

The other tested p*K*_a_ predictors all attained lower accuracies compared to the null-2 model on the Large Set. In fact, H++, PKAI, MCCE2, and DelPhiPKa produced RMSEs of 1.01, 1.09, 1.08, and 1.24, respectively, compared to 0.98 for null-2. In addition, their respective MUEs of 0.68, 0.72, 0.74, and 0.79 were greater than that of null-2 (0.64). Similarly, MeUEs for these methods were also greater compared to null-2, especially for MCCE2. While these results were discouraging, it should be noted that many of these methods carried some utility as evaluated by correlation *R*^2^ between p*K*_a_ shifts. For example, MCCE2 yielded an *R*^2^ of 0.30, which suggested that the underlying physics-based approach has the potential to predict p*K*_a_’s. A recalibration of the method could help rescue its performance and reduce errors.

Interestingly, many of the tested p*K*_a_ predictors did not produce median signed errors (MeSE) nor mean signed errors (MSE) close to zero. In fact, DeepKa, PKAI, DelPhiPKa, and H++ exceeded ±0.2 p*K*_a_ units in one of these two metrics. In contrast, the null models registered very small MeSEs and MSEs below ±0.06 p*K*_a_ units in both metrics.

Evaluation of these error metrics on the Small Set, having more evenly distributed p*K*a shifts, produced similar results. Due to the greater representation of larger p*K*_a_ shifts, all methods suffered accuracy losses. However, the two null models suffered greater deterioration in the quality of their predictions as they employed fixed values for each amino acid. A few other notable differences were revealed. First, the accuracy of PROPKA3 did not deteriorate as greatly as that of PKAI+ (RMSE of 1.16 *vs* 1.29; MUE of 0.85 *vs* 0.92, respectively). Second, H++ improved in its ranking and outperformed null-2 in all metrics. In fact, H++ was the sole method capable of attaining an MeUE of less than 0.5. Apart from this metric however, DeepKa was still the most well-rounded, best-performing method with regards to MUE and RMSE. It registered more convincing improvements in errors relative to the null models and reached an *R*^2^ value of 0.49 when the p*K*_a_ shifts were compared. The performance of DeepKa was almost indistinguishable from that of the cpHMD method on the Small Set, which was expected given that DeepKa was calibrated on cpHMD simulations (but outside of the PKAD dataset). Crucially, DeepKa required much less computational resources and expertise compared to cpHMD.

It has been previously noted that the accuracy of p*K*_a_ predictions may be influenced, to some extent, by the degree of surface exposure or burial of the titrated residue under investigation.^36^ The data obtained here, on the Large Set, appears to support that notion (Figure S3). First, most of the larger absolute p*K*_a_ shifts (>2.5 log units) from the solution p*K*_a_ of isolated amino acids were found to be associated with ionizable groups which were more buried in the protein environment. Second, it was a common feature for both classes of empirical and physics-based methods to report larger errors for residues with little or no surface exposure.

### Accuracies of p*K*_a_ Predictors on Specific Amino Acid Types

We also calculated the accuracies of each predictor for specific types of amino acids using data from the Large Set (Table 1, Figure 2, and Figure S2). This was not possible on the Small Set due to the limited number of entries for each amino acid residue type. The importance of histidyl residues, which titrate within the pH ranges of biological systems, motivated us to scrutinize them in detail. In total, the p*K*_a_’s of 41 histidyl residues were predicted by various methods on the Large Set. While the solution p*K*_a_ of His was elucidated to be approximately 6.04, its average observed p*K*_a_ from the nonredundant, curated PKAD dataset was approximately 6.6. Due to this large difference, the accuracies offered by the two null models differed significantly for His. The null-1 baseline afforded RMSE, MUE, and MeUE of 1.23, 0.98, and 0.78, respectively; while the null-2 baseline gave 1.08, 0.81, and 0.68, respectively. Several methods, including DeepKa, PKAI+, H++, and DelPhiPKa, marginally outperformed both nulls across all error metrics. Two separate groups of methods emerged which had their own strengths and weaknesses. The two empirical methods DeepKa and PKAI+ achieved lower RMSEs than the physics-based methods H++ and DelPhiPKa, while the latter group achieved lower MeUE than the first group. Numerically, DeepKa was the slightly better empirical predictor (RMSE and MeUE of 0.89 and 0.61, respectively, and an *R*^2^ of 0.43) while H++ was the slightly better physics-based predictor (RMSE and MeUE of 0.94 and 0.47, respectively, and an *R*^2^ of 0.26). The other predictors, PROPKA3, PKAI, and MCCE2 did not offer any clear advantages over null-2. The number of nonredundant histidyl p*K*_a_ data, particularly for those with large deviations in the solution values, should be expanded in the future for a more comprehensive benchmarking.

Lysine p*K*_a_ predictions are also of practical interest in certain biologics engineering applications, with antibody–drug conjugates (ADCs) as an example.^23^ A total of 49 lysyl p*K*_a_’s comprised the current dataset. The null-2 baseline afforded RMSE, MUE, and MeUE of 0.92, 0.66 and 0.51, respectively. Several p*K*_a_ predictors were able to outperform the null-2 model. However, one method, DeepKa, surpassed others by producing RMSE, MUE, and MeUE of 0.70, 0.51, and 0.39, respectively. In addition, DeepKa attained an *R*^2^ of 0.48, which was the highest, and greater than 0.56 obtained by PROPKA3. Interestingly, MCCE2 also yielded an *R*^2^ of 0.46. The presence of a significant MSE, coupled with a robust *R*^2^, might indicate that a recalibration of MCCE2 could improve its lysyl p*K*_a_ predictions.

The two acidic amino acid residue types, aspartic and glutamic, were also analyzed separately and provided a larger number of p*K*_a_ data points. A total of 141 aspartyl and 177 glutamyl residues were found in the Large Set. The null-2 baseline afforded RMSE, MUE, and MeUE of 1.19, 0.75, and 0.47, respectively, for Asp; and 0.74, 0.50, and 0.32, respectively, for Glu. An interesting observation was that, while all methods outperformed the null models in terms of RMSE for the prediction of Asp p*K*_a_’s, no predictor could outperform null-2 by any error metric for Glu p*K*_a_’s. In the aspartic acid subset, PROPKA3 and DeepKa stood out with the highest overall accuracies. The best performance was from PROPKA3, which achieved RMSE and MeUE of 0.96 and 0.39, respectively. DeepKa also showed promise for Asp p*K*_a_ prediction, with an RMSE of 0.86 and a relatively high *R*^2^ of 0.49. For Glu p*K*_a_ prediction, the best two methods were DeepKa and PKAI+. However, as mentioned earlier, their RMSE, MUE, and MeUE of 0.78, 0.59, and 0.44 for DeepKa and 0.83, 0.55, and 0.37 for PKAI+, respectively, did worse than the null models. Furthermore, the highest *R*^2^ value of only 0.25, achieved by PROPKA3, was significantly poorer than the *R*^2^ achieved for Asp p*K*_a_ prediction.

### Classification Abilities of p*K*_a_ Predictors

**Figure 4:**
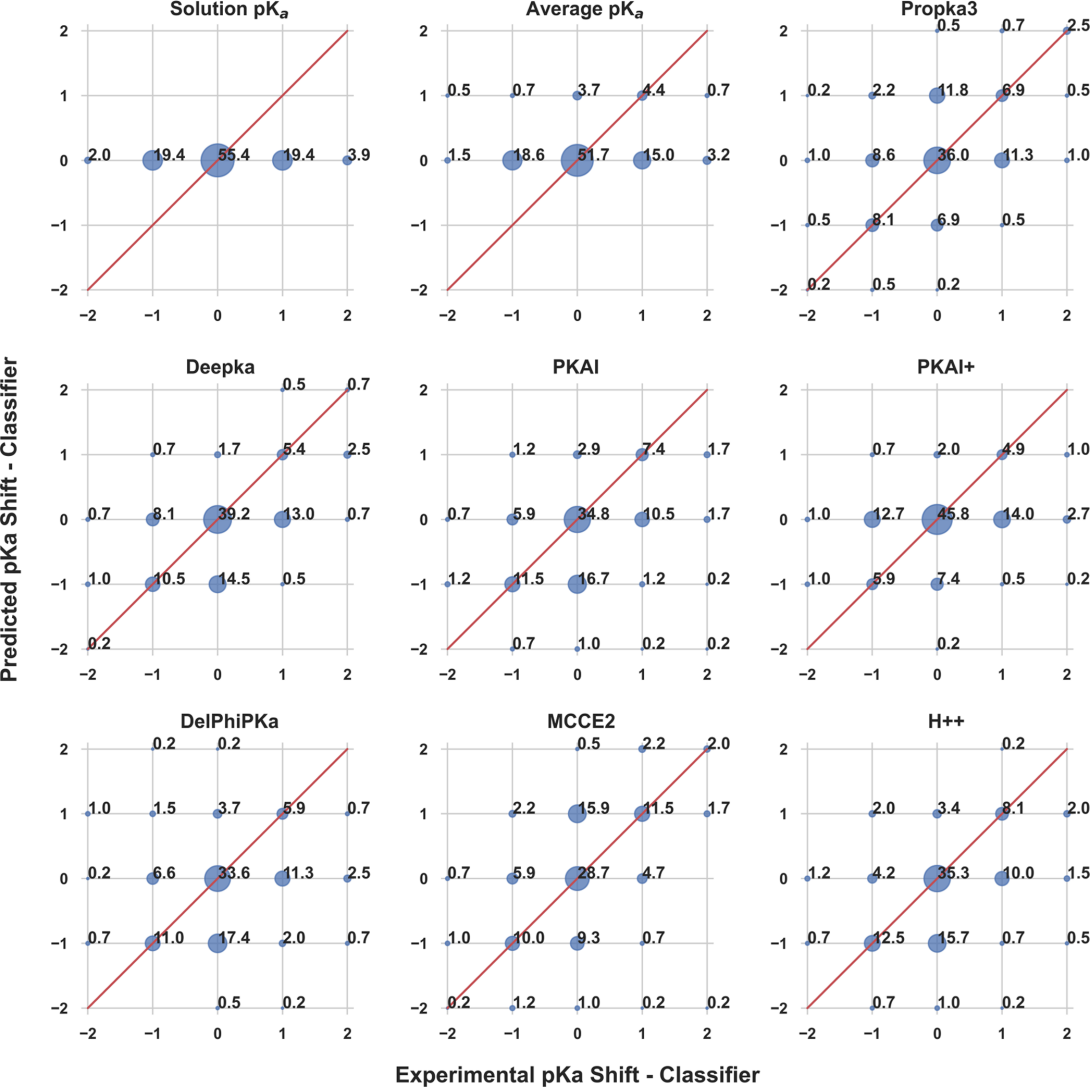
Classification performance of various p*K*_a_ predictors. Quantized estimates for the direction and magnitude of p*K*_a_ shifts are shown as percentages (%) for the Large Set. The overall accuracy of each method is equaled to the sum along the diagonal line. Quantized accuracies are 55.6% for solution p*K*_a_, 51.6% for average p*K*_a_, 53.5% for PROPKA3, 55.7% for DeepKa, 54.1% for PKAI, 56.7% for PKAI+, 50.6% for DelPhiPKa, 51.8% for MCCE2, and 55.6% for H++.

The usefulness of these p*K*_a_ predictors was also evaluated with a semiquantitative approach. A quantized classifier was defined to assess the ability of different methods to predict the general direction and magnitude of p*K*_a_ shifts without an overly stringent criterion on a precise numerical value. As such, pK_a_ shifts were categorized into five distinct bins or regions: [-∞,–2), [−2, −0.5), [−0.5, 0.5], (0.5, 2], and (2, ∞]. These corresponded to p*K*_a_’s which were significantly downshifted, moderately downshifted, negligibly shifted, moderately upshifted, and significantly upshifted, respectively. This classification was applied to both the experimental and predicted p*K*_a_’s. To obtain a perfect classification score, all predicted p*K*_a_ shift data points needed to be placed in the corresponding bins determined experimentally. When plotted, these should fall on the indicated diagonal lines of slope 1 (Figure 4). The fraction of p*K*_a_ shifts predicted in the correct bins (and lying on the diagonal line) could thus be taken as a measure of classification accuracy. A limitation of this assessment in terms of classification ability is the choice of p*K*_a_ bin boundaries. As with any discrete method, two very similar values could fall on either side of the boundary and be assigned into different bins. To preclude this possibility, various bin sizes were attempted by adjusting the boundaries in small increments as follows: [-∞,–2-δ), [−2-δ, −0.5-δ), [−0.5-δ, 0.5 + δ), (0.5 + δ,2 + δ], and (2 + δ,∞]. It should be noted that employing δ < −0.1 penalized the null models to a greater degree than the other p*K*_a_ predictors due to the former using fixed p*K*_a_ values.

Solution p*K*_a_ values (i.e., p*K*_a_ shifts of zero) were applied as the crudest predictor for our null-1 baseline, which achieved a classification accuracy of 55.6%. Unexpectedly, no method noticeably outperformed the null model, with PROPKA3 (53.5%), DeepKa (55.7%), PKAI (54.1%), PKAI+ (56.7%), and H++ (55.6%) achieving accuracy within ±2% of null-1. This was the case, given that δ > 0.1, for the bin sizes employed. In addition, both MCCE2 (51.8%) and DelPhiPKa (50.6%) attained noticeably lower classification accuracies than the null-1 model. Overall, this was not surprising as most methods achieved similar error metrics (i.e., MUE, RMSE, and MeUE) as that of the solution (null-1) and average p*K*_a_’s (null-2).

In contrast to the overall classification accuracy, predictions of individual amino acid types followed a similar trend to the previously presented error metrics (Figures S4–S7). In brief, the classification accuracies of several methods surpassed that of the null hypothesis when applied to Asp, His, and Lys. For example, H++ attained a classification accuracy of 61.7% on Asp, which was noticeably higher than the 51.8% attained by the null hypotheses. In fact, all other methods comfortably outperformed the null models by at least 3%. These p*K*_a_ predictors were also applied to histidyl p*K*_a_ classification, which showed that DeepKa was the most useful. DeepKa attained the highest classification ability, with 60.9% accuracy compared to 39 and 41.5%, yielded by null-1 and null-2, respectively. Lysyl p*K*_a_ classification was also successfully performed by various methods. Interestingly, H++ and PKAI were able to attain accuracies of 61.2 and 61.1%, respectively, which were significantly higher than either null model. Lastly, glutamyl p*K*_a_ prediction was the most challenging, with both null models attaining a classification accuracy of 64.9%, which was superior to that of the best-performing method, PKAI+, which had an accuracy of 59.7%.

In general, apart from the classification of Glu p*K*_a_’s, many methods were useful to predict the direction and magnitude of p*K*_a_ shifts and offered a noticeable advantage over the null models. In fact, for Asp, His, and Lys p*K*_a_’s, DeepKa, PKAI, and H++ were found to consistently offer the most robust classification accuracies. They attained classification accuracies of 57.77, 57.03, and 56.43%, respectively, over the 46.73% obtained by the null-2 model. Despite this success, there is still significant room for improvement.

An interesting strategy in the analysis of classification ability data of various predictors was to focus on the most shifted p*K*_a_’s, which in many cases are known to be associated with functional sites (e.g., reactive catalytic residues of enzyme active sites). With this focus in mind, by examining the classification plots shown in Figure 4, it was immediately apparent that some predictors (PROPKA3, DeepKa, MCCE2) have utililty as compared to the null models by placing a significant number of the larger pK_a_ shifts (those beyond ±2 log units) in the correct bins. Conversely, however, the same methods also predicted large p*K*_a_ shifts for some experimentally unperturbed residues (which, by definition, are placed in the correct bin by the null models) – an undesirable outcome from an engineering perspective.

### Consensus Approach to p*K*_a_ Prediction

Consensus approaches are often used in other areas of *in silico* molecular property predictions (e.g., docking, binding affinity scoring, and water placement). DeepKa, PROPKA3, and PKAI+ obtained consistently higher accuracies than other methods and achieved *R*^2^ values of 0.40, 0.29, and 0.16 when benchmarked to experiment, respectively. However, the cross-correlations between these empirical methods were not high (Figure S8), which was likely the result of their different underlying training data. While PROPKA3 was parametrized on experimental p*K*_a_’s, DeepKa was trained on cpHMD simulations. Lastly, PKAI methods learned to recognize p*K*_a_’s derived from PB calculations. The relatively low pairwise correlation between the three empirical methods and their moderate correlations to experiment justified their amalgamation into a consensus approach. In addition, they appeared to identify and register different signals when provided with the same protein structure. It was hoped that the use of a consensus approach could enable Glu p*K*_a_ protonation state prediction, which was previously found to be lacking. Furthermore, as the errors of DeepKa were left-shifted while those of PROPKA3 were right-shifted relative to the null model, it was hypothesized that the combination of these two approaches could yield improved results. Consequently, these three methods were deemed good candidates to build a consensus approach.

In total, four consensuses were developed, based on simple arithmetic averages of predicted p*K*_a_ values. The first consensus approach involved the use of DeepKa in conjunction with PKAI+, which was termed consensus-1. Consensus-2 was designated as the simple average of DeepKa and PROPKA3. Similarly, consensus-3 was the combination of PKAI+ and PROPKA3. Finally, consensus-4 was the arithmetic average of all three methods: DeepKa, PKAI+, and PROPKA3. It should be mentioned that no optimization of coefficients nor training was performed. The performances of these four consensus methods were evaluated by using previously defined metrics: MeUE, MUE, RMSE, and *R*^2^ on the Large Set and its amino acid residue type subsets.

**Figure 5:**
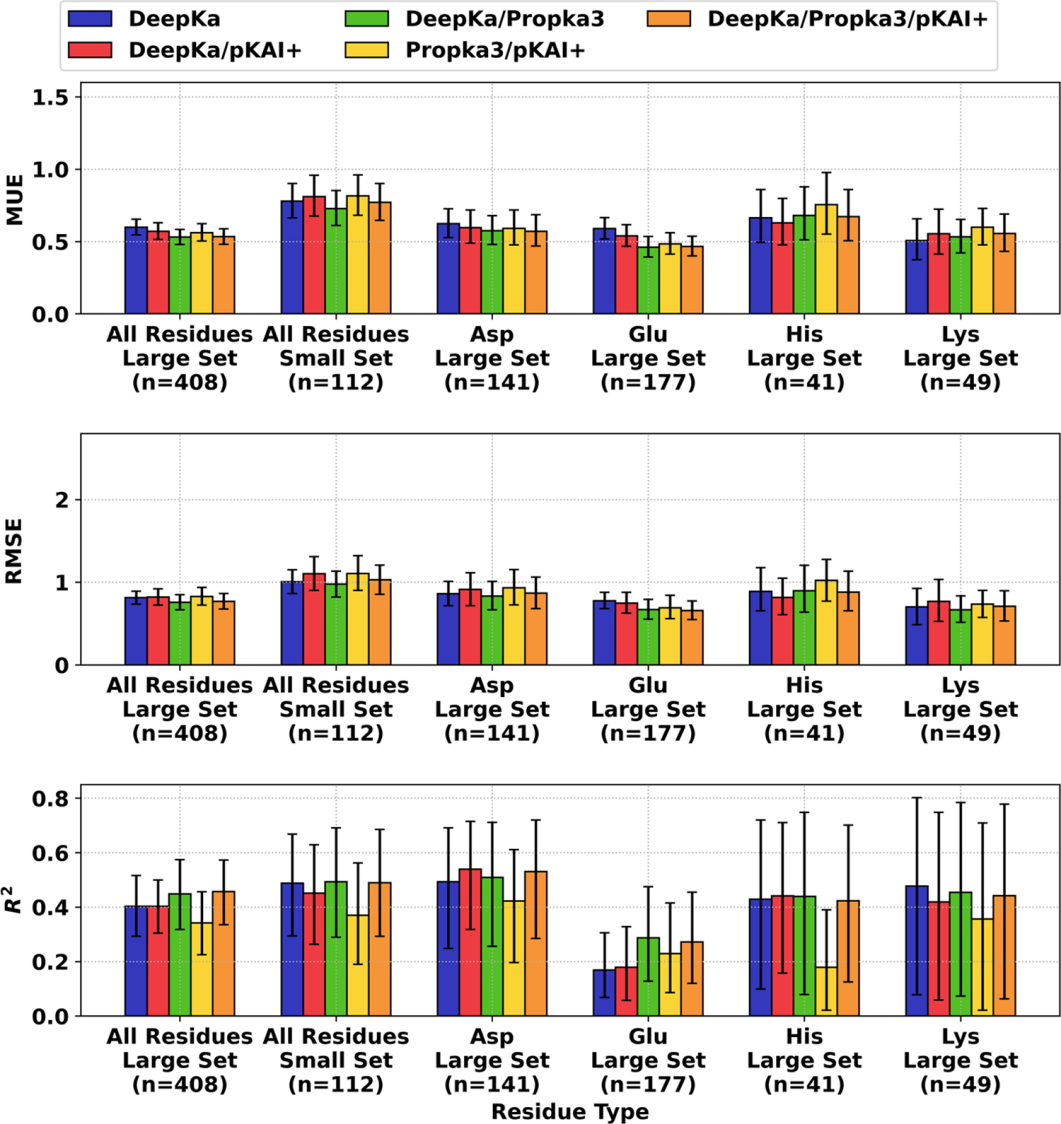
Performance of the consensus approach combining empirical p*K*_a_ predictors. Data for DeepKa predictions were included as reference. Error bars denote 95% confidence intervals.

Interestingly, the performance of consensus-2, combining DeepKa and PROPKA3, yielded the highest overall performance, which marginally outperformed consensus-4 (Table 1, Figures 5, and S9). An RMSE, MUE, MeUE, and *R*^2^ of 0.76, 0.53, 0.38, and 0.45, respectively, for consensus-2, and 0.77, 0.54, 0.39, and 0.46 for consensus-4, indicated that both methods surpassed the accuracies of DeepKa, PROPKA3 or PKAI+ individually. When looking at the p*K*_a_ for each of the His, Lys, Asp, and Glu residue types separately, both consensuses 2 and 4 were also found to possess the highest improved accuracies relative to individual methods. Notably, these consensus methods showed utility for predicting Glu p*K*_a_’s, a feat which was not accomplished by any of the individual predictors when compared to the null baseline. This was also reflected in a noticeable improvement in the classification accuracy with consensus-1 to −4 yielding 58, 60.8, 59.2, and 60.7%, respectively (Figure S10).

Previously, a conceptually similar approach had been employed for estimating p*K*_a_’s, using a Bayesian model.^54^ During that endeavor, 11 methods constituted the consensus approach, which was found to also outperform individual methods. However, the coefficients and weights were extensively trained on the available experimental data. Our approach differs in that only up to three methods were combined using a simple arithmetic average, with no prior training on the validation set. The latter was deemed important due to the risk of overfitting due to insufficient p*K*_a_ data.

### Sensitivity of p*K*_a_ Predictors to Structural Variations

Proteins are dynamic biomolecules, capable of structural rearrangements in solution. While some movements are small and local, others could be larger and fluctuate at the domain level. As experimentally measured p*K*_a_’s are a Boltzmann weighted average of these various states, empirical and macroscopic p*K*_a_ prediction methods rely on a static structure with limited sampling of dynamic states. Consequently, they are dependent on the input structure.

It is also known that protein conformations influence the p*K*_a_ of protein-titratable residues. For example, the Lys^258^ residue of aminotransferase undergoes a two-unit increase of its p*K*_a_ upon ligand binding, which causes significant conformational changes. This increase in p*K*_a_ enables Lys^258^ to act as a nucleophile.^55^ The availability of NMR structures with multiple protein conformations enabled each model to be subjected to p*K*_a_ calculations. In addition, multiple X-ray crystal structures of identical proteins were treated in a similar fashion. These allowed an evaluation of the effect of structural variability on the p*K*_a_ sensitivity for each evaluated predictor.

**Figure 6:**
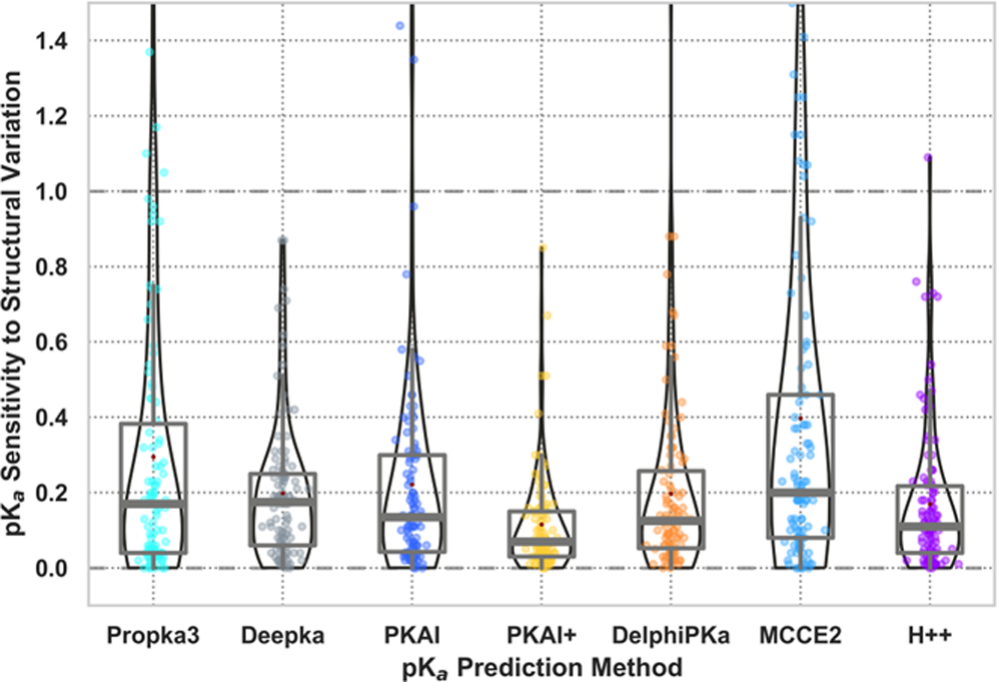
Sensitivity to structural deviations for various p*K*_a_ predictors. Data was obtained on 120 structures for which more than one 3D model was available from NMR or X-ray crystallography experiments.

Computations on 120 structures showed that different programs had varying degrees of p*K*_a_ sensitivities to structural perturbations (Figure 6). In order, from the least to the most sensitive, PKAI+, H++, DelPhiPKa, PKAI, DeepKa, PROPKA3, and MCCE2 showed mean unsigned fluctuations of predicted p*K*_a_ of 0.12, 0.17, 0.19, 0.2, 0.22, 0.29, and 0.38 units, respectively. Ranking by median unsigned fluctuations gave 0.07, 0.11, 0.12, 0.14, 0.18, 0.17, and 0.20, respectively. While it was previously conjectured that empirical methods should be less sensitive to structural differences than physics-based methods, this was not found to be true. All tested methods were structure-based and relied on atomic coordinates, which could partly explain this sensitivity. Other factors seemed to influence prediction sensitivity, including the choice of *sc* and ε_r_ constants. For example, the choice of ε_r_ = 4 and sc = 0 M over ε_r_ = 8 and sc = 0.15 M for MCCE2 decreased the mean unsigned fluctuation from 0.61 to 0.40 and median unsigned fluctuation from 0.32 to 0.20. In addition, the use of smoothed energy potentials in DelPhiPKa and H++, and the regularization constant in PKAI+, likely helped decrease structural sensitivity. Surprisingly, MCCE2 was expected to be one of the least sensitive to structural variation due to its MC sampling algorithm, while the opposite was found here. It was reasoned that protein side chain sampling would enable different protein structures to always arrive at the local minimum, irrespective of its starting point. However, as protein conformational states from models could differ more drastically, including large-scale backbone and secondary structure variations, MC sampling was not designed to this end, which might help explain these convergence issues. An analysis of p*K*_a_ predictions that were found to be general outliers by all evaluated methods also underlined structural variations as possible causes of mispredictions, pointing to a need for more careful preparation and representation of the structural data used as input for p*K*_a_ calculations (see Supplemental Material and Table S7). Relationships between structural variability (RMSD) and calculated p*K*_a_ shift errors with each method were included in Figure S11 as this information might help developers of p*K*_a_ prediction methods looking into fundamental problems related to underlying structural models.

### Preliminary Assessment of p*K*_a_ Prediction on Modeled Protein Structures

**Figure 7:**
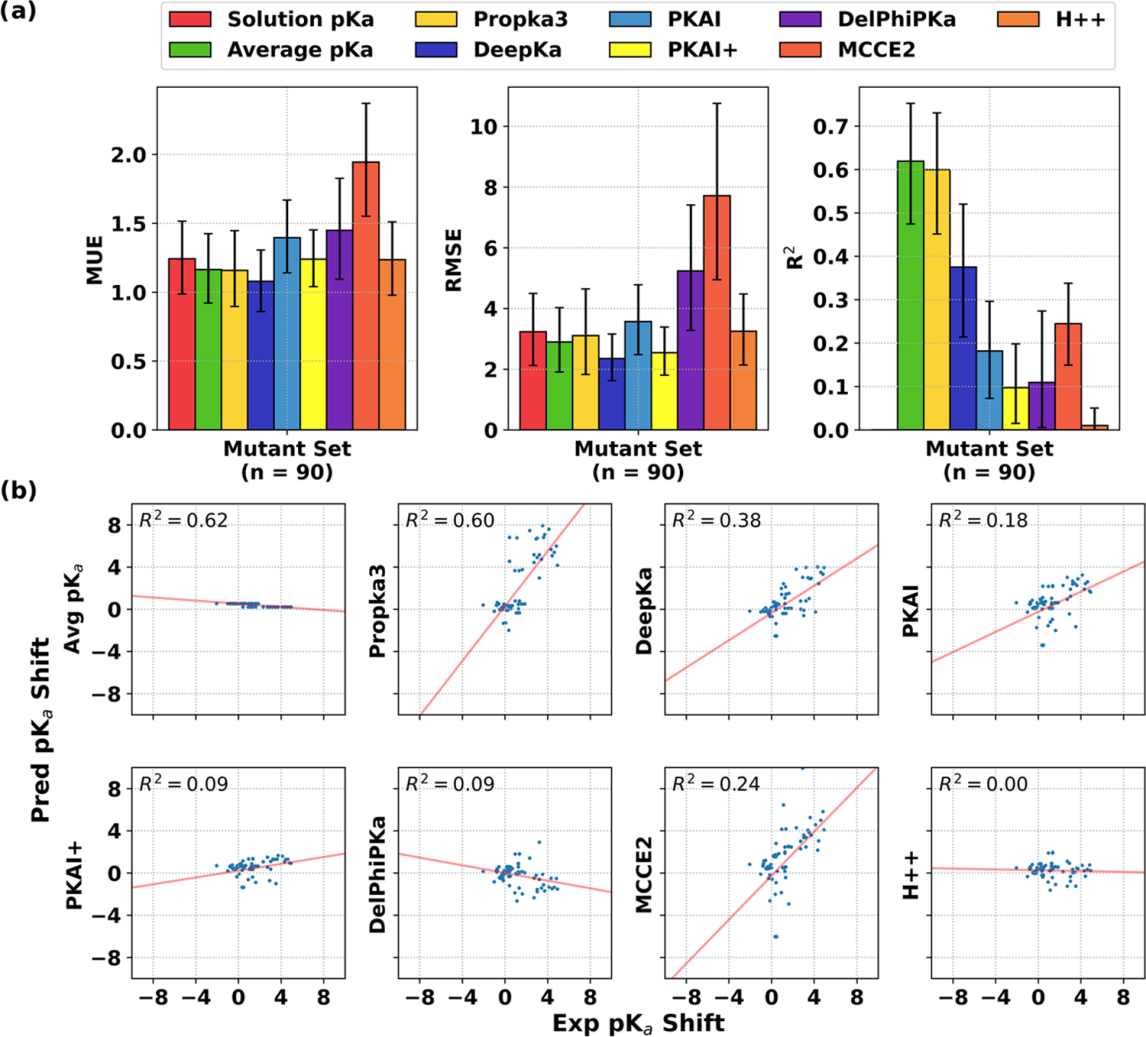
Performance of p*K*_a_ predictors on modeled protein mutant structures. The modeling of the Mutant Set consisting of 90 site-directed mutants with experimental p*K*_a_ data for Glu and His residue types is described in the Methods section. Error bars denote 95% confidence intervals. (a) Error metrics and correlation coefficients. (b) Correlation plots between experimental and predicted p*K*_a_ shifts.

With the above-noted caveats about prediction sensitivities to structural variations, it was hypothesized that an evaluation of p*K*_a_ prediction accuracies on entirely modeled protein structures would lead to degraded performances by compounding p*K*_a_ prediction errors with the inevitable structural prediction errors. A more tractable preliminary assessment at this point might be drawn from the PKAD-based single- and double-point structures in the Mutant Set that we curated and which contained 90 entries with available p*K*_a_ measurements for His and Glu residue types (see the Methods section). Starting from the experimental structures of parent proteins, we have modeled the mutated residues, hoping that structural modeling errors would not be too large. The performances of various predictors were summarized in Figures 7 and S12. First, indeed, we observed that the prediction performances deteriorated for modeled mutant structures relative to experimental structures. Second, the empirical methods, PROPKA3 and DeepKa, remained as the best performers in terms of prediction errors. This was more apparent from the correlation scatter plots for p*K*_a_ shifts. On the one hand, *R*^2^ values of 0.35–0.60 and correlation slopes close to 1 were obtained with PROPKA3 and DeepKa, while on the other hand, no or even anticorrelations were obtained with some of the physics-based predictors. Again, these prediction results need to be treated with caution, given the underlying assumption that protein backbone rearrangements upon mutation were relatively restricted and modeled mutant structures were fairly reliable. Nevertheless, assessing p*K*_a_ prediction methods on such a set of modeled mutants is relevant for the aforementioned practical applications in protein and antibody engineering based on site-directed mutagenesis.

## Conclusions

The performances of several high-throughput p*K*_a_ predictors were systematically evaluated on a nonredundant subset of 408 protein p*K*_a_ data from the PKAD set. Computationally efficient approaches were evaluated, which included both empirical methods: PROPKA3, DeepKa, PKAI, and PKAI+; and physics-based methods: DelPhiPKa, MCCE2, and H++. While no predictor significantly outperformed the null hypotheses of using a fixed value, pertaining to solution or average p*K*_a_ for each amino acid type, several methods did offer some benefits. Overall, it was found that DeepKa, PKAI, and H++ offered average classification accuracies of p*K*_a_ shifts of greater than 56% for Asp, His, and Lys residues, which were 10% greater than those of the null models. By contrast, Glu p*K*_a_ shifts prediction and classification accuracies were poor as evaluated by all predictors.

Generally, most methods had similar performances, with methods such as DeepKa, PKAI+, PROPKA3, and H++ attaining slightly better accuracies compared to the rest. The empirical method DeepKa was consistently found to be among the best-performing methods overall, in terms of many metrics including classification accuracy, MUE, and RMSE. Among the group of physics-based methods tested, H++ appeared as the most accurate, but produced many outliers as illustrated by a relatively low MeUE and high RMSE. Unfortunately, statistical bootstrapping of the evaluated methods revealed that current p*K*_a_ predictors could not outperform the null hypotheses with confidence.

Simple arithmetic averaging of these empirical predictors in a consensus approach afforded improved performances, with the two-predictor DeepKa/PROPKA3 consensus emerging as the most transferrable and accurate across many systems. It is hoped that this work provides a basis and useful guide for future structure-based p*K*_a_ calculations in applied research areas such as *in silico* high-throughput screening for engineering of therapeutic proteins, including but not limited to antibodies. Understanding the accuracies of current p*K*_a_ predictors would also allow benchmarking and comparisons to future developments. One limiting factor in the field is the lack of available data for calibration and validation.^40^ The field would benefit greatly if the additional but often ignored, low-cost step of performing titration experiments could be regularly included in NMR structural determination projects once the more involved aspects of isotope labeling and peak assignment have been completed.

## Data Availability

Curated protein structures (PDB files) used as input for p*K*_a_ calculations in this study and associated tabulated information describing these structure files are available in the Supporting Information.

## Supporting Information Available

Impact of internal dielectric constant and salt concentration on predictor accuracies; additional bootstrapped error metrics for various tested predictors and data sets; relationship between predicted p*K*_a_ errors and surface exposure and structural variability; cross-correlations between different methods; performance of consensus methods; outlier analysis; classification performances by individual amino acid residue types; and tabulated values of performance metrics for the predictors evaluated in this study (PDF)
Composition of protein structure sets used in this study including all experimental and predicted p*K*_a_ values (XLSX)
Curated protein structures used as input for p*K*_a_ calculations (ZIP)

## Abbreviations

PB: Poisson–Boltzmann
cpHMD: constant-pH molecular dynamics
QM: quantum mechanical
TI: thermodynamic integration
QM/MM: quantum mechanics/molecular mechanic
MM: molecular mechanics
vHTS: virtual high-throughput screening
ML: machine-learning
PKAD: p*K*_a_ database
*sc*: salt concentration
FF: force field
MUE: mean unsigned error
MSE: mean signed error
RMSD: root-mean-squared error
MeUE: median unsigned error
MeSE: median signed error
AUC: area under the curve
