# Supplementary material for: Comparative Performance of High-Throughput Methods for Protein pKa Predictions
Source: J Chem Inf Model. 2023 Aug 8;63(16):5169–81. doi: 10.1021/acs.jcim.3c00165 (PMC10466379; doi:10.1021/acs.jcim.3c00165)
Supplement: Supplementary file 1 — ci3c00165_si_001.pdf [file ci3c00165_si_001.pdf]

Supplemental Material

for

Comparative Performance of High-Throughput Methods for Protein pK_a Predictions

Wanlei Wei, Hervé Hogues, and Traian Sulea*

Human Health Therapeutics Research Center, National Research Council Canada

6100 Royalmount Avenue, Montreal, QC, H4P 2R2, Canada

* Corresponding author: traian.sulea@nrc-cnrc.gc.ca

Supplemental Text

Impact of Internal Dielectric Constant and Salt Concentration on Macroscopic Predictor Accuracies. For all reported correlations, the predicted pK_a shifts (*i.e.*, $pK_{a\ (predicted)} - pK_{a\ (free\ amino\ acid)}$) were plotted against experimental pK_a shifts (ie. $pK_{a\ (experimental)} - pK_{a\ (free\ amino\ acid)}$). Physics-based methods, namely H++, MCCE2, and DelPhiPKa, were subjected to calculation using various adjustable parameters, including internal dielectric, ε_r, and salt concentration, *sc*, as to identify the best suitable settings. The obtained results indicated that the choice of the internal ε_r constant greatly affected the accuracy of pK_a calculation for H++ and MCCE2 (**Figure S1a and b**). Both methods recorded a high squared correlation coefficient (R^2) when computed at $\varepsilon_r = 4$. It was found that the value of *sc* did not greatly impact R^2 nor mean unsigned error (MUE). For H++, using larger ε_r greatly deteriorated the quality of the correlation. Conversely, the error of the fit, expressed as MUE, was found to decrease for H++ and MCCE2 as ε_r was increased beyond ε_r = 4. It was also found that the error could generally be reduced as *sc* was increased.

In the case of MCCE2, no major reduction in MUE was observed when the *sc* was increased beyond 0.15 M. Parameters in the vicinity of ε_r = 8 and *sc* = 0.15 M seemed to be optimal for MCCE2 with regards to minimizing errors. Surprisingly, H++ seemed to have a completely different set of parameters which minimized MUE. These were of ε_r = 40 and *sc* = 0.30 M. These two conditions were used for MCCE2 and H++, respectively. Interestingly, R^2 values for H++ and MCCE2 were optimal at ε_r = 4 and *sc* = 0 M. Consequently, a simple method recalibration could be performed at these parameter sets to lower their overall errors. Unexpectedly, DelPhiPKa calculations indicated that the predicted pK_as had minimal correlation to that of experiment (**Figure S1c**; **Figure S1**). More specifically, an R^2 of 0.03 was the highest correlation registered. While the MUE of 0.8 was not large, its low R^2 value indicated that that DelPhiPKa was not useful with regards to protein pK_a prediction.

Comparisons to other pK_a prediction methods were performed at the ε_r and *sc* parameter set that was optimal in terms of MUE. This was done for H++ (ε_r = 40 and *sc* = 0.30 M), MCCE2 (ε_r = 8 and *sc* = 0.15 M), and DelPhiPKa (ε_r = 1, *sc* = 0 M, FF = Amber).

Cross Correlation of Various Approaches. To further understand the current state of pK_a predictions, pairwise correlations between methods were calculated. In addition, pairwise cross-correlation could help chose methods to build a consensus pK_a prediction method. Results indicated that certain pairs of methods had relatively high correlation (**Figure S8**). Not surprisingly, PKAI and PKAI+ had an R^2 of 0.92, due to their common methodology and training data. It was also presumed that both these methods correlated highly to other PB-based methods due to their similar training. As expected, PKAI registered an R^2 of 0.72 to H++, and an R^2 of 0.50 to MCCE2. However, the correlation of PKAI to DelPhiPKa was lower, with an R^2 of only 0.32. It was also expected that the PB based methods would correlate with each other, which was true for H++ and MCCE2, with an R^2 of 0.50. Again, their correlation to DelPhiPKa was noticeably lower, with an R^2 of 0.32 and 0.1, respectively. In our benchmarking, DelPhiPKa had poor correlation to experimental data (**Figure S1**, and **Figures 2** and **3**), which might partially help to explain this phenomenon.

Performance of Consensus Methods by Residues Type. His pK_a prediction originally favoured DeepKa and H++. Consensus methods did improve these predictions. Consensus-1 offered the highest accuracy with RMSE, MUE, MeUE and R^2 of 0.82, 0.65, 0.46 and 0.43, respectively,

which meaningfully outperformed both DeepKa and H++ across all metrics (**Figure 5** and **Figure S9**). The second-best consensus method, consensus-4, also yielded greater accuracy than DeepKa, especially with regards to MeUE. The former achieved RMSE, MUE, MeUE and R^2 of 0.89, 0.68, 0.53 and 0.42, respectively. However, its MeUE was slightly higher than that of H++. Consensus-2 performed similarly to DeepKa with RMSE, MUE, MeUE and R^2 of 0.91, 0.69, 0.59 and 0.44, respectively. Overall, consensus-1 performed most accurately for His pK_a prediction followed sequentially by consensus-4 and -2.

Lys pK_a prediction originally favoured both DeepKa and PROPKA3 for their respective strengths. The former achieved a low MUE while the latter yielded lower RMSE and MeUE. The consensus-2 approach was able to improve these results, by combining them, which produced RMSE, MUE, MeUE and R^2 of 0.69, 0.55, 0.40 and 0.64, respectively (**Figure 5** and **Figure S9**). This amalgamation produced a higher overall accuracy than either constituent method. The accuracy of other consensus methods performed slightly worse, including consensus-4. The latter scored RMSE, MUE, MeUE and R^2 of 0.77, 0.59, 0.41 and 0.63, respectively. Consensus-2 slightly outperformed DeepKa.

Asp pK_a prediction was previously found to favour DeepKa and PROPKA3. Between these two methods, DeepKa was found to yield lower RMSE and MUE, while PROPKA3 produced lower MeUE. The application of consensus methods for Asp pK_a prediction further lowered these error metrics and was successful. More specifically, consensus-2 produced RMSE, MUE, MeUE and R^2 of 0.84, 0.57, 0.42 and 0.52, respectively and consensus-4 yielded 0.87, 0.57, 0.34 and 0.54, respectively (**Figure 5** and **Figure S9**). As a result, consensus-2 was observed to outperform DeepKa across all metrics. Similarly, consensus-4 surpassed PROPKA3 across all metrics. A comprehensive inspection of error metrics showed that consensus-4 seemed to surpass consensus-2 with regards to predicting Asp pK_as. This is because consensus-4 significantly surpassed consensus-2 in MeUE. However, their RMSEs were almost identical. In summary, Asp pK_a prediction seemed to be most accurate when employing consensus-4.

Glu pK_a prediction was hitherto one of the most difficult. In fact, the two null models were found to be superior to the benchmarked predictors across all error metrics (e.g., RMSE, MUE, MeUE and protonation errors). This was primarily due to Glu sidechain carboxylate being less shifted compared to other titratable residues. Remarkably, all consensus methods, except for consensus-1, was able to outperform the null models. Furthermore, consensus-2 and -4 were notable as they comfortably surpassed the null-2 model. RMSE, MUE, MeUE and R^2 of 0.67, 0.46, 0.31 and 0.28 were achieved by the former, respectively, while 0.66, 0.47, 0.35 and 0.26 were obtained by the latter, respectively (**Figure 5** and **Figure S9**). In summary, Glu pK_a prediction seemed to be most accurate when employing consensus-2 or -4.

Examples of Common Outliers. Titratable sidechain groups which were incorrectly predicted by many pK_a predictors were flagged for visual inspection (**Table S7**). In this case, an incorrect prediction was defined as a difference of greater than one pK_a unit from that of the experiment. During the pK_a prediction of His-60 imidazole group of family 11 xylanase (PDB: 1H4G),[1] no method was successful in fulfilling this criterion. Structurally, the $_{His60}NE_2$ of interest is hydrogen-bonded (H-bonded) to a nearby $_{Glu167}OE_1$. In addition, an Arg-164 is also present, but slightly further away at 4.6 Å ($_{His60}CE_1\cdots{}_{Arg164}NH_1$). Due to the closer and more involved association of the Glu-167 than Arg-164, it was expected that the imidazole pK_a would be slightly elevated or unchanged. Unexpectedly, it was found that its pK_a of 4.01 was significantly suppressed. While most methods did in fact correctly predict a lowering of the pK_a, no method was successful in

estimating the magnitude of that change. DeepKa predicted a pK_a of 5.33, which was the lowest of these methods.

One striking feature of the binding pocket was the presence of three nearby methionine residues (Met-66, Met-172 and Met-176), which is rare in proteins and suggests a functional role. In fact, in conjunction with histidine, multiple methionines have previously been observed to coordinate metal cations, such as copper.[2] While copper cation has not been confirmed to exist in this case, it does highlight the importance of the accuracy of the used model. The possible presence of a copper cation could explain the lower pK_a of the His-60. As structure-based pK_a predictors rely on a robust structure, they are extremely sensitive to any structural inaccuracies.

In another case, His-72 of bovine heart phosphotyrosyl phosphatase (PDB: 1PNT)[3] was surrounded by several H-bond acceptors, composed of $_{Glu23}COO^-$, $_{Asp42}COO^-$, $_{Ser43}$=O, and $_{Lys73}$=O. Furthermore, there were three additional functional groups which were positionally ambiguous by X-ray crystallography. More specifically, the positions of the protons in $_{Ser43}OH$ and $_{Thr70}OH$ were not observable while $_{Asn15}CONH_2$ was isoelectronic. Consequently, both functional groups could act as a H-bond acceptor or donor, depending on their orientations. Consequently, energetic sampling during pK_a prediction would be required for these residues. Nevertheless, due to the overall prevalence of H-bond acceptors, the pK_a of His-72 could be visually rationalized to be significantly elevated. Indeed, a pK_a of 9.19 was registered experimentally. Unexpectedly however, no predictor was able to correctly predict the magnitude of this shift. Exacerbating this trend, both DeepKa and PROPKA3 predicted a reduction in the pK_a value. The sole exception was MCCE2, which predicted a value of 8.57, which was within 1 pK_a unit of the experiment.

In a separate case, the pK_a prediction of Lys-39 of MutT protein (PDB: 1MUT)[4] failed for all methods. A visualization of the various 15 NMR-based conformational states revealed that Lys-39 and surrounding residues were extremely flexible. While Lys-39 made more frequent contacts with Glu-41, other residues were also found to transiently interact with Lys-39. Impressively, Arg-52 was found to move from over 20 Å away to less than 6 Å from Lys-39, which could be the reason for the depressed p*K*a of 8.4 for the latter. For structure-based pK_a calculations, these drastic changes in amino acid positions will present a significant challenge.

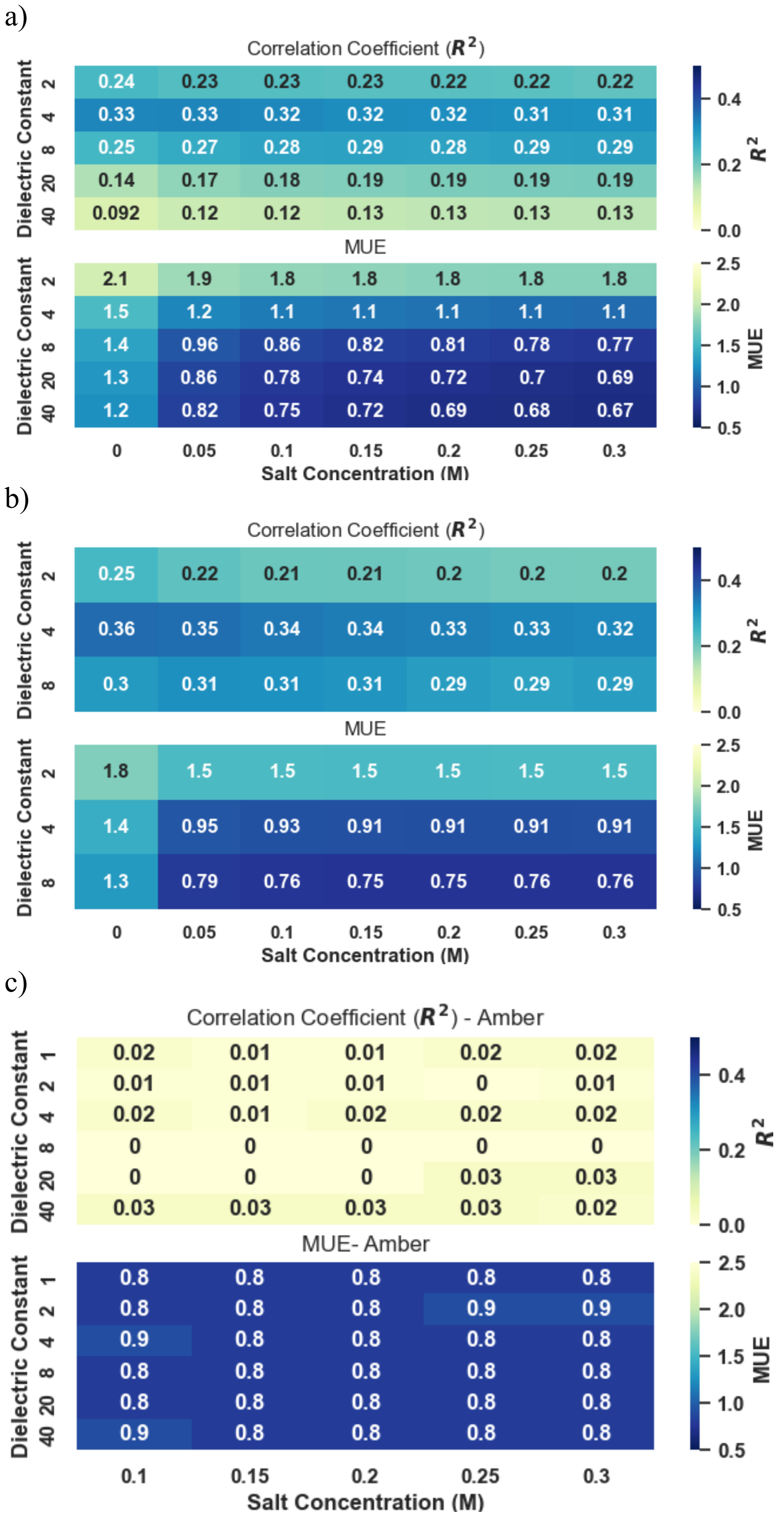

Figure S1. The accuracy of a) MCCE2, b) H++, and c) DelPhiPKa at various dielectric constants and salt concentrations on the Large Set, as represented by: R^2 (top) and MUE (bottom).

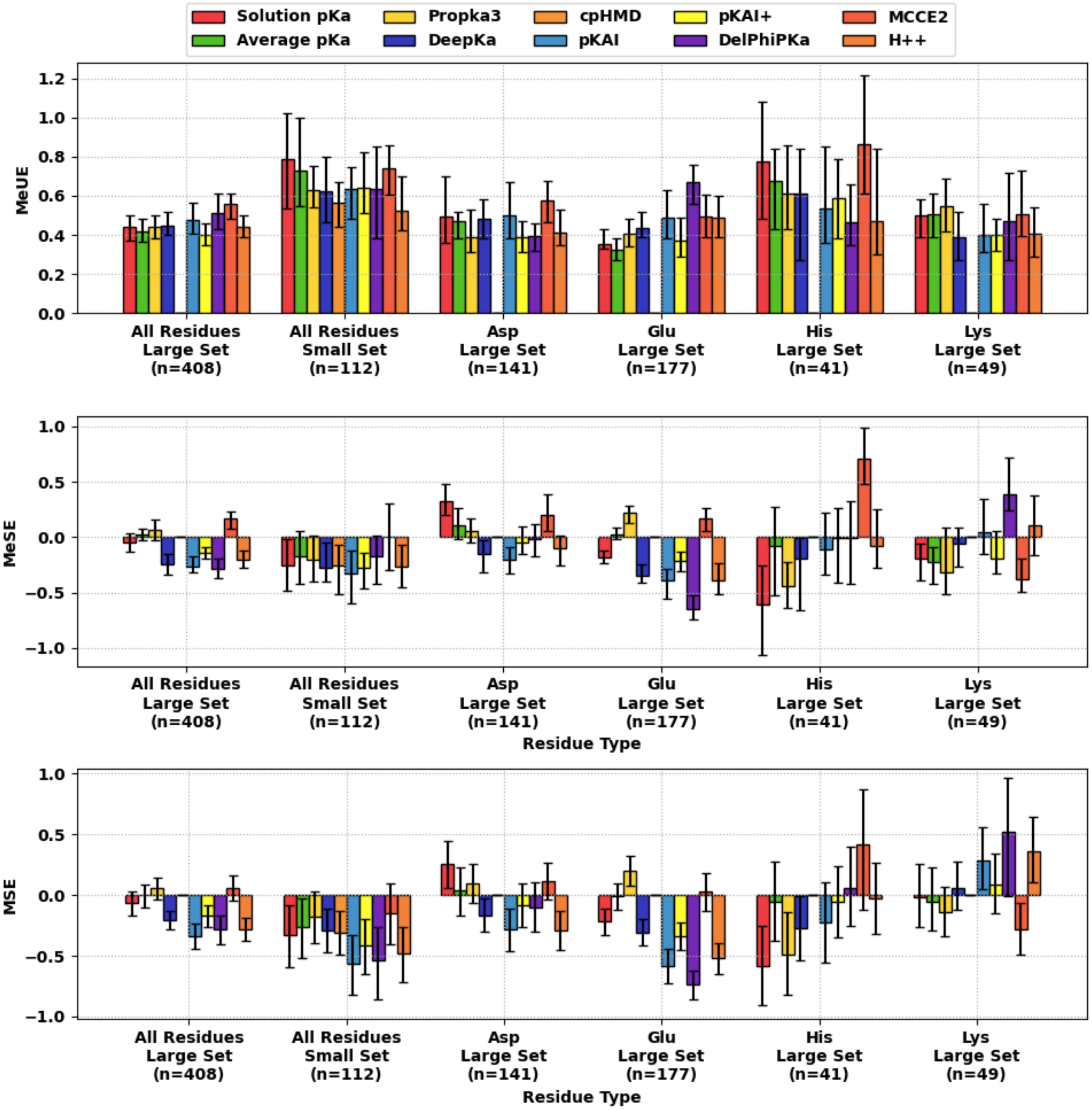

Figure S2. Additional bootstrapped performance metrics for tested pK_a predictors on various sets and subsets of pK_a data. Error bars denote 95% confidence intervals. Null models were estimated using solution pK_a and average pK_a.

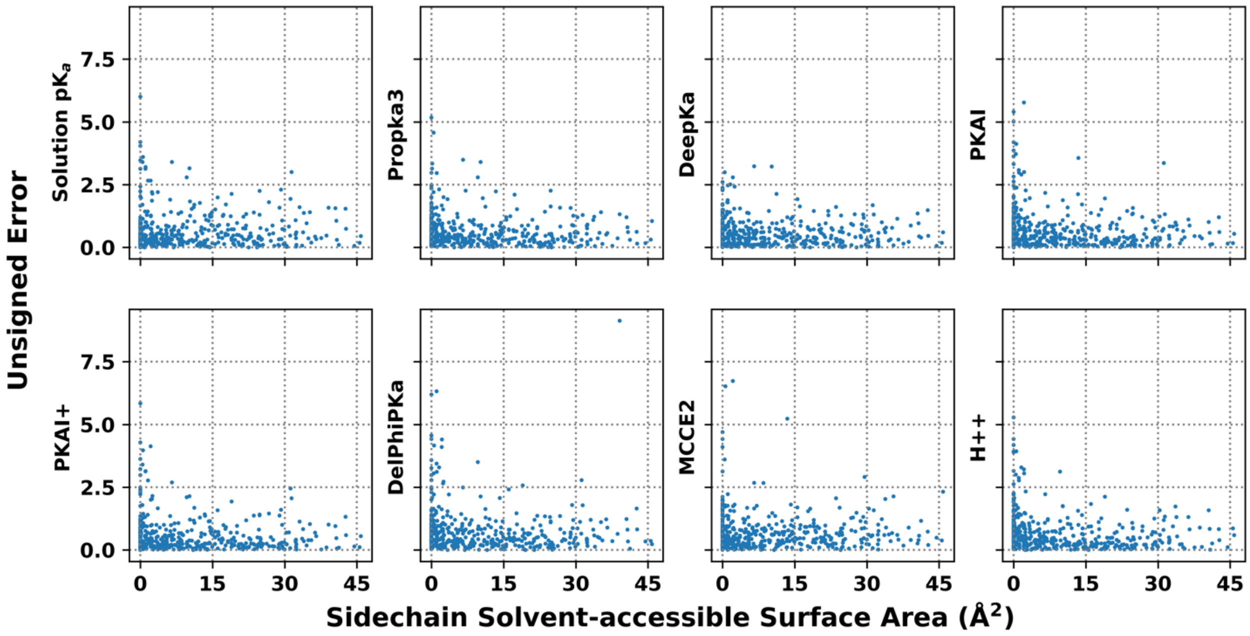

Figure S3. Relationship of pK_a errors to solvent-accessible surface areas (SASA) of ionizable residue sidechains in the Large Set for various tested predictors.

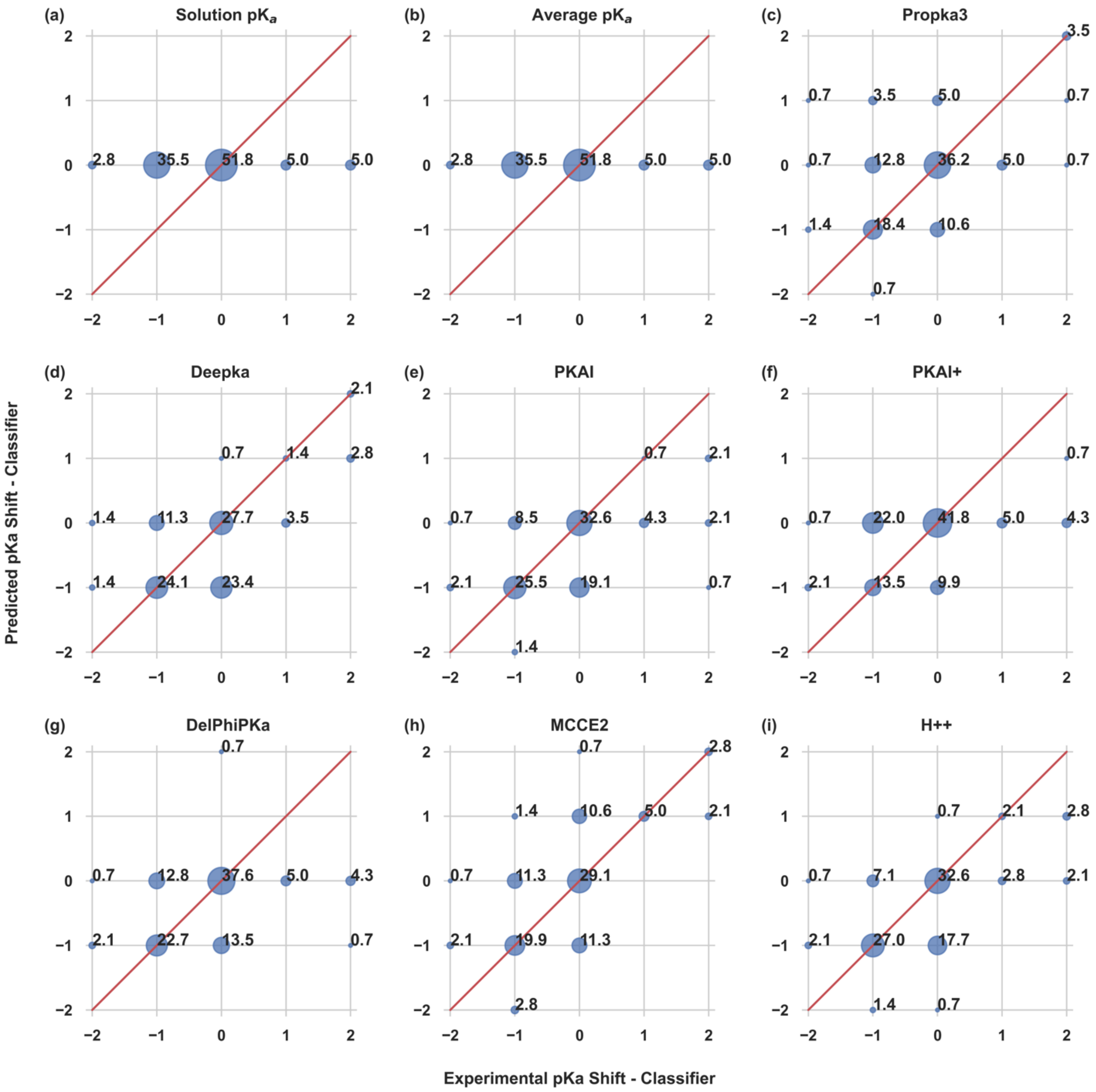

Figure S4. Quantized estimates for the direction and magnitude of pK_a shifts are shown as percentages (%) for **Asp**. The overall accuracy of each method is equalled to the sum along the diagonal line. Quantized accuracies are shown for: a) solution p*K*a (51.8%), b) average pK_a (51.8%), c) PROPKA3 (58.1%), d) DeepKa (55.3%), e) PKAI (58.8%), f) PKAI+ (55.3%), g) DelPhiPKa (60.3%) h) MCCE2 (56.8%), and i) H++ (61.7%).

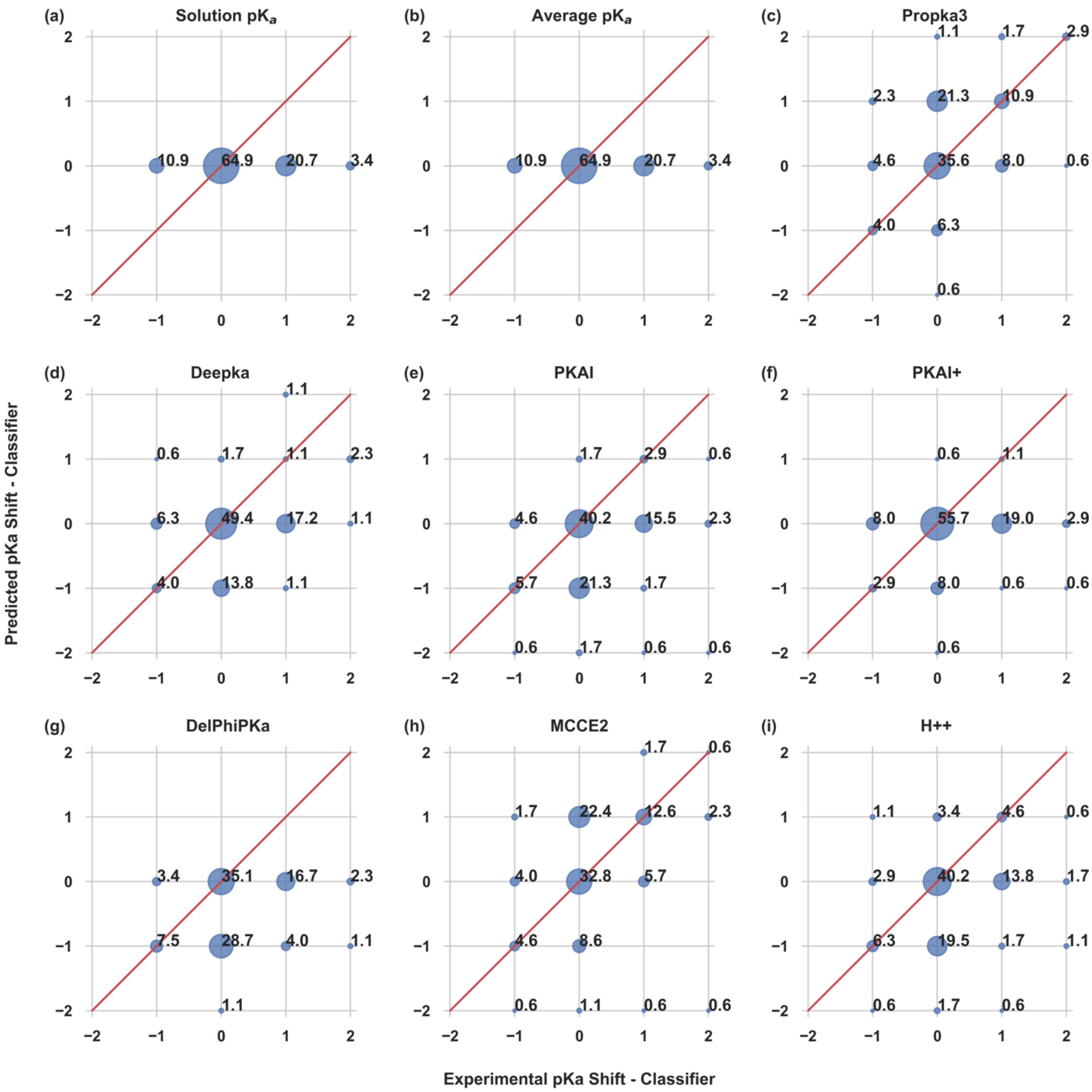

Figure S5. Quantized estimates for the direction and magnitude of pK_a shifts are shown as percentages (%) for **Glu**. The overall accuracy of each method is equalled to the sum along the diagonal line. Quantized accuracies are shown for: a) solution p*K*a (64.9%), b) average pK_a (64.9%), c) PROPKA3 (53.4%), d) DeepKa (54.5%), e) PKAI (48.8%), f) PKAI+ (59.7%), g) DelPhiPKa (42.6%) h) MCCE2 (50.6%), and i) H++ (51.1%).

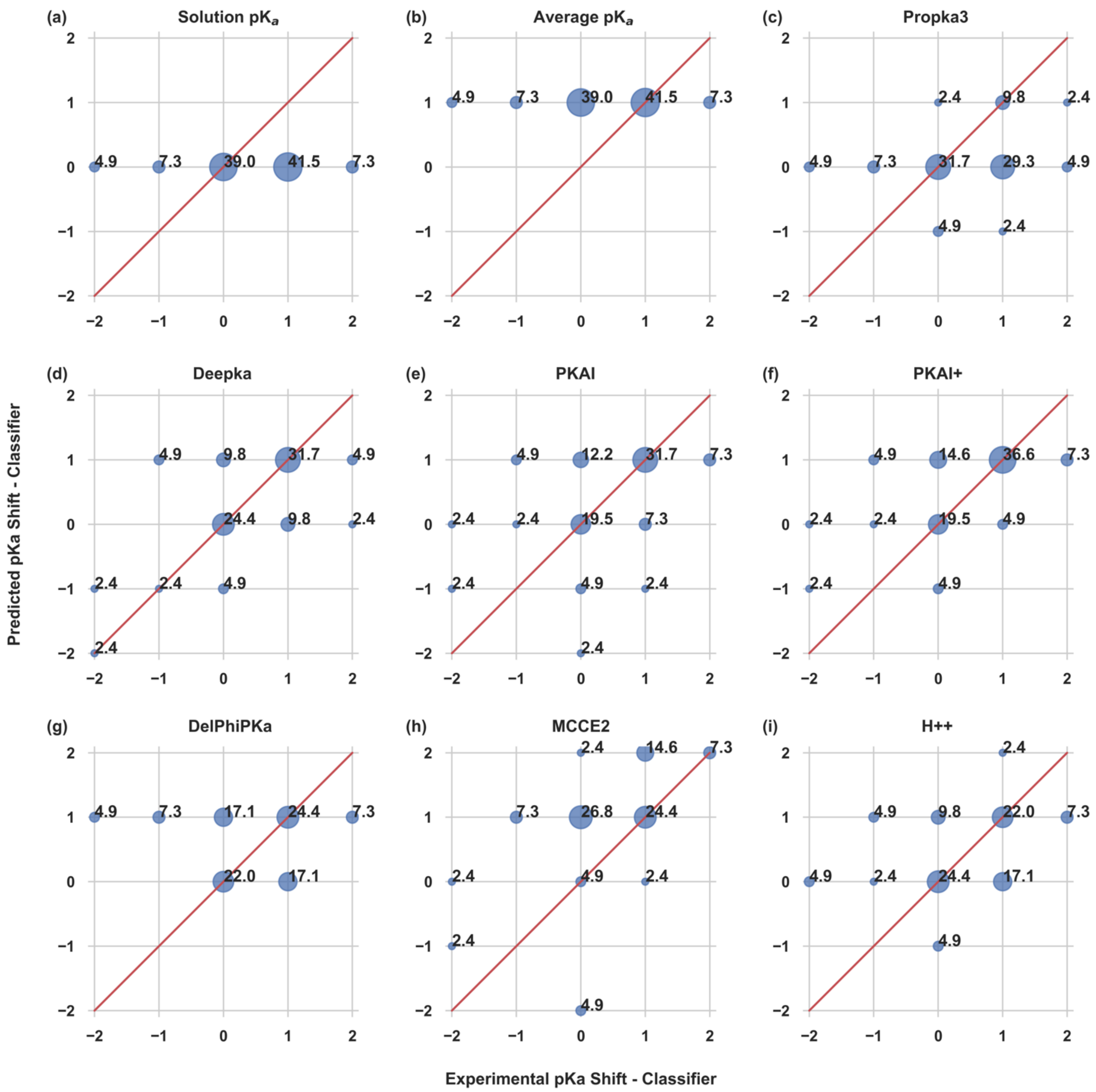

Figure S6. Quantized estimates for the direction and magnitude of pK_a shifts are shown as percentages (%) for **His**. The overall accuracy of each method is equalled to the sum along the diagonal line. Quantized accuracies are shown for: a) solution p*K*a (39.0%), b) average pK_a (41.5%), c) PROPKA3 (41.5%), d) DeepKa (60.9%), e) PKAI (51.2%), f) PKAI+ (56.1%), g) DelPhiPKa (46.4%) h) MCCE2 (36.6%), and i) H++ (46.4%).

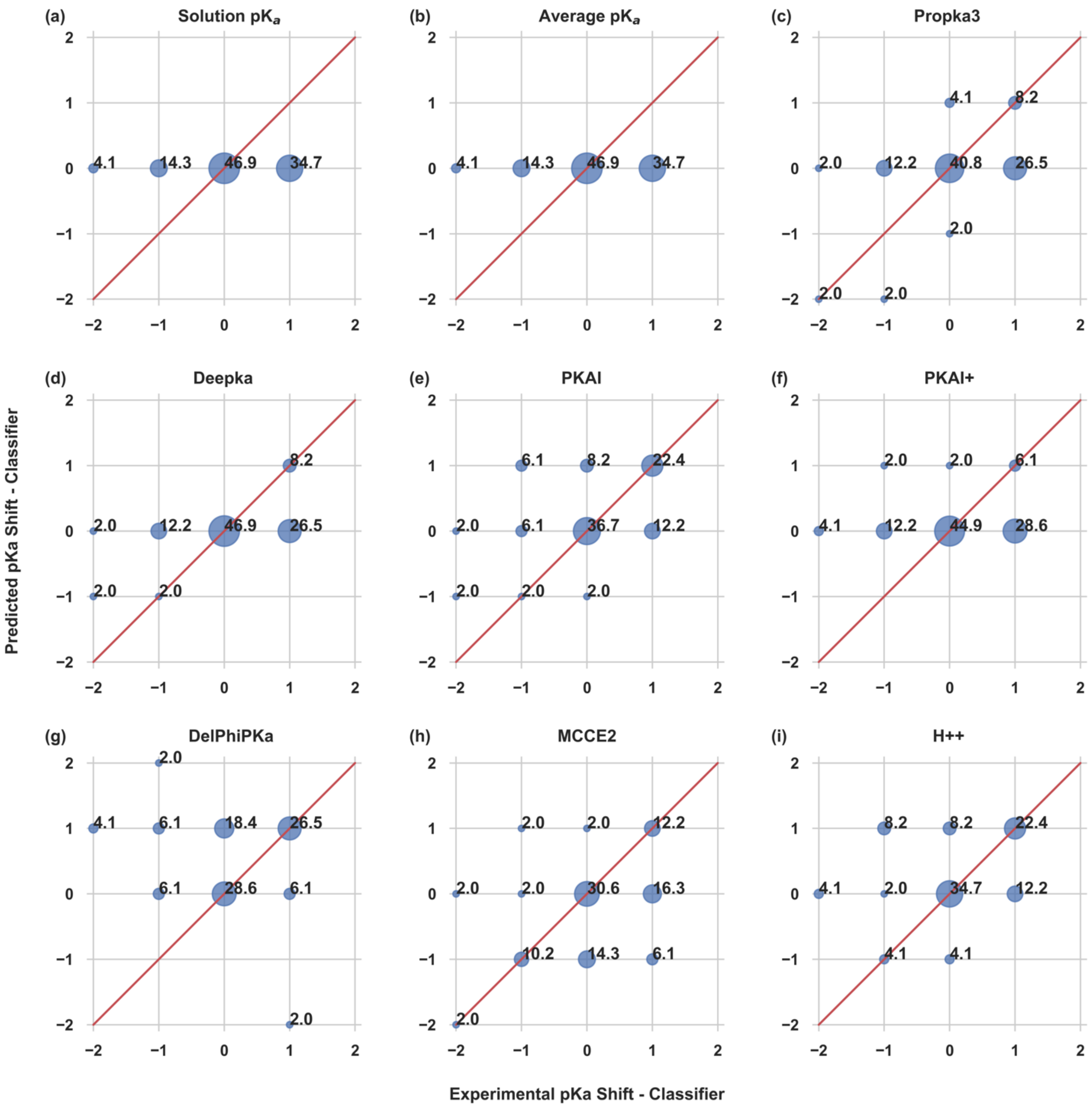

Figure S7. Quantized estimates for the direction and magnitude of pK_a shifts are shown as percentages (%) for **Lys**. The overall accuracy of each method is equalled to the sum along the diagonal line. Quantized accuracies are shown for: a) solution p*K*a (46.9%), b) average pK_a (46.9%), c) PROPKA3 (51.0%), d) DeepKa (57.1%), e) PKAI (61.1%), f) PKAI+ (51.0%), g) DelPhiPKa (55.1%) h) MCCE2 (55.0%), and i) H++ (61.2%).

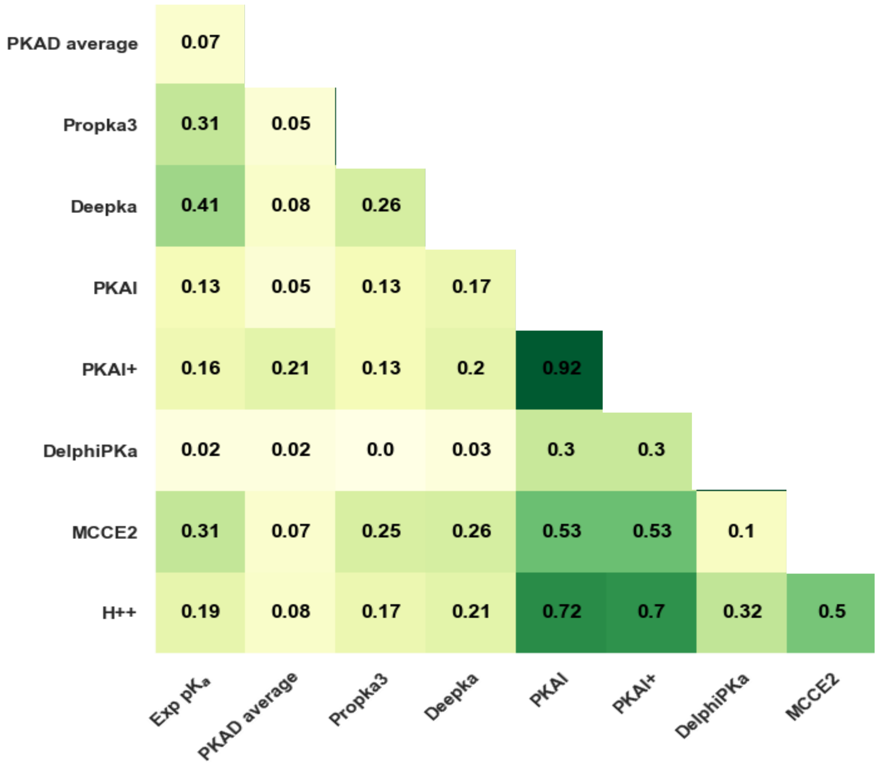

Figure S8. Pairwise squared Pearson correlation coefficient (R^2) between different pK_a predictors when applied to the Large Set. The PKAD average (null-2) model was included as a comparison.

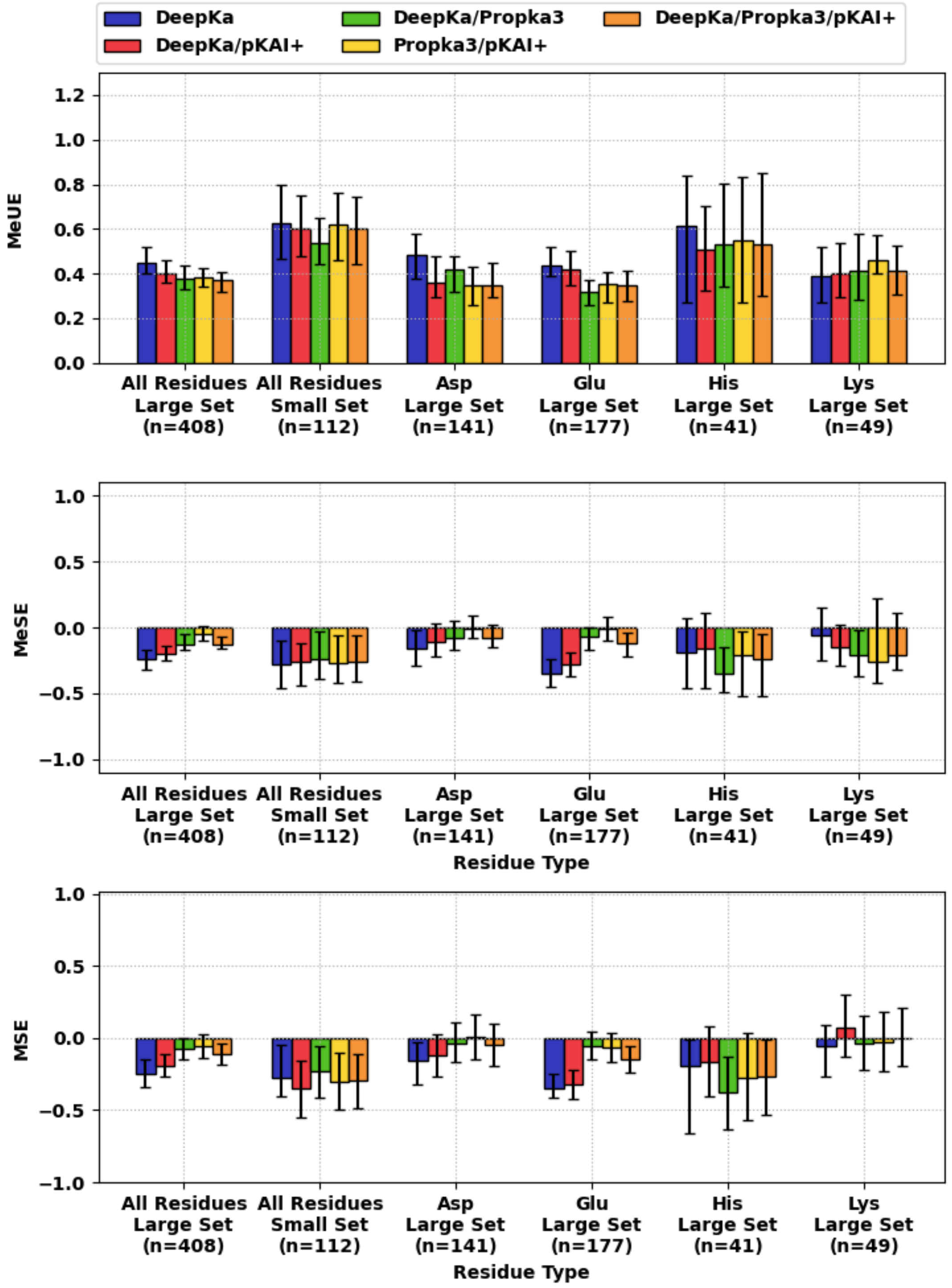

Figure S9. Additional bootstrapped errors metrics for the consensus approach combining empirical pK_a predictors. Data for DeepKa predictions are included as reference. Error bars denote 95% confidence intervals.

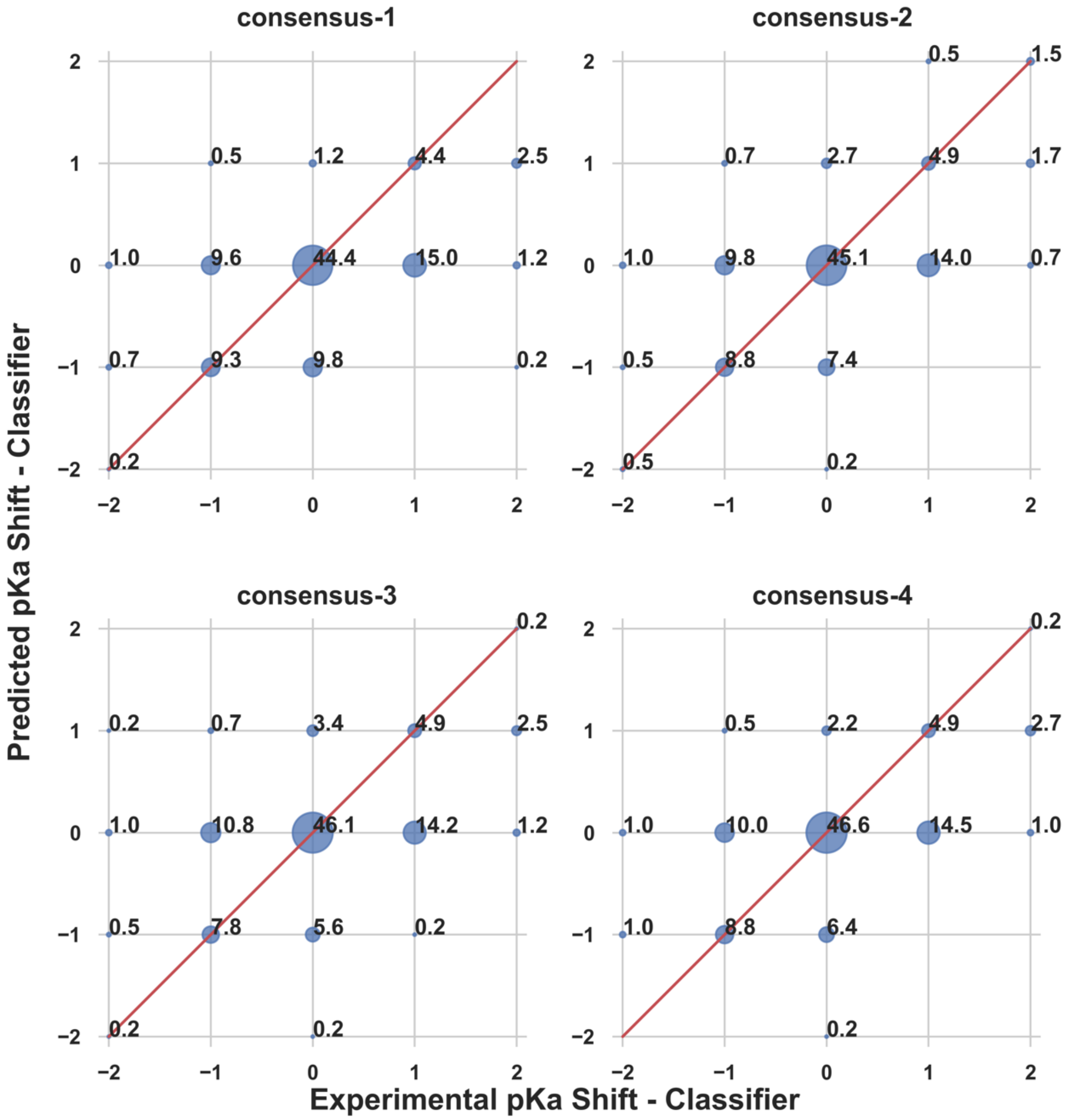

Figure S10. Quantized estimates for the direction and magnitude of pK_a shifts are shown as percentages (%) for the Large Set. The overall accuracy of each method is equalled to the sum along the diagonal line. Quantized accuracies are shown for: a) consensus-1: DeepKa/PKAI+ (58.0%), b) consensus-2: DeepKa/PROPKA3 (60.8%), c) consensus-3: PROPKA3/PKAI+ (59.2%), and d) consensus-4: DeepKa/PROPKA3/PKAI+ (60.7%).

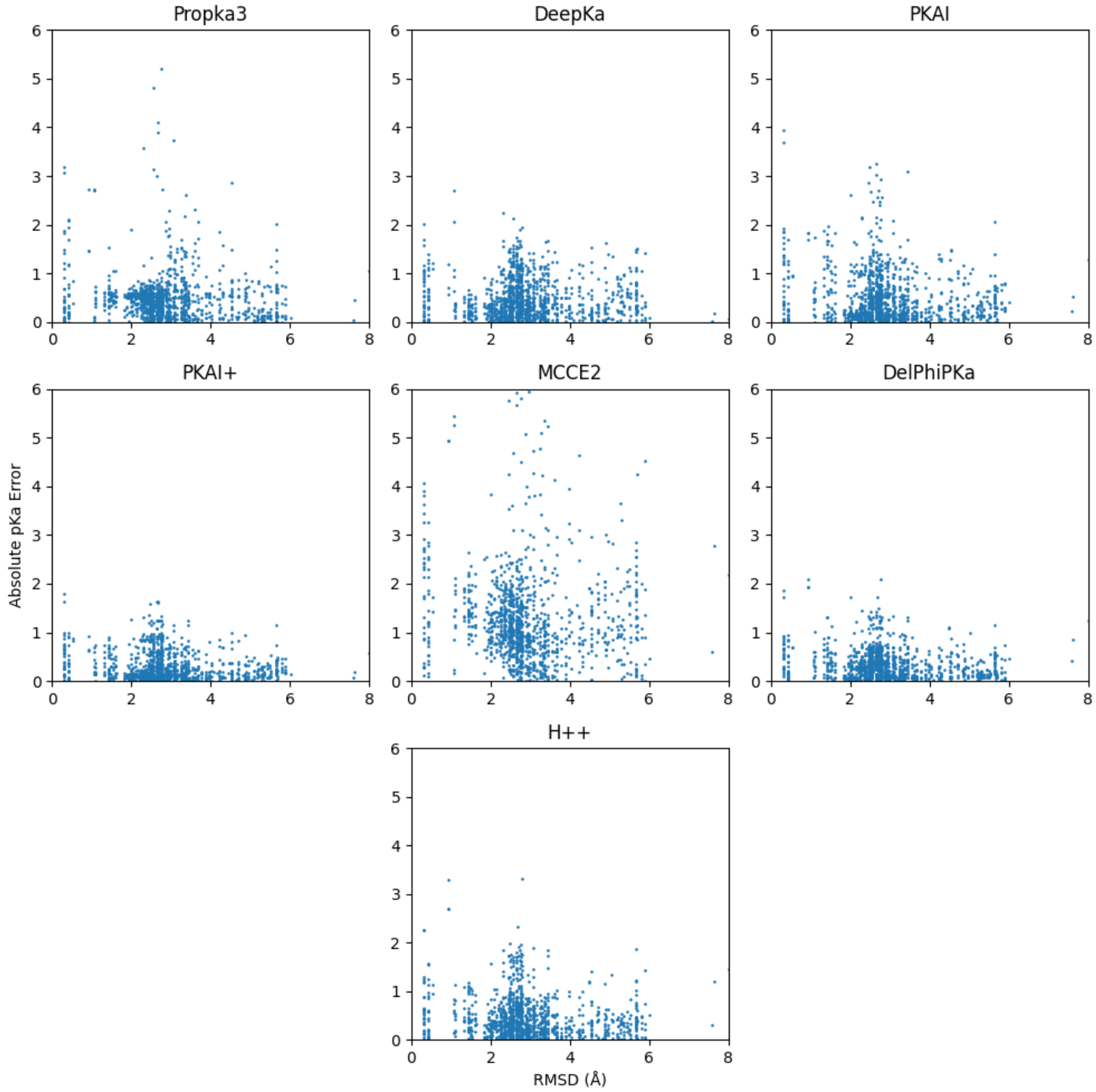

Figure S11. Relationship between calculated unsigned pK_a shift errors and structural variability expressed as the root-mean-square deviation (RMSD, in Å) for each structure in a protein ensemble experimentally determined (NMR ensemble or multiple X-ray structures). For each ensemble, the average structure was generated and used as reference for RMSD calculations. Overall, there were 16 conformational ensembles for different proteins, totalling 178 conformational states and 1188 pK_a data points included in each scatter plot.

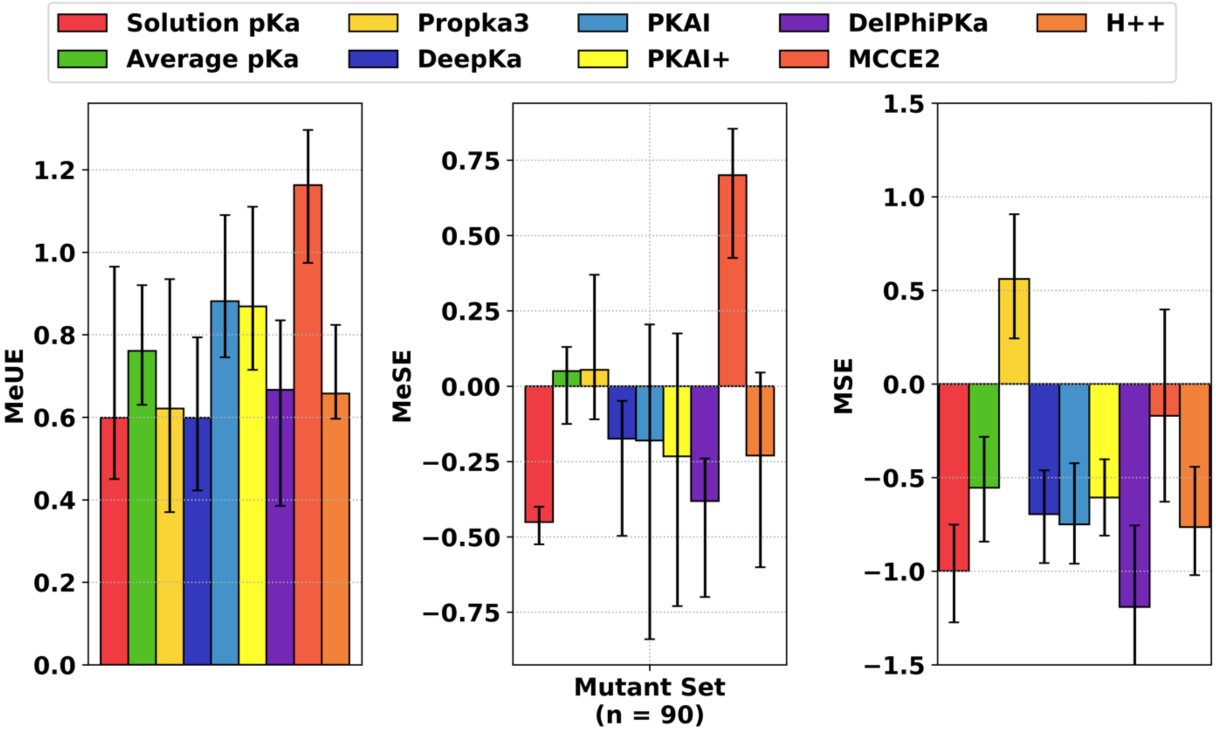

Figure S12. Additional bootstrapped error metrics of tested pK_a predictors on the Mutant Set. Error bars denote 95% confidence intervals.

Table S1. Experimental and predicted pK_a values from the Large Set (n=408).

PDB	Residue	SASA (Å²) side chain	Expt p*K*a	Soln p*K*a	Avg p*K*a	Propka3	DeepKa	PKAI	PKAI+	DelPhiPKa	MCCE2	H++
135l	GLU7	14.86	2.68	4.07	4.28	2.94	3.32	2.74	3.51	2.85	3.04	2.95
135l	GLU35	16.07	6.06	4.07	4.28	6.78	4.43	4.69	4.48	3.65	5.22	4.23
135l	ASP18	21.43	2.68	3.90	3.68	2.94	2.79	1.94	2.85	2.79	2.06	2.28
135l	ASP52	0.00	3.78	3.90	3.68	5.23	4.72	3.75	3.80	3.53	4.87	3.91
135l	ASP87	12.38	2.13	3.90	3.68	2.48	3.18	3.09	3.42	3.03	3.28	2.70
135l	ASP119	17.71	3.35	3.90	3.68	3.39	2.54	2.68	3.17	2.94	3.20	2.82
1a2p, 1fw7, 1bnr, 1bnj 1bni	HIS18	17.78	7.75	6.04	6.57	6.23	7.00	6.90	6.84	6.73	6.21	6.92
1a2p, 1fw7, 1bnr, 1bnj 1bni	HIS102	4.86	6.30	6.04	6.57	5.82	6.43	6.25	6.50	6.63	6.80	6.23
1a2p, 1fw7, 1bnr, 1bnj 1bni	GLU29	1.84	3.75	4.07	4.28	3.97	3.76	4.17	4.18	3.96	4.16	4.21
1a2p, 1fw7, 1bnr, 1bnj 1bni	GLU60	29.47	3.00	4.07	4.28	4.26	3.62	3.79	4.00	4.19	3.87	3.70
1a2p, 1fw7, 1bnr, 1bnj 1bni	GLU73	0.13	2.10	4.07	4.28	5.24	3.59	2.27	3.26	2.96	2.30	1.37
1a2p, 1fw7, 1bnr, 1bnj 1bni	ASP8	1.33	3.10	3.90	3.68	3.54	2.88	3.21	3.51	3.26	3.17	3.49
1a2p, 1fw7, 1bnr, 1bnj 1bni	ASP12	1.18	3.80	3.90	3.68	3.82	3.99	3.61	3.70	3.66	4.16	3.96
1a2p, 1fw7, 1bnr, 1bnj 1bni	ASP22	16.85	3.30	3.90	3.68	3.65	3.54	3.40	3.59	3.75	3.76	3.51
1a2p, 1fw7, 1bnr, 1bnj 1bni	ASP44	22.15	3.60	3.90	3.68	3.77	3.61	3.60	3.70	4.11	4.15	3.64
1a2p, 1fw7, 1bnr, 1bnj 1bni	ASP54	0.44	2.20	3.90	3.68	4.40	2.31	2.74	3.27	3.12	2.29	2.23
1a2p, 1fw7, 1bnr, 1bnj 1bni	ASP75	0.04	3.10	3.90	3.68	5.58	3.68	1.58	2.63	3.23	1.15	2.28
1a2p, 1fw7, 1bnr, 1bnj 1bni	ASP86	0.00	4.20	3.90	3.68	4.08	3.31	3.26	3.53	3.80	4.10	3.32
1a2p, 1fw7, 1bnr, 1bnj 1bni	ASP101	6.20	2.00	3.90	3.68	2.01	2.36	2.98	3.35	3.43	2.86	2.56
1a91	GLU2	0.98	5.50	4.07	4.28	4.59	3.70	4.20	4.19	4.03	4.92	4.06
1a91	GLU37	1.54	5.50	4.07	4.28	4.57	4.10	4.00	4.10	3.78	4.79	4.10
1a91	ASP7	0.43	5.60	3.90	3.68	4.04	4.42	3.81	3.81	3.96	4.66	4.01
1a91	ASP44	11.22	5.60	3.90	3.68	3.98	3.47	4.01	3.92	3.97	4.57	4.32
1a91	ASP61	1.10	7.10	3.90	3.68	4.14	4.63	4.12	3.96	3.99	4.88	4.33
1ans	GLU20	22.00	5.40	4.07	4.28	4.59	4.12	4.25	4.22	4.04	5.60	4.39
1beo, 1beg	LYS61	0.60	10.10	10.54	10.51	10.31	10.83	10.44	10.44	11.12	9.76	10.39

1beo, 1beg	LYS94	0.51	9.40	10.54	10.51	10.34	10.63	10.87	10.67	11.61	9.97	11.19
1beo, 1beg	ASP21	6.37	2.49	3.90	3.68	3.37	3.07	3.18	3.48	3.43	2.59	3.19
1beo, 1beg	ASP30	23.59	2.51	3.90	3.68	3.93	4.12	3.96	3.88	3.85	4.57	4.11
1beo, 1beg	ASP72	1.36	2.61	3.90	3.68	3.71	3.63	3.33	3.55	3.57	3.56	3.34
1bhc, 4pti, 1d0d, 1bpi	GLU7	5.97	3.70	4.07	4.28	4.48	3.65	3.62	3.88	3.33	4.32	3.69
1bhc, 4pti, 1d0d, 1bpi	GLU49	10.20	3.80	4.07	4.28	3.52	3.74	2.72	3.42	3.61	2.95	2.05
1bhc, 4pti, 1d0d, 1bpi	LYS15	27.84	10.60	10.54	10.51	10.26	10.60	10.54	10.46	11.00	9.12	10.10
1bhc, 4pti, 1d0d, 1bpi	LYS26	44.48	10.60	10.54	10.51	10.43	10.66	10.45	10.46	10.94	10.11	10.49
1bhc, 4pti, 1d0d, 1bpi	LYS41	13.31	10.80	10.54	10.51	10.60	10.53	10.96	10.70	11.41	10.70	10.77
1bhc, 4pti, 1d0d, 1bpi	LYS46	30.41	10.60	10.54	10.51	10.02	10.57	10.29	10.38	10.84	8.95	10.00
1bhc, 4pti, 1d0d, 1bpi	ASP3	14.75	3.00	3.90	3.68	3.77	3.08	3.51	3.66	3.63	3.66	3.36
1bhc, 4pti, 1d0d, 1bpi	ASP50	0.91	3.40	3.90	3.68	4.72	3.44	3.08	3.54	3.14	2.45	1.73
1bus, 2bus	GLU9	21.04	4.50	4.07	4.28	4.90	4.00	4.34	4.27	3.63	4.85	4.39
1bus, 2bus	GLU20	21.43	4.40	4.07	4.28	3.91	3.44	3.49	3.83	3.20	4.13	3.68
1bus, 2bus	ASP6	9.73	4.10	3.90	3.68	3.75	3.32	3.77	3.78	3.67	3.77	3.28
1bus, 2bus	ASP12	25.14	3.60	3.90	3.68	3.89	3.57	3.64	3.72	3.59	3.99	3.51
1cdc	GLU29	0.00	4.42	4.07	4.28	-0.75	3.31	-0.59	1.93	1.44	0.00	0.00
1cdc	GLU33	2.28	4.16	4.07	4.28	4.33	3.77	3.02	3.49	3.13	4.28	2.68
1cdc	GLU41	2.14	6.73	4.07	4.28	6.36	3.94	0.96	2.60	2.63	0.00	3.52
1cdc	GLU56	2.42	3.92	4.07	4.28	3.34	3.27	2.90	3.51	2.62	4.35	3.21
1cdc	GLU99	45.58	4.25	4.07	4.28	4.56	4.07	4.08	4.14	3.89	4.63	3.39
1cdc	ASP25	22.61	3.53	3.90	3.68	3.73	3.06	3.49	3.66	3.72	4.01	3.71
1cdc	ASP26	0.00	3.58	3.90	3.68	4.08	3.44	3.66	3.76	3.72	4.26	3.75
1cdc	ASP28	3.21	3.57	3.90	3.68	3.91	3.40	2.77	3.22	3.28	3.81	2.51
1cdc	ASP62	0.00	4.15	3.90	3.68	4.61	3.84	2.43	3.13	3.65	4.36	4.28
1cdc	ASP71	18.96	3.18	3.90	3.68	3.75	3.90	3.40	3.59	3.73	4.24	3.44
1cdc	ASP72	0.00	4.14	3.90	3.68	4.43	4.06	3.53	3.68	3.55	2.35	2.92
1cdc	ASP94	0.00	3.87	3.90	3.68	3.25	3.60	2.46	3.00	3.47	3.16	2.99
1cvo, 1kxi	GLU17	24.48	4.00	4.07	4.28	4.53	3.71	4.11	4.17	3.87	4.72	4.03

1cvo, 1kxi	ASP42	18.22	3.20	3.90	3.68	3.39	3.46	3.35	3.55	3.36	3.30	3.17
1cvo, 1kxi	ASP59	11.10	2.30	3.90	3.68	2.92	3.04	3.10	3.47	3.15	2.21	2.59
1de3	HIS35	27.12	6.30	6.04	6.57	5.88	7.19	6.83	6.81	6.65	7.59	6.51
1de3	HIS36	21.36	6.80	6.04	6.57	6.49	6.93	7.01	6.90	6.80	7.77	6.99
1de3	HIS50	0.00	7.70	6.04	6.57	5.35	6.70	6.26	6.49	6.64	7.89	6.69
1de3	HIS82	10.35	7.30	6.04	6.57	6.41	7.23	7.67	7.16	7.50	8.77	8.17
1de3	HIS92	1.50	6.90	6.04	6.57	5.63	6.24	5.73	6.21	6.23	6.57	5.93
1de3	HIS104	0.00	6.60	6.04	6.57	6.35	5.91	6.66	6.68	6.65	6.57	6.47
1de3	HIS137	0.00	5.80	6.04	6.57	5.19	5.74	5.46	6.08	6.24	5.79	6.38
1de3	HIS150	40.54	7.60	6.04	6.57	6.40	6.25	6.59	6.65	6.37	7.34	6.02
1de3	GLU19	0.00	4.60	4.07	4.28	4.45	3.59	3.70	3.95	3.41	4.39	3.73
1de3	GLU31	0.00	4.60	4.07	4.28	4.53	3.50	3.32	3.77	3.36	3.92	3.39
1de3	GLU96	0.00	5.10	4.07	4.28	5.61	4.78	1.48	2.88	2.60	0.41	1.95
1de3	GLU115	0.94	4.90	4.07	4.28	4.49	3.48	3.69	3.94	3.35	4.17	3.60
1de3	GLU140	2.22	4.30	4.07	4.28	4.54	3.29	3.73	3.97	3.48	3.83	3.34
1de3	GLU144	1.90	4.30	4.07	4.28	4.58	3.78	3.86	4.05	3.57	4.23	3.88
1de3	ASP9	0.00	3.90	3.90	3.68	4.05	3.17	3.43	3.58	3.28	2.67	3.02
1de3	ASP57	16.29	4.30	3.90	3.68	3.98	3.26	3.60	3.70	3.49	4.31	3.59
1de3	ASP59	5.11	4.10	3.90	3.68	3.63	2.72	3.24	3.51	3.27	2.42	3.05
1de3	ASP75	0.91	3.90	3.90	3.68	3.59	2.58	2.88	3.35	3.03	2.53	2.22
1de3	ASP85	0.82	3.80	3.90	3.68	3.60	2.94	3.28	3.54	3.43	4.01	3.61
1de3	ASP109	12.43	3.70	3.90	3.68	3.92	3.12	3.40	3.60	3.44	2.98	3.14
1div	GLU17	4.71	3.57	4.07	4.28	4.51	3.22	3.55	3.85	3.23	3.74	3.19
1div	GLU38	0.00	4.04	4.07	4.28	4.23	3.85	4.16	4.15	3.67	4.30	3.89
1div	GLU48	1.21	4.21	4.07	4.28	4.49	3.87	3.89	4.06	3.50	4.23	3.98
1div	GLU54	12.06	4.21	4.07	4.28	4.23	3.82	3.59	3.87	2.98	3.98	3.34
1div	ASP8	8.90	2.99	3.90	3.68	3.90	3.03	3.37	3.58	3.56	3.75	3.03
1div	ASP23	16.07	3.05	3.90	3.68	2.88	3.43	3.46	3.63	3.70	3.31	3.21
1egf, 3egf, 1epi, 1eph, 1epg	GLU24	13.93	4.10	4.07	4.28	4.61	3.53	4.02	4.11	3.90	4.45	3.85

1egf, 3egf, 1epi, 1eph, 1epg	ASP11	24.27	3.90	3.90	3.68	3.91	3.86	3.81	3.81	4.00	4.49	3.93
1egf, 3egf, 1epi, 1eph, 1epg	ASP27	8.76	4.00	3.90	3.68	3.73	3.70	3.72	3.76	3.84	4.17	3.62
1egf, 3egf, 1epi, 1eph, 1epg	ASP40	11.57	3.60	3.90	3.68	3.67	3.18	3.38	3.57	3.60	4.29	3.43
1egf, 3egf, 1epi, 1eph, 1epg	ASP46	8.20	3.80	3.90	3.68	3.67	3.01	3.50	3.64	3.51	3.67	3.54
1era, 3ebx	HIS26	0.00	5.80	6.04	6.57	5.87	5.85	6.02	6.36	6.19	7.02	6.20
1ert, 1eru	HIS43	0.00	5.50	6.04	6.57	6.33	6.78	6.94	6.85	7.11	7.22	6.86
1ert, 1eru	GLU6	29.52	4.80	4.07	4.28	5.67	4.13	5.16	4.66	4.52	7.71	5.12
1ert, 1eru	GLU13	6.85	4.80	4.07	4.28	4.72	4.26	4.24	4.20	4.03	4.93	4.34
1ert, 1eru	GLU47	32.36	4.10	4.07	4.28	4.32	4.31	3.75	3.98	3.70	4.08	3.79
1ert, 1eru	GLU56	0.00	5.00	4.07	4.28	5.23	4.30	4.67	4.42	3.76	4.51	4.11
1ert, 1eru	GLU68	30.88	4.90	4.07	4.28	4.84	4.45	4.24	4.20	3.78	5.44	4.61
1ert, 1eru	GLU70	7.98	4.60	4.07	4.28	4.31	4.23	3.98	4.10	3.88	4.61	4.14
1ert, 1eru	GLU88	37.60	3.70	4.07	4.28	3.76	3.85	3.25	3.72	3.35	3.87	3.60
1ert, 1eru	GLU95	12.44	4.10	4.07	4.28	4.61	3.74	3.95	4.08	3.73	4.49	3.91
1ert, 1eru	GLU98	0.00	3.90	4.07	4.28	3.16	4.14	2.97	3.58	3.37	3.88	3.15
1ert, 1eru	GLU103	25.28	4.40	4.07	4.28	4.74	4.45	4.28	4.23	4.20	5.42	4.63
1ert, 1eru	ASP16	20.38	3.70	3.90	3.68	3.95	4.29	3.88	3.84	3.98	4.20	3.98
1ert, 1eru	ASP20	8.12	3.60	3.90	3.68	3.29	3.43	3.43	3.62	3.50	3.43	3.35
1ert, 1eru	ASP26	0.00	9.90	3.90	3.68	7.43	7.62	4.50	4.06	3.71	9.13	4.62
1ert, 1eru	ASP58	0.00	3.10	3.90	3.68	4.36	3.08	4.07	3.80	4.01	4.95	3.85
1ert, 1eru	ASP60	18.96	4.20	3.90	3.68	4.14	3.83	4.10	3.94	3.38	5.13	4.33
1ert, 1eru	ASP61	35.24	5.30	3.90	3.68	3.90	4.52	4.65	4.27	4.19	4.63	5.20
1ert, 1eru	ASP64	8.72	3.10	3.90	3.68	3.33	2.72	3.67	3.71	3.43	3.97	3.48
1ey0, 1stn	HIS8	7.40	6.52	6.04	6.57	6.34	6.40	6.74	6.71	6.59	7.53	6.47
1ey0, 1stn	HIS46	13.46	5.86	6.04	6.57	6.38	4.66	2.30	4.50	6.06	0.63	4.86
1ey0, 1stn	HIS121	0.60	5.30	6.04	6.57	6.28	5.44	6.88	6.74	6.69	7.03	6.61
1ey0, 1stn	HIS124	6.24	5.73	6.04	6.57	6.20	5.72	6.23	6.48	6.05	6.63	5.99
1fez	LYS53	2.18	9.30	10.54	10.51	8.45	9.64	12.30	11.30	13.70	11.04	12.35
1fmh	GLU6	2.11	4.82	4.07	4.28	4.66	4.39	4.12	4.17	3.80	4.45	3.95

1fmh	GLU8	0.22	4.52	4.07	4.28	3.90	4.09	3.15	3.66	3.31	3.57	2.93
1fmh	GLU13	0.92	4.37	4.07	4.28	3.86	4.41	3.39	3.81	3.57	3.84	3.67
1fmh	GLU15	0.04	4.11	4.07	4.28	3.31	4.38	2.62	3.43	2.84	3.25	2.91
1fmh	GLU20	2.87	4.41	4.07	4.28	3.78	4.37	3.21	3.72	3.40	4.57	3.94
1fmh	GLU22	0.52	4.82	4.07	4.28	4.09	4.47	3.63	3.91	3.65	3.96	3.96
1fmh	GLU27	4.05	4.65	4.07	4.28	3.85	4.24	3.33	3.76	3.34	4.63	3.52
1fmh	GLU29	27.61	4.63	4.07	4.28	4.64	4.22	4.23	4.23	3.95	5.37	4.54
1fna	ASP67	1.07	4.20	3.90	3.68	2.61	2.41	3.16	3.44	10.52	4.21	3.34
1goa	HIS62	34.56	7.00	6.04	6.57	6.64	6.73	6.84	6.82	6.48	7.62	6.78
1goa	HIS124	14.20	7.10	6.04	6.57	6.65	6.56	7.94	7.36	7.44	8.91	7.74
1goa	GLU6	0.00	4.50	4.07	4.28	4.28	3.25	3.56	3.90	3.10	3.09	2.45
1goa	GLU32	38.69	3.60	4.07	4.28	3.24	3.38	3.16	3.70	2.96	2.71	2.71
1goa	GLU48	2.14	4.40	4.07	4.28	6.07	4.07	3.57	3.92	2.78	3.56	3.37
1goa	GLU57	21.97	3.20	4.07	4.28	3.96	4.65	2.81	3.47	3.05	3.07	3.33
1goa	GLU61	6.04	3.90	4.07	4.28	4.40	3.80	3.80	4.00	3.47	4.34	3.39
1goa	GLU64	6.56	4.40	4.07	4.28	4.62	3.94	3.61	3.91	3.75	5.08	4.49
1goa	GLU119	2.42	4.10	4.07	4.28	4.48	3.52	3.66	3.93	3.35	4.11	3.52
1goa	GLU129	1.09	3.60	4.07	4.28	4.15	3.67	3.39	3.82	3.16	3.31	2.84
1goa	GLU131	1.07	4.30	4.07	4.28	4.57	3.90	4.21	4.20	3.70	4.46	3.79
1goa	GLU135	10.99	4.30	4.07	4.28	4.83	4.08	4.37	4.30	3.90	5.01	4.63
1goa	GLU147	17.42	4.20	4.07	4.28	4.57	3.91	4.24	4.22	4.01	5.11	4.45
1goa	GLU154	28.95	4.40	4.07	4.28	4.70	3.68	4.11	4.15	3.78	4.69	4.19
1goa	ASP10	2.42	6.10	3.90	3.68	7.61	4.56	4.22	4.07	3.45	5.64	4.65
1goa	ASP70	17.28	2.60	3.90	3.68	4.70	2.73	3.39	3.59	3.27	1.93	2.90
1goa	ASP94	26.26	3.20	3.90	3.68	2.88	2.68	3.13	3.45	3.04	3.26	2.72
1goa	ASP108	5.36	3.20	3.90	3.68	3.58	3.90	2.24	3.01	2.75	2.50	2.37
1goa	ASP134	0.00	4.10	3.90	3.68	3.91	3.61	3.03	3.42	3.28	2.65	2.55
1gs9	LYS69	3.62	10.40	10.54	10.51	10.62	10.60	10.76	10.62	11.09	10.59	10.94
1gs9	LYS72	4.29	10.00	10.54	10.51	10.09	10.68	10.36	10.42	11.03	9.72	10.71

1gs9	LYS75	0.00	10.10	10.54	10.51	11.27	10.62	11.85	11.17	12.10	10.34	12.00
1gs9	LYS95	5.50	10.10	10.54	10.51	10.44	10.68	10.39	10.42	10.82	10.28	10.48
1gs9	LYS143	9.78	9.50	10.54	10.51	10.37	10.13	10.03	10.24	10.44	9.20	9.88
1gs9	LYS146	7.92	9.20	10.54	10.51	10.27	10.20	10.08	10.27	10.21	9.28	9.62
1gs9	LYS157	5.50	11.10	10.54	10.51	10.59	10.83	10.84	10.64	10.95	10.52	10.78
1h4g	HIS11	0.60	6.52	6.04	6.57	5.70	5.67	4.14	5.45	6.34	0.00	5.16
1h4g	HIS32	7.89	6.68	6.04	6.57	6.25	6.62	6.42	6.57	6.50	7.29	6.40
1h4g	HIS60	0.00	4.01	6.04	6.57	5.78	5.33	5.99	6.31	6.79	6.12	6.21
1h4g	GLU17	0.00	4.31	4.07	4.28	4.43	3.33	2.27	3.09	3.13	3.09	3.17
1h4g	GLU56	17.68	4.55	4.07	4.28	3.91	4.11	3.26	3.71	3.09	3.84	3.44
1h4g	GLU94	0.00	3.94	4.07	4.28	5.92	5.42	3.67	4.00	3.41	5.56	4.57
1h4g	GLU126	24.64	4.51	4.07	4.28	4.47	3.24	3.19	3.70	3.20	3.65	3.43
1h4g	GLU167	20.50	3.58	4.07	4.28	3.88	4.32	2.60	3.35	2.93	3.26	3.09
1h4g	GLU178	0.00	4.10	4.07	4.28	2.62	5.49	0.24	2.35	1.80	0.00	0.18
1h4g	GLU184	0.00	6.50	4.07	4.28	7.20	5.06	4.04	4.10	3.72	5.14	4.24
1h4g	ASP5	2.77	3.84	3.90	3.68	3.60	3.73	3.65	3.71	3.74	3.80	3.83
1h4g	ASP12	30.35	3.94	3.90	3.68	2.74	3.26	2.71	3.25	3.08	3.33	2.36
1h4g	ASP15	0.00	3.35	3.90	3.68	3.41	3.52	2.58	3.17	2.96	2.79	2.51
1h4g	ASP21	11.88	3.46	3.90	3.68	3.55	3.05	2.83	3.28	2.94	3.79	3.47
1h4g	ASP90	30.42	3.88	3.90	3.68	3.90	3.67	3.50	3.65	3.64	4.10	3.50
1hic	GLU8	0.00	4.28	4.07	4.28	4.36	3.68	4.07	4.14	3.86	4.89	4.15
1hic	GLU17	1.79	3.79	4.07	4.28	4.30	3.94	4.09	4.13	3.74	4.87	4.17
1hic	GLU35	10.95	4.32	4.07	4.28	4.17	3.84	4.33	4.26	3.98	5.67	4.50
1hic	GLU43	12.68	4.24	4.07	4.28	4.33	3.81	3.93	4.05	3.87	4.84	4.22
1hic	ASP5	2.36	4.25	3.90	3.68	4.02	3.45	3.78	3.79	3.77	4.44	3.88
1hic	ASP33	25.01	4.24	3.90	3.68	3.79	3.64	3.82	3.81	3.95	3.96	3.82
1hng	GLU29	0.00	4.42	4.07	4.28	4.34	3.80	3.21	3.76	3.36	3.53	3.76
1hng	GLU33	1.14	4.16	4.07	4.28	4.72	3.74	3.93	4.07	3.62	4.38	3.70
1hng	GLU41	1.60	6.73	4.07	4.28	4.42	4.22	3.83	3.96	3.44	5.16	3.44

1hng	GLU56	0.00	3.92	4.07	4.28	4.31	3.57	3.61	3.92	3.25	4.15	3.69
1hng	GLU99	1.82	4.25	4.07	4.28	4.16	3.10	3.52	3.86	3.30	3.93	3.17
1hng	ASP25	18.94	3.53	3.90	3.68	3.64	3.13	3.49	3.65	3.67	3.94	3.50
1hng	ASP26	2.84	3.58	3.90	3.68	3.62	2.97	3.65	3.74	3.71	4.23	3.72
1hng	ASP28	0.00	3.57	3.90	3.68	2.38	3.20	2.18	2.96	2.75	2.66	2.02
1hng	ASP62	0.00	4.15	3.90	3.68	4.20	4.26	3.30	3.54	3.46	4.92	3.72
1hng	ASP71	19.43	3.18	3.90	3.68	3.89	3.32	3.55	3.67	3.64	3.89	3.67
1hng	ASP72	0.00	4.14	3.90	3.68	4.57	3.62	3.32	3.57	3.26	3.75	2.54
1hng	ASP94	0.60	3.87	3.90	3.68	3.82	3.03	3.29	3.55	3.46	3.41	3.12
1hv0	GLU78	0.00	4.60	4.07	4.28	6.18	6.97	3.49	3.89	3.24	3.84	3.54
1hv0	GLU172	0.00	7.20	4.07	4.28	8.26	5.48	4.58	4.22	3.62	6.42	4.58
1hv1	GLU78	0.00	4.60	4.07	4.28	7.57	6.10	5.00	4.43	3.70	5.31	5.02
1hv1	GLU172	1.07	7.20	4.07	4.28	6.36	5.32	4.12	4.07	3.76	5.44	4.33
1ig5	GLU4	19.69	3.77	4.07	4.28	4.21	3.62	4.03	4.12	3.52	4.70	4.09
1ig5	GLU5	12.61	3.40	4.07	4.28	2.97	3.78	3.66	3.93	3.51	3.70	3.63
1ig5	GLU11	6.43	4.74	4.07	4.28	4.52	4.36	4.09	4.14	3.96	4.88	4.37
1ig5	GLU17	7.11	3.62	4.07	4.28	5.88	4.24	5.25	4.68	4.12	5.38	4.85
1ig5	GLU26	3.35	4.08	4.07	4.28	4.54	3.61	4.09	4.16	3.74	4.41	3.87
1ig5	GLU48	3.62	4.62	4.07	4.28	4.36	3.93	4.50	4.34	3.90	5.19	4.88
1ig5	GLU64	5.50	3.84	4.07	4.28	4.06	3.41	3.99	4.09	3.14	4.03	4.23
1ig5	LYS7	0.00	11.36	10.54	10.51	10.58	10.98	11.29	10.88	11.49	11.14	11.44
1ig5	LYS12	16.09	11.06	10.54	10.51	11.75	10.65	11.80	11.12	11.87	10.59	12.00
1ig5	LYS16	32.28	11.06	10.54	10.51	10.52	11.12	11.01	10.73	11.45	10.83	11.35
1ig5	LYS25	2.19	11.81	10.54	10.51	11.40	10.86	11.25	10.84	11.78	12.12	12.00
1ig5	LYS29	2.28	11.28	10.54	10.51	10.33	10.72	10.79	10.62	11.12	10.40	10.82
1ig5	LYS41	24.92	10.93	10.54	10.51	10.39	10.73	10.46	10.45	10.85	10.07	10.48
1ig5	LYS55	39.12	12.12	10.54	10.51	10.80	11.25	11.76	11.11	2.99	11.72	12.00
1ig5	LYS71	8.18	10.73	10.54	10.51	10.19	10.53	10.34	10.39	10.98	9.89	10.31
1ig5	ASP47	5.50	3.04	3.90	3.68	2.64	3.12	2.70	3.27	2.92	2.75	2.61

1kf3, 9rat, 3rn3, 1rnz, 3srn	HIS105	0.00	6.50	6.04	6.57	6.89	5.13	6.86	6.83	6.85	8.16	6.75
1kf3, 9rat, 3rn3, 1rnz, 3srn	HIS119	2.66	6.50	6.04	6.57	6.46	5.66	6.18	6.48	7.09	6.70	6.22
1kf3, 9rat, 3rn3, 1rnz, 3srn	GLU2	33.98	2.70	4.07	4.28	2.75	3.49	1.99	3.05	2.57	1.81	2.16
1kf3, 9rat, 3rn3, 1rnz, 3srn	GLU9	4.58	4.00	4.07	4.28	5.04	4.08	4.02	4.11	3.90	4.70	3.75
1kf3, 9rat, 3rn3, 1rnz, 3srn	GLU49	4.83	4.30	4.07	4.28	4.64	3.95	4.35	4.29	4.22	5.13	3.91
1kf3, 9rat, 3rn3, 1rnz, 3srn	GLU86	24.86	4.00	4.07	4.28	4.10	4.01	4.28	4.19	4.18	4.60	3.49
1kf3, 9rat, 3rn3, 1rnz, 3srn	GLU111	5.44	3.50	4.07	4.28	4.70	3.73	3.79	4.00	3.82	4.14	3.83
1kf3, 9rat, 3rn3, 1rnz, 3srn	ASP38	26.73	2.10	3.90	3.68	3.68	3.04	3.31	3.55	3.45	3.37	2.86
1kf3, 9rat, 3rn3, 1rnz, 3srn	ASP53	3.75	3.70	3.90	3.68	3.95	4.36	3.83	3.81	4.04	3.79	3.74
1kf3, 9rat, 3rn3, 1rnz, 3srn	ASP83	0.24	3.30	3.90	3.68	4.53	3.19	2.46	3.14	3.39	2.70	2.35
1kf3, 9rat, 3rn3, 1rnz, 3srn	ASP121	18.22	3.00	3.90	3.68	2.83	3.69	1.49	2.67	3.14	1.36	1.74
1l54	LYS102	0.00	6.50	10.54	10.51	6.75	8.82	9.82	10.12	11.06	5.37	10.39
1l98	GLU105	31.21	6.00	4.07	4.28	5.70	4.32	2.64	3.55	3.22	5.93	4.81
1lni, 1rgg	HIS53	0.00	8.27	6.04	6.57	5.91	7.34	7.26	7.03	7.18	9.03	7.81
1lni, 1rgg	HIS85	13.93	6.35	6.04	6.57	5.97	6.42	6.07	6.39	6.18	6.91	6.09
1lni, 1rgg	GLU14	0.00	5.02	4.07	4.28	4.32	3.76	4.05	4.12	3.47	5.85	4.37
1lni, 1rgg	GLU41	28.86	4.14	4.07	4.28	4.58	4.10	3.99	4.10	3.64	4.25	4.10
1lni, 1rgg	GLU74	33.83	3.47	4.07	4.28	4.69	4.07	4.36	4.30	4.05	5.50	4.78
1lni, 1rgg	GLU78	2.39	3.13	4.07	4.28	3.46	3.74	4.13	4.13	3.97	4.68	3.66
1lni, 1rgg	ASP17	2.85	3.72	3.90	3.68	4.11	4.24	3.87	3.84	3.88	4.18	3.92
1lni, 1rgg	ASP25	20.10	4.87	3.90	3.68	3.99	4.18	4.00	3.91	4.20	4.97	4.39
1lni, 1rgg	ASP33	15.57	2.39	3.90	3.68	3.49	4.31	2.50	3.12	2.82	1.09	2.39
1lni, 1rgg	ASP79	0.00	7.37	3.90	3.68	5.67	4.77	4.55	4.15	4.11	6.39	5.40
1lni, 1rgg	ASP84	16.39	3.01	3.90	3.68	1.77	2.87	3.10	3.43	3.39	3.23	2.88
1lni, 1rgg	ASP93	3.62	3.09	3.90	3.68	2.73	3.40	3.54	3.63	3.99	3.89	3.78
1lse, 6lyz, 4lzt, 2lzt, 1lys	GLU7	14.57	2.85	4.07	4.28	3.38	4.06	2.98	3.61	3.21	3.27	2.96
1lse, 6lyz, 4lzt, 2lzt, 1lys	GLU35	18.97	6.20	4.07	4.28	6.65	4.75	4.25	4.27	3.63	5.01	4.08
1lse, 6lyz, 4lzt, 2lzt, 1lys	LYS13	13.39	10.50	10.54	10.51	11.08	10.49	11.09	10.76	10.74	10.85	10.70
1lse, 6lyz, 4lzt, 2lzt, 1lys	LYS33	4.82	10.60	10.54	10.51	10.11	10.26	10.01	10.24	11.01	9.87	10.40

1lse, 6lyz, 4lzt, 2lzt, 1lys	LYS96	0.94	10.80	10.54	10.51	10.07	10.40	11.01	10.73	11.52	10.64	11.25
1lse, 6lyz, 4lzt, 2lzt, 1lys	LYS97	8.35	10.30	10.54	10.51	10.40	10.69	11.00	10.72	11.49	10.33	10.78
1lse, 6lyz, 4lzt, 2lzt, 1lys	LYS116	21.32	10.40	10.54	10.51	10.19	10.57	10.10	10.27	10.83	9.67	10.20
1lse, 6lyz, 4lzt, 2lzt, 1lys	ASP18	18.79	2.66	3.90	3.68	3.52	2.85	2.97	3.35	3.02	2.97	2.75
1lse, 6lyz, 4lzt, 2lzt, 1lys	ASP48	29.24	1.60	3.90	3.68	2.49	2.91	2.61	3.20	3.39	2.92	2.85
1lse, 6lyz, 4lzt, 2lzt, 1lys	ASP52	0.00	3.68	3.90	3.68	4.40	3.72	2.91	3.34	3.46	4.54	4.07
1lse, 6lyz, 4lzt, 2lzt, 1lys	ASP66	31.43	0.90	3.90	3.68	2.19	2.21	2.22	2.96	2.68	2.48	2.23
1lse, 6lyz, 4lzt, 2lzt, 1lys	ASP87	10.32	2.07	3.90	3.68	2.82	3.03	2.80	3.28	2.89	2.67	2.28
1lse, 6lyz, 4lzt, 2lzt, 1lys	ASP101	35.53	4.09	3.90	3.68	4.23	4.01	3.69	3.70	3.71	6.22	4.04
1lse, 6lyz, 4lzt, 2lzt, 1lys	ASP119	15.51	3.20	3.90	3.68	3.50	2.87	3.12	3.44	3.34	3.64	3.25
1lz1	HIS78	32.28	7.12	6.04	6.57	6.26	6.80	6.65	6.74	6.67	7.73	6.62
1mut	LYS39	2.51	8.40	10.54	10.51	10.40	10.82	10.67	10.55	11.12	10.21	10.73
1nzp	LYS312	0.14	9.58	10.54	10.51	10.92	10.43	11.29	10.84	11.66	9.23	11.65
1pga, 3gb1, 2gb1, 1pgb, 1gb1	GLU19	17.03	3.70	4.07	4.28	4.39	3.71	4.14	4.16	4.04	4.41	4.13
1pga, 3gb1, 2gb1, 1pgb, 1gb2	GLU27	0.62	4.50	4.07	4.28	4.67	3.76	4.12	4.15	3.77	4.28	4.19
1pga, 3gb1, 2gb1, 1pgb, 1gb3	GLU42	0.58	4.40	4.07	4.28	4.81	3.69	4.55	4.37	4.02	5.23	4.60
1pga, 3gb1, 2gb1, 1pgb, 1gb4	GLU56	20.98	4.00	4.07	4.28	5.10	3.85	4.31	4.24	3.73	4.50	3.33
1pga, 3gb1, 2gb1, 1pgb, 1gb5	LYS28	2.08	10.90	10.54	10.51	10.44	10.83	10.58	10.51	11.03	10.70	10.74
1pga, 3gb1, 2gb1, 1pgb, 1gb6	ASP22	0.03	2.90	3.90	3.68	3.15	2.01	2.90	3.34	3.20	3.12	3.05
1pga, 3gb1, 2gb1, 1pgb, 1gb7	ASP36	28.68	3.80	3.90	3.68	4.03	4.26	3.97	3.89	4.07	4.95	4.41
1pga, 3gb1, 2gb1, 1pgb, 1gb8	ASP40	12.85	4.00	3.90	3.68	3.99	3.56	3.91	3.85	3.86	4.94	4.10
1pga, 3gb1, 2gb1, 1pgb, 1gb9	ASP46	0.04	3.60	3.90	3.68	3.36	2.89	3.37	3.56	3.65	3.55	3.50
1pga, 3gb1, 2gb1, 1pgb, 1gb10	ASP47	8.38	3.40	3.90	3.68	3.87	3.34	3.52	3.66	3.72	3.92	3.51
1pnt, 1z12	HIS66	24.79	8.29	6.04	6.57	6.66	7.51	6.94	6.88	7.15	8.20	7.41
1pnt, 1z12	HIS72	10.22	9.19	6.04	6.57	5.79	5.97	7.31	7.05	7.06	8.57	7.45
1poh	HIS76	5.50	6.00	6.04	6.57	5.81	6.54	5.95	6.35	6.28	6.48	6.39
1ptd	HIS92	0.00	5.40	6.04	6.57	5.76	6.77	6.35	6.53	7.84	7.04	6.25
1sap	GLU11	4.71	4.19	4.07	4.28	4.34	3.66	4.02	4.10	3.63	4.43	3.96
1sap	GLU12	4.30	4.41	4.07	4.28	4.80	3.75	4.28	4.20	3.75	5.65	4.48

1sap	GLU14	11.79	4.00	4.07	4.28	3.65	3.30	3.37	3.78	3.01	3.38	2.98
1sap	GLU47	6.43	4.21	4.07	4.28	3.82	3.56	3.80	4.00	3.17	3.96	3.77
1sap	GLU53	0.00	3.53	4.07	4.28	5.53	4.16	4.10	4.18	3.81	5.49	4.62
1sap	GLU62	0.00	3.99	4.07	4.28	4.32	2.92	2.80	3.41	2.96	3.76	2.67
1sap	GLU64	19.30	4.23	4.07	4.28	4.48	3.84	3.87	4.04	3.56	4.52	4.09
1sap	ASP16	1.09	2.89	3.90	3.68	2.34	2.70	3.05	3.40	3.09	2.44	2.22
1sap	ASP35	0.00	3.42	3.90	3.68	3.32	3.18	2.98	3.34	2.75	3.91	3.47
1sap	ASP36	0.00	3.12	3.90	3.68	2.33	3.13	2.37	3.07	2.75	1.50	1.62
1sap	ASP49	8.57	3.55	3.90	3.68	2.94	3.34	2.65	3.19	2.80	3.39	2.80
1sap	ASP56	0.00	3.35	3.90	3.68	3.31	3.49	2.93	3.36	3.12	2.69	2.62
1trs, 1trw	GLU6	5.90	4.90	4.07	4.28	4.78	4.15	4.52	4.33	4.09	4.96	4.58
1trs, 1trw	GLU13	1.07	4.40	4.07	4.28	4.61	4.23	4.13	4.15	3.83	4.80	4.22
1trs, 1trw	GLU47	23.71	4.30	4.07	4.28	4.49	4.07	3.96	4.09	3.79	4.25	3.83
1trs, 1trw	GLU56	0.00	3.20	4.07	4.28	4.51	4.01	3.25	3.77	2.96	3.11	2.75
1trs, 1trw	GLU68	11.26	5.10	4.07	4.28	4.37	4.14	3.83	4.02	3.81	4.87	4.06
1trs, 1trw	GLU70	13.40	4.80	4.07	4.28	4.65	4.32	4.14	4.18	4.04	4.91	4.50
1trs, 1trw	GLU88	19.30	3.60	4.07	4.28	3.83	3.82	3.39	3.80	3.58	4.38	3.67
1trs, 1trw	GLU95	5.43	4.10	4.07	4.28	4.59	3.34	3.91	4.06	3.76	4.47	3.88
1trs, 1trw	GLU98	0.00	3.90	4.07	4.28	4.52	3.99	3.96	4.09	3.70	4.43	3.93
1trs, 1trw	GLU103	23.04	4.50	4.07	4.28	4.63	4.40	4.29	4.24	3.90	4.76	4.52
1trs, 1trw	ASP16	10.72	4.20	3.90	3.68	3.95	4.33	3.85	3.83	4.03	4.20	3.96
1trs, 1trw	ASP20	5.44	3.80	3.90	3.68	3.56	2.44	3.44	3.59	3.54	3.94	3.23
1trs, 1trw	ASP26	0.00	8.10	3.90	3.68	6.62	6.41	3.91	3.82	3.68	6.09	3.92
1trs, 1trw	ASP58	0.00	2.70	3.90	3.68	3.87	2.99	3.25	3.45	2.80	2.31	2.05
1trs, 1trw	ASP60	14.46	3.90	3.90	3.68	4.49	3.92	3.58	3.63	3.87	5.08	4.34
1trs, 1trw	ASP61	14.46	5.20	3.90	3.68	4.22	4.25	4.30	4.05	4.30	5.11	4.58
1trs, 1trw	ASP64	6.98	3.20	3.90	3.68	3.48	2.52	3.48	3.62	3.33	3.08	3.55
1u2u	HIS28	21.84	6.79	6.04	6.57	6.26	6.97	7.07	6.89	6.30	6.99	6.45
1u2u	GLU6	7.10	4.90	4.07	4.28	4.55	3.89	4.00	4.10	3.66	3.93	3.71

1u2u	GLU8	0.12	4.89	4.07	4.28	4.73	4.12	4.12	4.16	3.58	4.09	3.62
1u2u	GLU13	7.80	4.42	4.07	4.28	4.64	4.34	4.03	4.11	3.74	4.60	4.09
1u2u	GLU15	0.50	4.09	4.07	4.28	4.30	4.15	3.59	3.91	3.44	3.85	3.49
1u2u	GLU20	3.97	4.21	4.07	4.28	4.63	4.37	4.07	4.14	3.87	4.99	4.16
1u2u	GLU22	0.20	4.83	4.07	4.28	4.65	4.44	4.11	4.16	3.82	5.06	4.29
1u2u	GLU27	8.02	4.74	4.07	4.28	4.44	4.14	3.94	4.07	3.68	4.46	4.00
1u2u	GLU29	22.90	4.63	4.07	4.28	4.55	3.93	4.21	4.21	3.87	4.74	4.41
1ubq	GLU16	9.89	3.90	4.07	4.28	4.38	3.88	4.00	4.10	3.89	4.27	3.77
1ubq	GLU18	0.00	4.30	4.07	4.28	4.48	4.16	4.09	4.12	3.96	4.15	3.78
1ubq	GLU24	8.71	4.30	4.07	4.28	4.78	4.40	4.24	4.24	3.96	5.04	4.40
1ubq	GLU34	36.32	4.50	4.07	4.28	3.99	3.36	3.39	3.81	3.28	3.90	3.19
1ubq	GLU51	4.83	3.80	4.07	4.28	4.56	3.55	4.02	4.09	3.59	3.92	3.92
1ubq	GLU64	22.64	4.50	4.07	4.28	4.48	3.73	3.86	4.03	3.52	4.71	3.61
1ubq	ASP21	0.00	3.10	3.90	3.68	3.36	3.57	3.14	3.51	3.34	3.70	2.65
1ubq	ASP32	32.44	3.80	3.90	3.68	3.92	3.76	3.71	3.78	3.96	4.20	3.76
1ubq	ASP39	11.58	3.60	3.90	3.68	3.67	3.18	3.29	3.51	3.29	3.44	3.31
1ubq	ASP52	6.49	3.40	3.90	3.68	3.10	2.47	2.72	3.24	3.05	2.64	2.32
1ubq	ASP58	40.85	3.60	3.90	3.68	2.99	3.16	3.49	3.66	3.26	3.03	3.61
1ypi	HIS103	17.14	7.29	6.04	6.57	6.65	7.19	6.98	6.88	6.79	8.21	7.17
2bca	GLU26	0.00	4.08	4.07	4.28	5.22	4.23	4.65	4.39	4.11	5.32	4.43
2bca	LYS7	0.00	11.36	10.54	10.51	10.46	10.95	10.86	10.64	11.26	10.87	11.07
2bca	LYS12	2.14	11.06	10.54	10.51	10.55	10.66	11.12	10.74	11.16	9.92	10.78
2bca	LYS16	26.79	11.06	10.54	10.51	10.23	11.15	11.34	10.91	11.89	11.84	12.00
2bca	LYS29	0.00	11.28	10.54	10.51	10.12	10.27	10.32	10.40	10.98	9.74	10.39
2bca	LYS41	0.00	10.93	10.54	10.51	10.28	10.33	10.61	10.48	10.97	9.92	10.63
2bca	LYS55	28.93	12.12	10.54	10.51	10.55	11.20	11.05	10.75	11.42	11.27	11.40
2bca	LYS71	0.00	10.73	10.54	10.51	10.30	10.77	10.34	10.41	10.82	10.16	10.37
2bca	LYS72	9.64	11.33	10.54	10.51	11.27	10.99	11.76	11.08	11.96	11.73	12.00
2ci2	GLU23	5.36	3.20	4.07	4.28	4.54	3.41	3.85	4.01	3.43	4.51	3.78

2ci2	GLU26	24.96	3.30	4.07	4.28	3.30	3.54	3.05	3.62	3.00	3.42	3.38
2ci2	GLU33	3.63	4.20	4.07	4.28	4.74	3.20	4.14	4.18	3.80	4.00	4.21
2ci2	GLU34	8.18	3.80	4.07	4.28	3.84	3.76	4.01	4.06	3.62	4.14	4.05
2ci2	GLU45	14.94	3.90	4.07	4.28	3.27	3.88	3.37	3.77	3.39	3.58	3.75
2ci2	GLU60	11.55	3.50	4.07	4.28	3.69	3.38	3.39	3.78	3.27	3.88	3.32
2ci2	ASP42	32.42	3.30	3.90	3.68	2.39	3.88	2.46	3.07	3.47	4.06	2.90
2ci2	ASP64	1.21	3.80	3.90	3.68	2.90	2.85	2.59	3.21	2.64	2.57	2.37
2ci2	ASP71	13.67	2.80	3.90	3.68	2.74	2.85	2.43	3.05	3.23	3.58	3.14
2ci2	ASP74	0.00	5.00	3.90	3.68	4.13	4.28	4.07	3.96	4.02	4.99	4.49
2cpl	HIS70	16.21	5.84	6.04	6.57	6.15	6.70	6.36	6.56	6.34	6.83	6.14
2igh	GLU20	23.52	4.30	4.07	4.28	4.39	3.96	3.93	4.06	3.74	4.55	4.11
2igh	GLU29	1.27	4.20	4.07	4.28	4.70	3.76	4.10	4.15	3.81	4.70	4.28
2igh	GLU32	0.00	4.60	4.07	4.28	4.67	4.17	3.97	4.09	3.83	7.73	4.16
2igh	GLU61	29.75	4.20	4.07	4.28	4.79	4.09	4.32	4.25	3.79	5.31	4.03
2igh	ASP27	2.55	2.90	3.90	3.68	3.19	2.83	3.54	3.65	3.51	3.31	3.32
2igh	ASP41	22.51	3.90	3.90	3.68	3.95	4.26	3.80	3.80	4.01	4.31	4.11
2igh	ASP45	20.08	4.40	3.90	3.68	3.85	3.50	3.65	3.71	3.79	5.27	4.13
2igh	ASP51	1.70	3.60	3.90	3.68	2.52	3.06	2.86	3.32	3.19	3.84	3.03
2igh	ASP52	18.20	3.40	3.90	3.68	3.54	3.40	3.62	3.71	3.82	4.35	3.66
2lzm	ASP70	6.57	0.50	3.90	3.68	3.99	3.73	2.67	3.19	2.99	3.17	2.72
2ovo	GLU10	21.32	4.14	4.07	4.28	4.39	3.73	3.99	4.12	3.78	4.54	3.96
2ovo	GLU19	15.08	3.21	4.07	4.28	4.00	3.45	3.50	3.81	3.19	3.70	3.17
2ovo	GLU43	42.75	4.81	4.07	4.28	4.66	4.35	4.20	4.22	3.99	5.01	4.42
2ovo	LYS13	14.21	9.86	10.54	10.51	10.71	10.99	11.57	11.00	11.93	10.85	11.25
2ovo	LYS29	2.42	11.12	10.54	10.51	10.51	10.76	11.09	10.75	11.39	10.65	10.88
2ovo	LYS34	3.28	10.13	10.54	10.51	10.55	10.47	11.13	10.87	11.51	10.49	11.09
2ovo	LYS55	12.90	11.10	10.54	10.51	11.33	10.49	11.30	10.90	11.40	10.38	11.27
2ovo	ASP7	14.47	2.67	3.90	3.68	2.58	2.89	3.34	3.57	3.36	3.40	2.57
2ovo	ASP27	33.10	2.30	3.90	3.68	2.61	3.30	3.13	3.43	3.50	3.53	3.24

2qmt	GLU15	36.56	4.40	4.07	4.28	3.83	4.09	3.53	3.85	3.51	3.99	3.58
2qmt	GLU19	32.35	3.70	4.07	4.28	4.46	3.81	4.06	4.11	3.99	4.71	4.07
2qmt	GLU27	0.00	4.50	4.07	4.28	3.50	3.94	2.95	3.60	3.50	3.83	3.52
2qmt	GLU42	5.51	4.40	4.07	4.28	4.77	4.00	4.46	4.32	4.00	5.40	4.54
2qmt	GLU56	33.63	4.00	4.07	4.28	2.91	3.28	3.01	3.55	3.19	3.41	2.27
2qmt	LYS28	4.69	10.90	10.54	10.51	10.53	10.76	10.78	10.60	11.17	10.44	10.82
2qmt	ASP22	1.21	2.90	3.90	3.68	2.53	2.07	3.04	3.40	3.02	3.45	2.83
2qmt	ASP36	41.26	3.80	3.90	3.68	3.50	4.19	3.67	3.71	3.85	4.96	4.06
2qmt	ASP40	19.56	4.00	3.90	3.68	3.96	3.64	3.80	3.81	4.00	4.45	4.14
2qmt	ASP46	0.00	3.60	3.90	3.68	3.38	2.92	3.30	3.52	3.56	3.47	3.71
2qmt	ASP47	8.78	3.40	3.90	3.68	2.30	3.37	2.92	3.33	3.11	3.03	2.68
2rks	GLU67	30.00	3.90	4.07	4.28	4.53	4.21	3.65	3.92	3.41	4.32	3.78
2rks	GLU129	0.00	3.80	4.07	4.28	4.89	3.58	3.13	3.68	3.06	3.60	3.19
2rn2	HIS124	40.74	7.10	6.04	6.57	6.30	6.14	6.52	6.63	6.29	6.71	6.26
2rn2	HIS127	2.42	7.90	6.04	6.57	7.26	7.18	7.72	7.23	7.49	8.71	7.90
2rn2	GLU6	0.00	4.50	4.07	4.28	4.57	4.71	3.33	3.79	3.46	4.18	3.71
2rn2	GLU32	31.14	3.60	4.07	4.28	3.53	3.32	2.86	3.53	2.95	3.02	2.80
2rn2	GLU48	3.35	4.40	4.07	4.28	6.32	4.42	2.44	3.40	2.86	4.75	4.84
2rn2	GLU57	27.47	3.20	4.07	4.28	3.47	4.52	2.47	3.35	2.77	3.06	3.41
2rn2	GLU61	9.67	3.90	4.07	4.28	3.77	3.72	3.40	3.79	3.07	2.91	2.75
2rn2	GLU64	5.42	4.40	4.07	4.28	4.60	3.33	3.82	4.03	3.73	4.46	3.74
2rn2	GLU119	2.42	4.10	4.07	4.28	3.62	3.85	3.36	3.79	3.02	4.61	3.26
2rn2	GLU129	1.21	3.60	4.07	4.28	3.28	4.18	2.73	3.39	3.10	2.25	2.66
2rn2	GLU131	8.99	4.30	4.07	4.28	4.76	4.16	4.46	4.35	4.03	5.09	4.49
2rn2	GLU135	2.14	4.30	4.07	4.28	4.53	4.15	4.13	4.17	3.80	4.77	4.33
2rn2	GLU147	23.70	4.20	4.07	4.28	4.66	3.86	4.13	4.17	3.95	4.86	4.30
2rn2	GLU154	29.35	4.40	4.07	4.28	4.21	3.22	4.04	4.11	3.53	3.93	4.09
2rn2	ASP10	3.63	6.10	3.90	3.68	7.09	4.55	5.19	4.44	3.89	6.61	4.79
2rn2	ASP70	24.78	2.60	3.90	3.68	4.86	3.22	4.17	3.98	3.70	3.29	3.46

2rn2	ASP94	29.91	3.20	3.90	3.68	2.78	2.36	3.26	3.51	3.33	3.44	3.04
2rn2	ASP108	4.29	3.20	3.90	3.68	3.95	4.20	2.74	3.26	3.03	3.05	2.85
2rn2	ASP134	0.00	4.30	3.90	3.68	3.58	4.29	3.44	3.52	3.47	2.71	3.34
2trx	HIS6	24.34	6.20	6.04	6.57	5.98	6.83	6.61	6.70	6.58	7.88	6.58
2trx	ASP20	20.58	3.80	3.90	3.68	3.85	3.23	3.69	3.74	3.91	4.26	4.09
2trx	ASP26	0.55	7.50	3.90	3.68	6.62	6.27	3.38	3.53	3.34	5.72	3.57
2zta	HIS18	4.71	6.24	6.04	6.57	6.24	6.20	6.71	6.75	6.29	7.44	6.60
2zta	GLU6	3.35	4.60	4.07	4.28	4.84	4.25	4.27	4.25	3.75	4.97	3.90
2zta	GLU10	15.55	3.94	4.07	4.28	5.14	4.44	4.72	4.44	4.16	5.63	4.87
2zta	GLU11	14.33	4.05	4.07	4.28	4.72	3.68	3.33	3.76	3.71	4.67	3.19
2zta	GLU20	6.57	4.38	4.07	4.28	4.68	4.27	3.35	3.79	3.60	4.54	3.87
2zta	GLU22	0.00	4.20	4.07	4.28	3.83	3.74	2.60	3.39	2.94	3.82	2.44
2zta	LYS3	3.37	10.78	10.54	10.51	10.44	10.65	10.10	10.28	10.78	10.30	9.95
2zta	LYS8	2.30	11.26	10.54	10.51	10.51	10.77	11.14	10.79	11.35	10.72	11.44
2zta	LYS15	7.13	10.62	10.54	10.51	10.86	10.73	11.20	10.82	11.44	11.38	11.34
2zta	LYS27	6.57	11.10	10.54	10.51	10.42	10.59	11.01	10.71	10.86	9.45	10.99
2zta	LYS28	25.48	10.64	10.54	10.51	10.43	10.69	10.35	10.40	10.70	10.20	10.42
2zta	ASP7	1.07	3.48	3.90	3.68	4.09	4.03	4.18	3.98	3.80	4.39	3.96
3ssi	HIS43	9.64	3.25	6.04	6.57	6.04	1.80	4.18	5.35	6.75	4.58	6.37
9rnt, 1ygw	HIS27	10.67	7.57	6.04	6.57	5.61	7.44	6.90	6.78	6.91	8.18	7.12
9rnt, 1ygw	HIS40	8.50	7.44	6.04	6.57	6.44	6.83	6.89	6.77	7.02	10.11	7.37
9rnt, 1ygw	HIS92	13.39	7.31	6.04	6.57	5.08	7.80	5.19	5.89	6.16	6.87	5.75
9rnt, 1ygw	GLU28	42.65	5.61	4.07	4.28	4.99	4.13	4.41	4.29	3.96	4.88	4.78
9rnt, 1ygw	GLU31	4.90	5.36	4.07	4.28	4.66	4.53	4.52	4.36	4.16	5.12	4.59
9rnt, 1ygw	GLU46	45.83	3.62	4.07	4.28	4.67	4.22	4.16	4.17	3.85	5.94	4.21
9rnt, 1ygw	GLU58	0.17	3.96	4.07	4.28	7.30	3.67	1.60	2.87	3.03	2.64	2.89
9rnt, 1ygw	GLU82	12.70	3.27	4.07	4.28	4.14	4.01	3.70	3.94	3.69	3.55	3.50
9rnt, 1ygw	GLU102	14.79	5.30	4.07	4.28	5.60	4.89	5.00	4.61	4.16	6.23	5.26
9rnt, 1ygw	ASP3	24.53	3.54	3.90	3.68	3.37	3.37	3.74	3.77	3.75	3.54	3.36

9rnt, 1ygw	ASP15	4.49	3.52	3.90	3.68	4.20	2.95	4.18	3.94	3.88	4.61	3.93
9rnt, 1ygw	ASP29	34.93	4.26	3.90	3.68	3.68	3.94	3.96	3.87	3.78	4.61	4.03
9rnt, 1ygw	ASP49	28.80	4.22	3.90	3.68	3.69	3.82	4.26	4.00	4.15	4.63	4.28
9rnt, 1ygw	ASP66	6.92	3.90	3.90	3.68	4.02	3.56	3.93	3.88	3.90	4.51	3.86
9rnt, 1ygw	ASP76	0.46	0.50	3.90	3.68	5.07	3.49	4.22	3.90	3.55	4.10	3.58

Table S2. Experimental and predicted p*K*a values from the Small Set (n=112).

PDB ID	Residue	Expt p*Ka*	Soln p*Ka*	Avg p*Ka*	Propka3	DeepKa	cpHMD	PKAI	PKAI+	DelPhiPKa	MCCE2	H++
135l	GLU7	2.68	4.07	4.28	2.94	3.32	3.32	2.74	3.51	2.85	3.04	2.95
135l	GLU35	6.06	4.07	4.28	6.78	4.43	5.40	4.69	4.48	3.65	5.22	4.23
135l	ASP18	2.68	3.90	3.68	2.94	2.79	1.40	1.94	2.85	2.79	2.06	2.28
1a2p, 1fw7, 1bnr, 1bnj 1bni	HIS18	7.75	6.04	6.57	6.23	7.00	8.01	6.90	6.84	6.73	6.21	6.92
1a2p, 1fw7, 1bnr, 1bnj 1bni	GLU29	3.75	4.07	4.28	3.97	3.76	3.65	4.17	4.18	3.96	4.16	4.21
1a2p, 1fw7, 1bnr, 1bnj 1bni	GLU60	3.00	4.07	4.28	4.26	3.62	3.24	3.79	4.00	4.19	3.87	3.70
1a2p, 1fw7, 1bnr, 1bnj 1bni	GLU73	2.10	4.07	4.28	5.24	3.59	5.04	2.27	3.26	2.96	2.30	1.37
1a2p, 1fw7, 1bnr, 1bnj 1bni	ASP12	3.80	3.90	3.68	3.82	3.99	4.02	3.61	3.70	3.66	4.16	3.96
1a91	GLU2	5.50	4.07	4.28	4.59	3.70	4.00	4.20	4.19	4.03	4.92	4.06
1a91	GLU37	5.50	4.07	4.28	4.57	4.10	4.11	4.00	4.10	3.78	4.79	4.10
1a91	ASP7	5.60	3.90	3.68	4.04	4.42	4.45	3.81	3.81	3.96	4.66	4.01
1a91	ASP44	5.60	3.90	3.68	3.98	3.47	3.77	4.01	3.92	3.97	4.57	4.32
1a91	ASP61	7.10	3.90	3.68	4.14	4.63	4.58	4.12	3.96	3.99	4.88	4.33
1ans	GLU20	5.40	4.07	4.28	4.59	4.12	4.34	4.25	4.22	4.04	5.60	4.39
1bhc, 4pti, 1d0d, 1bpi	GLU49	3.80	4.07	4.28	3.52	3.74	3.62	2.72	3.42	3.61	2.95	2.05
1bhc, 4pti, 1d0d, 1bpi	LYS26	10.60	10.54	10.51	10.43	10.66	10.52	10.45	10.46	10.94	10.11	10.49
1bhc, 4pti, 1d0d, 1bpi	LYS46	10.60	10.54	10.51	10.02	10.57	10.14	10.29	10.38	10.84	8.95	10.00
1cdc	GLU29	4.42	4.07	4.28	-0.75	3.31	3.58	-0.59	1.93	1.44	0.00	0.00
1cdc	GLU41	6.73	4.07	4.28	6.36	3.94	3.79	0.96	2.60	2.63	0.00	3.52
1cdc	ASP62	4.15	3.90	3.68	4.61	3.84	3.69	2.43	3.13	3.65	4.36	4.28
1cdc	ASP71	3.18	3.90	3.68	3.75	3.90	3.02	3.40	3.59	3.73	4.24	3.44
1cdc	ASP94	3.87	3.90	3.68	3.25	3.60	3.06	2.46	3.00	3.47	3.16	2.99
1de3	HIS35	6.30	6.04	6.57	5.88	7.19	6.47	6.83	6.81	6.65	7.59	6.51
1de3	HIS36	6.80	6.04	6.57	6.49	6.93	7.00	7.01	6.90	6.80	7.77	6.99
1de3	HIS50	7.70	6.04	6.57	5.35	6.70	7.58	6.26	6.49	6.64	7.89	6.69
1de3	HIS150	7.60	6.04	6.57	6.40	6.25	6.10	6.59	6.65	6.37	7.34	6.02
1de3	GLU19	4.60	4.07	4.28	4.45	3.59	3.39	3.70	3.95	3.41	4.39	3.73

1de3	GLU96	5.10	4.07	4.28	5.61	4.78	4.09	1.48	2.88	2.60	0.41	1.95
1ert, 1eru	ASP16	3.70	3.90	3.68	3.95	4.29	4.04	3.88	3.84	3.98	4.20	3.98
1ert, 1eru	ASP26	9.90	3.90	3.68	7.43	7.62	7.96	4.50	4.06	3.71	9.13	4.62
1ert, 1eru	ASP60	4.20	3.90	3.68	4.14	3.83	2.52	4.10	3.94	3.38	5.13	4.33
1ert, 1eru	ASP61	5.30	3.90	3.68	3.90	4.52	4.08	4.65	4.27	4.19	4.63	5.20
1gs9	LYS75	10.10	10.54	10.51	11.27	10.62	9.43	11.85	11.17	12.10	10.34	12.00
1gs9	LYS143	9.50	10.54	10.51	10.37	10.13	10.02	10.03	10.24	10.44	9.20	9.88
1gs9	LYS146	9.20	10.54	10.51	10.27	10.20	9.91	10.08	10.27	10.21	9.28	9.62
1h4g	HIS60	4.01	6.04	6.57	5.78	5.33	4.10	5.99	6.31	6.79	6.12	6.21
1h4g	GLU56	4.55	4.07	4.28	3.91	4.11	3.84	3.26	3.71	3.09	3.84	3.44
1h4g	GLU94	3.94	4.07	4.28	5.92	5.42	5.39	3.67	4.00	3.41	5.56	4.57
1h4g	GLU126	4.51	4.07	4.28	4.47	3.24	3.01	3.19	3.70	3.20	3.65	3.43
1h4g	GLU178	4.10	4.07	4.28	2.62	5.49	3.22	0.24	2.35	1.80	0.00	0.18
1h4g	GLU184	6.50	4.07	4.28	7.20	5.06	5.90	4.04	4.10	3.72	5.14	4.24
1h4g	ASP21	3.46	3.90	3.68	3.55	3.05	2.73	2.83	3.28	2.94	3.79	3.47
1hng	GLU41	6.73	4.07	4.28	4.42	4.22	4.16	3.83	3.96	3.44	5.16	3.44
1hng	GLU99	4.25	4.07	4.28	4.16	3.10	3.68	3.52	3.86	3.30	3.93	3.17
1hng	ASP25	3.53	3.90	3.68	3.64	3.13	3.35	3.49	3.65	3.67	3.94	3.50
1hv0	GLU172	7.20	4.07	4.28	8.26	5.48	7.60	4.58	4.22	3.62	6.42	4.58
1hv1	GLU78	4.60	4.07	4.28	7.57	6.10	6.58	5.00	4.43	3.70	5.31	5.02
1hv1	GLU172	7.20	4.07	4.28	6.36	5.32	7.15	4.12	4.07	3.76	5.44	4.33
1ig5	GLU5	3.40	4.07	4.28	2.97	3.78	3.77	3.66	3.93	3.51	3.70	3.63
1ig5	GLU11	4.74	4.07	4.28	4.52	4.36	4.73	4.09	4.14	3.96	4.88	4.37
1ig5	LYS7	11.36	10.54	10.51	10.58	10.98	11.37	11.29	10.88	11.49	11.14	11.44
1ig5	LYS12	11.06	10.54	10.51	11.75	10.65	11.30	11.80	11.12	11.87	10.59	12.00
1ig5	LYS16	11.06	10.54	10.51	10.52	11.12	11.49	11.01	10.73	11.45	10.83	11.35
1ig5	LYS25	11.81	10.54	10.51	11.40	10.86	10.52	11.25	10.84	11.78	12.12	12.00
1ig5	LYS29	11.28	10.54	10.51	10.33	10.72	11.03	10.79	10.62	11.12	10.40	10.82
1ig5	LYS41	10.93	10.54	10.51	10.39	10.73	10.60	10.46	10.45	10.85	10.07	10.48
1ig5	LYS55	12.12	10.54	10.51	10.80	11.25	11.58	11.76	11.11	2.99	11.72	12.00

1ig5	LYS71	10.73	10.54	10.51	10.19	10.53	10.73	10.34	10.39	10.98	9.89	10.31
1ig5	ASP47	3.04	3.90	3.68	2.64	3.12	2.69	2.70	3.27	2.92	2.75	2.61
1kf3, 9rat, 3rn3, 1rnz, 3srn	HIS105	6.50	6.04	6.57	6.89	5.13	6.58	6.86	6.83	6.85	8.16	6.75
1kf3, 9rat, 3rn3, 1rnz, 3srn	GLU2	2.70	4.07	4.28	2.75	3.49	3.28	1.99	3.05	2.57	1.81	2.16
1kf3, 9rat, 3rn3, 1rnz, 3srn	GLU86	4.00	4.07	4.28	4.10	4.01	3.46	4.28	4.19	4.18	4.60	3.49
1kf3, 9rat, 3rn3, 1rnz, 3srn	ASP38	2.10	3.90	3.68	3.68	3.04	2.81	3.31	3.55	3.45	3.37	2.86
1kf3, 9rat, 3rn3, 1rnz, 3srn	ASP121	3.00	3.90	3.68	2.83	3.69	2.51	1.49	2.67	3.14	1.36	1.74
1lni, 1rgg	HIS53	8.27	6.04	6.57	5.91	7.34	8.85	7.26	7.03	7.18	9.03	7.81
1lni, 1rgg	GLU78	3.13	4.07	4.28	3.46	3.74	4.06	4.13	4.13	3.97	4.68	3.66
1lni, 1rgg	ASP79	7.37	3.90	3.68	5.67	4.77	5.36	4.55	4.15	4.11	6.39	5.40
1lni, 1rgg	ASP93	3.09	3.90	3.68	2.73	3.40	3.48	3.54	3.63	3.99	3.89	3.78
1lse, 6lyz, 4lzt, 2lzt, 1lys	GLU7	2.85	4.07	4.28	3.38	4.06	3.55	2.98	3.61	3.21	3.27	2.96
1lse, 6lyz, 4lzt, 2lzt, 1lys	GLU35	6.20	4.07	4.28	6.65	4.75	5.06	4.25	4.27	3.63	5.01	4.08
1lse, 6lyz, 4lzt, 2lzt, 1lys	LYS116	10.40	10.54	10.51	10.19	10.57	10.42	10.10	10.27	10.83	9.67	10.20
1lse, 6lyz, 4lzt, 2lzt, 1lys	ASP18	2.66	3.90	3.68	3.52	2.85	2.03	2.97	3.35	3.02	2.97	2.75
1lse, 6lyz, 4lzt, 2lzt, 1lys	ASP48	1.60	3.90	3.68	2.49	2.91	1.91	2.61	3.20	3.39	2.92	2.85
1lse, 6lyz, 4lzt, 2lzt, 1lys	ASP87	2.07	3.90	3.68	2.82	3.03	2.02	2.80	3.28	2.89	2.67	2.28
1mut	LYS39	8.40	10.54	10.51	10.40	10.82	10.75	10.67	10.55	11.12	10.21	10.73
1pga, 3gb1, 2gb1, 1pgb, 1gb1	GLU56	4.00	4.07	4.28	5.10	3.85	4.29	4.31	4.24	3.73	4.50	3.33
1sap	GLU47	4.21	4.07	4.28	3.82	3.56	3.58	3.80	4.00	3.17	3.96	3.77
1sap	GLU53	3.53	4.07	4.28	5.53	4.16	3.74	4.10	4.18	3.81	5.49	4.62
1sap	ASP16	2.89	3.90	3.68	2.34	2.70	2.14	3.05	3.40	3.09	2.44	2.22
1sap	ASP36	3.12	3.90	3.68	2.33	3.13	2.97	2.37	3.07	2.75	1.50	1.62
1trs, 1trw	GLU13	4.40	4.07	4.28	4.61	4.23	4.58	4.13	4.15	3.83	4.80	4.22
1trs, 1trw	GLU56	3.20	4.07	4.28	4.51	4.01	3.56	3.25	3.77	2.96	3.11	2.75
1trs, 1trw	ASP60	3.90	3.90	3.68	4.49	3.92	2.25	3.58	3.63	3.87	5.08	4.34
1trs, 1trw	ASP61	5.20	3.90	3.68	4.22	4.25	3.74	4.30	4.05	4.30	5.11	4.58
1ubq	GLU16	3.90	4.07	4.28	4.38	3.88	4.07	4.00	4.10	3.89	4.27	3.77
1ubq	GLU18	4.30	4.07	4.28	4.48	4.16	2.54	4.09	4.12	3.96	4.15	3.78
2bca	LYS12	11.06	10.54	10.51	10.55	10.66	11.29	11.12	10.74	11.16	9.92	10.78

2bca	LYS16	11.06	10.54	10.51	10.23	11.15	11.30	11.34	10.91	11.89	11.84	12.00
2bca	LYS29	11.28	10.54	10.51	10.12	10.27	11.17	10.32	10.40	10.98	9.74	10.39
2bca	LYS41	10.93	10.54	10.51	10.28	10.33	10.73	10.61	10.48	10.97	9.92	10.63
2bca	LYS55	12.12	10.54	10.51	10.55	11.20	11.51	11.05	10.75	11.42	11.27	11.40
2igh	ASP45	4.40	3.90	3.68	3.85	3.50	3.21	3.65	3.71	3.79	5.27	4.13
2ovo	GLU19	3.21	4.07	4.28	4.00	3.45	3.25	3.50	3.81	3.19	3.70	3.17
2ovo	GLU43	4.81	4.07	4.28	4.66	4.35	4.55	4.20	4.22	3.99	5.01	4.42
2ovo	ASP7	2.67	3.90	3.68	2.58	2.89	2.67	3.34	3.57	3.36	3.40	2.57
2ovo	ASP27	2.30	3.90	3.68	2.61	3.30	4.96	3.13	3.43	3.50	3.53	3.24
2qmt	GLU15	4.40	4.07	4.28	3.83	4.09	3.95	3.53	3.85	3.51	3.99	3.58
2qmt	ASP22	2.90	3.90	3.68	2.53	2.07	2.09	3.04	3.40	3.02	3.45	2.83
2qmt	ASP36	3.80	3.90	3.68	3.50	4.19	4.47	3.67	3.71	3.85	4.96	4.06
2qmt	ASP40	4.00	3.90	3.68	3.96	3.64	2.95	3.80	3.81	4.00	4.45	4.14
2zta	HIS18	6.24	6.04	6.57	6.24	6.20	6.37	6.71	6.75	6.29	7.44	6.60
2zta	GLU11	4.05	4.07	4.28	4.72	3.68	3.87	3.33	3.76	3.71	4.67	3.19
2zta	LYS8	11.26	10.54	10.51	10.51	10.77	10.67	11.14	10.79	11.35	10.72	11.44
2zta	LYS27	11.10	10.54	10.51	10.42	10.59	10.53	11.01	10.71	10.86	9.45	10.99
9rnt, 1ygw	HIS27	7.57	6.04	6.57	5.61	7.44	6.79	6.90	6.78	6.91	8.18	7.12
9rnt, 1ygw	HIS40	7.44	6.04	6.57	6.44	6.83	7.49	6.89	6.77	7.02	10.11	7.37
9rnt, 1ygw	GLU28	5.61	4.07	4.28	4.99	4.13	5.12	4.41	4.29	3.96	4.88	4.78
9rnt, 1ygw	GLU31	5.36	4.07	4.28	4.66	4.53	4.36	4.52	4.36	4.16	5.12	4.59
9rnt, 1ygw	GLU46	3.62	4.07	4.28	4.67	4.22	4.86	4.16	4.17	3.85	5.94	4.21
9rnt, 1ygw	GLU102	5.30	4.07	4.28	5.60	4.89	4.37	5.00	4.61	4.16	6.23	5.26
9rnt, 1ygw	ASP15	3.52	3.90	3.68	4.20	2.95	2.79	4.18	3.94	3.88	4.61	3.93
9rnt, 1ygw	ASP49	4.22	3.90	3.68	3.69	3.82	3.99	4.26	4.00	4.15	4.63	4.28

Table S3. Experimental and predicted pK_a values from the Mutant Set (n=90).

PDB ID _ Applied SCWRL Mutation	Residue	Exp pKa	Soln pKa	Avg pKa	Propka3	DeepKa	PKAI	PKAI+	DelPhiPKa	MCCE2	H++
1DE3_E96Q	HIS35	6.30	6.04	6.57	5.94	6.72	6.98	6.90	6.55	7.61	6.80
1DE3_E96Q	HIS36	7.30	6.04	6.57	6.50	7.10	7.07	6.90	6.91	7.92	6.89
1DE3_E96Q	HIS50	6.40	6.04	6.57	4.04	6.63	6.06	6.30	6.13	4.02	6.21
1DE3_E96Q	HIS92	7.20	6.04	6.57	5.69	5.64	6.11	6.38	5.95	6.51	6.27
1DE3_E96Q	HIS137	5.90	6.04	6.57	4.70	5.35	4.30	5.68	5.30	3.40	6.32
1DE3_E96Q	HIS150	7.90	6.04	6.57	6.53	6.12	6.65	6.69	6.05	8.50	6.59
1DE3_H50Q	HIS35	6.20	6.04	6.57	5.94	6.72	6.98	6.90	6.63	7.64	6.92
1DE3_H50Q	HIS82	7.40	6.04	6.57	6.82	7.44	8.20	7.36	7.84	10.20	7.93
1DE3_H50Q	HIS92	7.30	6.04	6.57	5.69	5.64	6.11	6.38	6.02	6.61	6.40
1DE3_H50Q	GLU96	5.00	4.07	4.28	7.73	6.05	2.50	3.44	2.18	2.20	3.21
1DE3_H50Q	HIS137	6.10	6.04	6.57	4.73	5.33	5.25	5.94	6.03	5.45	6.90
1DE3_H50Q	HIS150	7.30	6.04	6.57	6.53	6.12	6.65	6.69	6.14	8.49	6.61
1DE3_H137Q	HIS35	6.30	6.04	6.57	5.94	6.72	6.98	6.90	6.63	7.61	6.90
1DE3_H137Q	HIS82	7.40	6.04	6.57	6.81	7.47	8.27	7.40	7.84	10.20	8.00
1DE3_H137Q	HIS92	7.10	6.04	6.57	5.69	5.64	6.11	6.38	6.01	6.55	6.25
1DE3_H137Q	GLU96	5.70	4.07	4.28	7.82	5.57	2.05	3.17	2.08	1.13	2.98
1DE3_H137Q	HIS104	6.60	6.04	6.57	6.08	6.57	6.48	6.57	6.58	6.19	6.62
1DE3_H137Q	HIS150	7.70	6.04	6.57	6.53	6.12	6.65	6.69	6.14	8.54	6.63
1DE3_H50Q_H137Q	HIS35	6.30	6.04	6.57	5.94	6.72	6.98	6.90	6.64	7.65	6.91
1DE3_H50Q_H137Q	HIS36	7.30	6.04	6.57	6.50	7.10	7.07	6.90	6.94	8.04	6.96
1DE3_H50Q_H137Q	HIS82	7.00	6.04	6.57	6.82	7.44	8.20	7.36	7.83	10.18	7.93
1DE3_H50Q_H137Q	HIS92	7.40	6.04	6.57	5.69	5.64	6.11	6.38	6.04	6.60	6.39
1DE3_H50Q_H137Q	GLU96	5.40	4.07	4.28	7.72	6.19	3.10	3.68	2.64	3.64	3.64
1DE3_H50Q_H137Q	HIS104	7.30	6.04	6.57	6.08	6.57	6.48	6.57	6.61	6.20	6.72
1DE3_H50Q_H137Q	HIS150	7.60	6.04	6.57	6.53	6.12	6.65	6.69	6.16	8.54	6.62
1STN_K9A	HIS8	6.73	6.04	6.57	6.37	6.49	6.78	6.75	6.45	7.86	6.75

1STN_K9A	HIS46	6.47	6.04	6.57	6.28	3.50	2.63	4.68	5.75	0.00	5.84
1STN_K9A	HIS121	5.48	6.04	6.57	6.55	6.15	6.88	6.79	6.63	7.23	6.83
1STN_K9A	HIS124	5.67	6.04	6.57	6.03	5.98	6.20	6.48	5.72	6.63	6.16
1STN_E73A	HIS8	6.47	6.04	6.57	6.36	6.34	6.57	6.65	6.19	7.59	6.38
1STN_E73A	HIS46	6.51	6.04	6.57	6.28	3.50	2.63	4.68	5.73	0.00	5.83
1STN_E73A	HIS121	5.24	6.04	6.57	6.42	5.96	6.55	6.66	6.13	6.70	6.41
1STN_E73A	HIS124	5.63	6.04	6.57	6.02	5.92	6.19	6.47	5.52	6.43	6.02
1STN_D77A	HIS8	6.44	6.04	6.57	6.36	6.27	6.66	6.68	6.20	7.57	6.41
1STN_D77A	HIS46	6.43	6.04	6.57	6.28	3.50	2.63	4.68	5.73	0.00	5.82
1STN_D77A	HIS121	5.89	6.04	6.57	6.25	5.47	6.21	6.50	5.96	5.84	6.05
1STN_D77A	HIS124	5.77	6.04	6.57	6.03	5.92	6.10	6.42	5.52	6.49	6.01
1STN_D77N	HIS8	6.29	6.04	6.57	6.36	6.33	6.65	6.67	6.21	7.58	6.42
1STN_D77N	HIS46	6.53	6.04	6.57	6.28	3.50	2.63	4.68	5.72	0.00	5.83
1STN_D77N	HIS121	5.77	6.04	6.57	6.22	5.63	6.19	6.42	5.94	5.53	6.04
1STN_D77N	HIS124	5.81	6.04	6.57	6.01	5.92	6.16	6.46	5.52	6.54	6.02
1STN_Y91A	HIS8	6.42	6.04	6.57	6.36	6.37	6.69	6.69	6.29	7.67	6.51
1STN_Y91A	HIS46	6.52	6.04	6.57	6.28	3.50	2.63	4.68	5.74	0.00	5.82
1STN_Y91A	HIS121	5.32	6.04	6.57	6.67	5.82	6.57	6.64	6.28	7.17	6.46
1STN_Y91A	HIS124	5.82	6.04	6.57	6.05	5.78	6.14	6.45	5.58	6.47	6.08
1STN_Y91F	HIS8	6.36	6.04	6.57	6.36	6.37	6.69	6.69	6.29	7.75	6.52
1STN_Y91F	HIS46	6.39	6.04	6.57	6.28	3.50	2.63	4.68	5.74	0.00	5.83
1STN_Y91F	HIS121	6.04	6.04	6.57	6.42	6.19	6.57	6.64	6.32	6.52	6.50
1STN_Y91F	HIS124	5.65	6.04	6.57	6.02	5.95	6.14	6.45	5.57	6.50	6.07
1STN_Y93A	HIS8	6.45	6.04	6.57	6.36	6.34	6.70	6.70	6.28	7.73	6.52
1STN_Y93A	HIS46	6.48	6.04	6.57	6.28	3.50	2.63	4.68	5.74	0.00	5.82
1STN_Y93A	HIS121	5.94	6.04	6.57	6.51	5.86	6.65	6.73	6.19	6.78	6.54
1STN_Y93A	HIS124	5.68	6.04	6.57	6.05	5.83	6.14	6.44	5.65	6.47	6.07
1STN_Y93F	HIS8	6.46	6.04	6.57	6.36	6.35	6.70	6.70	6.28	7.74	6.52
1STN_Y93F	HIS46	6.42	6.04	6.57	6.28	3.50	2.63	4.68	5.74	0.00	5.83
1STN_Y93F	HIS121	4.00	6.04	6.57	6.43	5.86	6.65	6.73	6.17	6.85	6.53

1STN_Y93F	HIS124	5.52	6.04	6.57	6.02	5.83	6.14	6.44	5.59	6.45	6.06
1STN_E101A	HIS8	6.49	6.04	6.57	6.36	6.37	6.68	6.68	6.25	7.71	6.52
1STN_E101A	HIS46	6.40	6.04	6.57	6.28	3.50	2.63	4.68	5.72	0.00	5.82
1STN_E101A	HIS121	5.26	6.04	6.57	6.42	5.89	6.50	6.63	6.13	6.71	6.39
1STN_E101A	HIS124	5.04	6.04	6.57	5.84	5.79	5.76	6.24	5.20	5.93	5.70
1STN_K127A	HIS8	6.51	6.04	6.57	6.36	6.37	6.68	6.68	6.30	7.74	6.52
1STN_K127A	HIS46	6.45	6.04	6.57	6.28	3.50	2.63	4.68	5.75	0.00	5.83
1STN_K127A	HIS121	5.30	6.04	6.57	6.42	5.95	6.69	6.70	6.41	6.93	6.60
1STN_K127A	HIS124	5.99	6.04	6.57	6.11	6.08	6.36	6.54	5.87	6.86	6.36
1STN_S128A	HIS8	6.53	6.04	6.57	6.36	6.37	6.68	6.68	6.29	7.72	6.52
1STN_S128A	HIS46	6.49	6.04	6.57	6.28	3.50	2.63	4.68	5.74	0.00	5.83
1STN_S128A	HIS121	5.25	6.04	6.57	6.41	5.95	6.52	6.63	6.28	6.80	6.51
1STN_S128A	HIS124	5.71	6.04	6.57	6.03	5.89	6.08	6.39	5.57	6.52	5.98
2LKV_I92E	GLU92	9.00	4.07	4.28	8.23	8.02	5.69	4.99	2.54	8.14	4.30
2LKV_L103E	GLU103	8.90	4.07	4.28	10.05	7.40	5.80	5.01	3.54	9.89	4.83
2LKV_L36E	GLU36	8.70	4.07	4.28	9.46	7.57	6.67	5.37	2.71	9.06	4.41
2LKV_V66E	GLU66	8.50	4.07	4.28	9.14	6.50	6.49	5.34	2.59	7.11	3.76
2LKV_V99E	GLU99	8.40	4.07	4.28	9.73	8.08	5.97	5.13	3.57	6.23	4.28
2LKV_V39E	GLU39	8.20	4.07	4.28	11.65	3.81	7.32	5.65	2.72	8.63	4.78
2LKV_V74E	GLU74	7.80	4.07	4.28	11.15	7.00	6.94	5.72	2.37	7.65	4.46
2LKV_T62E	GLU62	7.70	4.07	4.28	10.87	6.95	6.33	5.38	2.95	9.32	4.53
2LKV_N100E	GLU100	7.60	4.07	4.28	11.99	5.08	2.41	3.06	3.95	7.90	2.82
2LKV_L25E	GLU25	7.50	4.07	4.28	8.80	7.06	6.52	5.36	2.64	8.11	4.13
2LKV_F34E	GLU34	7.30	4.07	4.28	9.23	8.03	6.46	5.34	6.98	7.43	4.08
2LKV_I72E	GLU72	7.30	4.07	4.28	7.03	4.86	6.73	5.38	3.46	6.53	4.42
2LKV_V23E	GLU23	7.10	4.07	4.28	10.80	3.97	5.96	5.16	2.50	7.42	3.73
2LKV_Y91E	GLU91	7.10	4.07	4.28	8.97	3.46	5.29	4.43	2.29	7.65	3.29
2LKV_A132E	GLU132	7.00	4.07	4.28	10.74	7.09	3.62	3.34	3.85	14.00	4.98
2LKV_L38E	GLU38	6.80	4.07	4.28	8.50	4.07	5.00	4.61	1.72	6.35	2.81
2LKV_T41E	GLU41	6.80	4.07	4.28	9.23	4.85	7.09	5.56	3.58	7.08	5.17

2LKV_A90E	GLU90	6.40	4.07	4.28	11.52	7.86	6.32	5.13	5.47	8.35	4.18
2LKV_L37E	GLU37	5.20	4.07	4.28	10.83	6.49	2.85	4.56	1.41	10.52	2.43
2LKV_G20E	GLU20	4.50	4.07	4.28	10.87	2.70	6.25	5.21	2.87	6.87	4.43
2LKV_N118E	GLU118	4.50	4.07	4.28	8.61	4.04	5.17	4.63	4.66	3.15	3.69

Table S4. Grouping of PDB entries corresponding to proteins with identical amino-acid sequences. The pK_a predictions were averaged within each group.

Group	Target	Target File Name	Origin
**1**	1A2P_1~3	1A2P_curated_1~3.pdb	1A2P
	1A2P_4~23	1A2P_curated_4~23.pdb	1FW7
	1A2P_24~43	1A2P_curated_24~43.pdb	1BNR
	1A2P_44~46	1A2P_curated_44~46.pdb	1BNJ
	1A2P_47~49	1A2P_curated_47~49.pdb	1BNI
**2**	1BEO_1	1BEO_curated_1.pdb	1BEO
	1BEO_2~19	1BEO_curated_2~19.pdb	1BEG
**3**	1BHC_1	1BHC_curated_1.pdb	1BHC
	1BHC_2	1BHC_curated_2.pdb	4PTI
	1BHC_3~4	1BHC_curated_3~4.pdb	1D0D
	1BHC_5	1BHC_curated_5.pdb	1BPI
**4**	1BUS_1~5	1BUS_curated_1~5.pdb	1BUS
	2BUS_6	2BUS_curated_6.pdb	2BUS
**5**	1CVO_1~2	1CVO_curated_1~2.pdb	1CVO
	1CVO_3	1CVO_curated_3.pdb	1KXI
**6**	1EGF_1~16	1EGF_curated_1~16.pdb	1EGF
	1EGF_17~32	1EGF_curated_17~32.pdb	3EGF
	1EGF_33	1EGF_curated_33.pdb	1EPI
	1EGF_34~43	1EGF_curated_34~43.pdb	1EPH
	1EGF_44	1EGF_curated_44.pdb	1EPG
**7**	1ERA_1	1ERA_curated_1.pdb	1ERA
	1ERA_2	1ERA_curated_2.pdb	3EBX

**8**	1ERT_1	1ERT_curated_1.pdb	1ERT
	1ERT_2	1ERT_curated_2.pdb	1ERU

**9**	1EY0_1	1EY0_curated_1.pdb	1EY0
	1EY0_2	1EY0_curated_2.pdb	1STN

**10**	1KF3_1	1KF3_curated_1.pdb	1KF3
	1KF3_2	1KF3_curated_2.pdb	9RAT
	1KF3_3	1KF3_curated_3.pdb	3RN3
	1KF3_4	1KF3_curated_4.pdb	1RNZ
	1KF3_5	1KF3_curated_5.pdb	3SRN

**11**	1LNI_1~2	1LNI_curated_1~2.pdb	1LNI
	1LNI_3~4	1LNI_curated_3~4.pdb	1RGG

**12**	1LSE_1	1LSE_curated_1.pdb	1LSE
	1LSE_2	1LSE_curated_2.pdb	6LYZ
	1LSE_3	1LSE_curated_3.pdb	4LZT
	1LSE_4	1LSE_curated_4.pdb	2LZT
	1LSE_5~6	1LSE_curated_5~6.pdb	1LYS

**13**	1PGA_1	1PGA_curated_1.pdb	1PGA
	1PGA_2~33	1PGA_curated_2~33.pdb	3GB1
	1PGA_34	1PGA_curated_34.pdb	2GB1
	1PGA_35	1PGA_curated_35.pdb	1PGB
	1PGA_36~95	1PGA_curated_36~95.pdb	1GB1

**14**	1PNT_1	1PNT_curated_1.pdb	1PNT
	1PNT_2	1PNT_curated_2.pdb	1Z12

**15**	1TRS_1	1TRS_curated_1.pdb	1TRS
	1TRS_2	1TRS_curated_2.pdb	1TRW

**16**	9RNT_1	9RNT_curated_1.pdb	9RNT
	9RNT_2~35	9RNT_curated_2~35.pdb	1YGW

Table S5. Error metrics for pK_a predictors tested on various data sets. Bootstrapped median values (n = 10,000), as well as the upper and lower bounds of the 95% confidence interval, are listed.

MUE	Data set	Soln p*Ka*	Avg p*Ka*	Propka3	DeepKa	cpHMD	PKAI	PKAI+	DelPhiPKa	MCCE2	H++
Bootstrapped	large	0.68	0.64	0.64	0.60	N/A	0.72	0.62	0.79	0.74	0.68
Upper	large	0.75	0.71	0.70	0.65	N/A	0.80	0.69	0.88	0.81	0.75
Lower	large	0.61	0.57	0.58	0.55	N/A	0.64	0.55	0.70	0.67	0.61
Bootstrapped	small	1.02	0.97	0.85	0.78	0.74	0.96	0.92	1.03	0.96	0.88
Upper	small	1.18	1.13	0.99	0.90	0.86	1.15	1.08	1.26	1.12	1.05
Lower	small	0.86	0.81	0.71	0.66	0.62	0.77	0.76	0.80	0.80	0.71
Bootstrapped	asp	0.81	0.75	0.65	0.62	N/A	0.71	0.66	0.71	0.70	0.66
Upper	asp	0.95	0.89	0.76	0.72	N/A	0.83	0.79	0.86	0.79	0.78
Lower	asp	0.67	0.61	0.54	0.52	N/A	0.59	0.53	0.56	0.61	0.54
Bootstrapped	glu	0.53	0.50	0.58	0.59	N/A	0.74	0.55	0.82	0.71	0.70
Upper	glu	0.61	0.58	0.67	0.66	N/A	0.86	0.64	0.92	0.82	0.80
Lower	glu	0.45	0.42	0.49	0.52	N/A	0.62	0.46	0.72	0.60	0.60
Bootstrapped	his	0.98	0.81	0.90	0.66	N/A	0.80	0.73	0.75	1.17	0.70
Upper	his	1.20	1.02	1.13	0.83	N/A	1.01	0.90	0.96	1.51	0.88
Lower	his	0.76	0.60	0.67	0.49	N/A	0.59	0.56	0.54	0.83	0.52
Bootstrapped	lys	0.65	0.66	0.62	0.51	N/A	0.65	0.62	0.97	0.64	0.68
Upper	lys	0.82	0.83	0.73	0.64	N/A	0.83	0.78	1.35	0.77	0.88
Lower	lys	0.48	0.49	0.51	0.38	N/A	0.47	0.46	0.59	0.51	0.48
RMSE	Data set	Soln pKa	Avg pKa	Propka3	DeepKa	cpHMD	PKAI	PKAI+	DelPhiPKa	MCCE2	H++
Bootstrapped	large	1.02	0.98	0.93	0.81	N/A	1.09	0.94	1.24	1.08	1.01
Upper	large	1.15	1.12	1.04	0.89	N/A	1.23	1.07	1.44	1.25	1.14
Lower	large	0.89	0.84	0.82	0.73	N/A	0.95	0.81	1.04	0.91	0.88
Bootstrapped	small	1.37	1.33	1.16	1.00	1.00	1.43	1.29	1.64	1.35	1.30
Upper	small	1.61	1.59	1.38	1.14	1.16	1.76	1.56	2.09	1.68	1.58
Lower	small	1.13	1.07	0.94	0.86	0.84	1.10	1.02	1.19	1.02	1.02
Bootstrapped	asp	1.22	1.19	0.96	0.86	N/A	1.08	1.08	1.21	0.92	1.02
Upper	asp	1.47	1.47	1.15	1.00	N/A	1.33	1.36	1.56	1.05	1.26
Lower	asp	0.97	0.91	0.77	0.72	N/A	0.83	0.80	0.86	0.79	0.78

Bootstrapped	glu	0.77	0.74	0.86	0.78	N/A	1.12	0.83	1.08	1.09	1.00
Upper	glu	0.91	0.87	1.05	0.88	N/A	1.35	0.99	1.23	1.36	1.17
Lower	glu	0.63	0.61	0.67	0.68	N/A	0.89	0.67	0.93	0.82	0.83
Bootstrapped	his	1.23	1.08	1.20	0.89	N/A	1.08	0.94	1.06	1.66	0.94
Upper	his	1.49	1.37	1.51	1.12	N/A	1.38	1.15	1.39	2.29	1.20
Lower	his	0.97	0.79	0.89	0.66	N/A	0.78	0.73	0.73	1.03	0.68
Bootstrapped	lys	0.91	0.92	0.74	0.70	N/A	0.95	0.87	1.75	0.79	1.02
Upper	lys	1.22	1.22	0.87	0.91	N/A	1.26	1.15	2.60	0.95	1.37
Lower	lys	0.60	0.62	0.61	0.49	N/A	0.64	0.59	0.90	0.63	0.67
**R2**	**Data set**	**Soln pKa**	**Avg pKa**	**Propka3**	**DeepKa**	**cpHMD**	**PKAI**	**PKAI+**	**DelPhiPKa**	**MCCE2**	**H++**
Bootstrapped	large	0.00	0.08	0.29	0.40	N/A	0.14	0.16	0.03	0.30	0.21
Upper	large	0.00	0.13	0.41	0.51	N/A	0.21	0.23	0.06	0.42	0.29
Lower	large	0.00	0.03	0.17	0.29	N/A	0.07	0.09	0.00	0.18	0.13
Bootstrapped	small	0.00	0.05	0.34	0.49	0.50	0.16	0.16	0.03	0.30	0.26
Upper	small	0.00	0.10	0.52	0.68	0.69	0.28	0.27	0.06	0.50	0.38
Lower	small	0.00	0.00	0.16	0.30	0.31	0.04	0.05	0.00	0.10	0.14
Bootstrapped	asp	0.00	0.00	0.38	0.49	N/A	0.25	0.24	0.11	0.47	0.32
Upper	asp	0.00	0.00	0.61	0.73	N/A	0.38	0.36	0.19	0.66	0.43
Lower	asp	0.00	0.00	0.15	0.25	N/A	0.12	0.12	0.03	0.28	0.21
Bootstrapped	glu	0.00	0.00	0.25	0.17	N/A	0.05	0.05	0.04	0.11	0.12
Upper	glu	0.00	0.00	0.41	0.27	N/A	0.10	0.10	0.08	0.21	0.20
Lower	glu	0.00	0.00	0.09	0.07	N/A	0.00	0.00	0.00	0.01	0.04
Bootstrapped	his	0.00	0.00	0.05	0.43	N/A	0.25	0.27	0.08	0.24	0.26
Upper	his	0.00	0.00	0.10	0.76	N/A	0.43	0.47	0.16	0.40	0.43
Lower	his	0.00	0.00	0.00	0.10	N/A	0.07	0.07	0.00	0.08	0.09
Bootstrapped	lys	0.00	0.00	0.36	0.48	N/A	0.12	0.12	0.07	0.46	0.10
Upper	lys	0.00	0.00	0.71	0.88	N/A	0.24	0.24	0.14	0.83	0.20
Lower	lys	0.00	0.00	0.01	0.08	N/A	0.00	0.00	0.00	0.09	0.00
**MeUE**	**Data set**	**Soln pKa**	**Avg pKa**	**Propka3**	**DeepKa**	**cpHMD**	**PKAI**	**PKAI+**	**DelPhiPKa**	**MCCE2**	**H++**

Bootstrapped	large	0.44	0.42	0.44	0.45	N/A	0.48	0.40	0.51	0.56	0.44
Upper	large	0.51	0.48	0.50	0.50	N/A	0.55	0.45	0.59	0.63	0.49
Lower	large	0.37	0.36	0.38	0.40	N/A	0.41	0.35	0.43	0.49	0.39
Bootstrapped	small	0.78	0.73	0.63	0.62	0.56	0.64	0.64	0.64	0.74	0.53
Upper	small	1.03	0.91	0.72	0.78	0.68	0.80	0.77	0.90	0.88	0.63
Lower	small	0.53	0.55	0.54	0.46	0.44	0.48	0.51	0.38	0.60	0.43
Bootstrapped	asp	0.50	0.47	0.39	0.48	N/A	0.50	0.39	0.39	0.58	0.42
Upper	asp	0.64	0.56	0.47	0.58	N/A	0.62	0.47	0.46	0.69	0.48
Lower	asp	0.36	0.38	0.31	0.38	N/A	0.38	0.31	0.32	0.47	0.36
Bootstrapped	glu	0.35	0.32	0.40	0.44	N/A	0.49	0.37	0.67	0.49	0.49
Upper	glu	0.37	0.37	0.46	0.49	N/A	0.60	0.45	0.78	0.59	0.59
Lower	glu	0.33	0.27	0.34	0.39	N/A	0.38	0.29	0.56	0.39	0.39
Bootstrapped	his	0.78	0.68	0.61	0.61	N/A	0.53	0.59	0.47	0.86	0.47
Upper	his	1.08	0.92	0.79	0.95	N/A	0.70	0.80	0.59	1.11	0.64
Lower	his	0.48	0.44	0.43	0.27	N/A	0.36	0.38	0.35	0.61	0.30
Bootstrapped	lys	0.50	0.51	0.55	0.39	N/A	0.40	0.40	0.47	0.51	0.41
Upper	lys	0.61	0.63	0.68	0.51	N/A	0.49	0.48	0.67	0.62	0.53
Lower	lys	0.39	0.39	0.42	0.27	N/A	0.31	0.32	0.27	0.40	0.29
**MSE**	**Data set**	**Soln pKa**	**Avg pKa**	**Propka3**	**DeepKa**	**cpHMD**	**PKAI**	**PKAI+**	**DelPhiPKa**	**MCCE2**	**H++**
Bootstrapped	large	-0.07	0.00	0.06	-0.21	N/A	-0.34	-0.17	-0.29	0.06	-0.29
Upper	large	0.03	0.10	0.15	-0.13	N/A	-0.24	-0.08	-0.17	0.17	-0.20
Lower	large	-0.17	-0.10	-0.03	-0.29	N/A	-0.44	-0.26	-0.41	-0.05	-0.38
Bootstrapped	small	-0.33	-0.27	-0.18	-0.29	-0.31	-0.56	-0.42	-0.54	-0.15	-0.48
Upper	small	-0.07	-0.02	0.03	-0.11	-0.13	-0.30	-0.18	-0.23	0.11	-0.25
Lower	small	-0.59	-0.52	-0.39	-0.47	-0.49	-0.82	-0.66	-0.85	-0.41	-0.71
Bootstrapped	asp	0.26	0.04	0.09	-0.16	N/A	-0.29	-0.08	-0.10	0.12	-0.29
Upper	asp	0.46	0.24	0.25	-0.02	N/A	-0.12	0.10	0.10	0.27	-0.13
Lower	asp	0.06	-0.16	-0.07	-0.30	N/A	-0.46	-0.26	-0.30	-0.03	-0.45
Bootstrapped	glu	-0.22	-0.01	0.20	-0.31	N/A	-0.58	-0.34	-0.74	0.03	-0.52
Upper	glu	-0.11	0.10	0.33	-0.21	N/A	-0.43	-0.22	-0.62	0.20	-0.39
Lower	glu	-0.33	-0.12	0.07	-0.41	N/A	-0.73	-0.46	-0.86	-0.14	-0.65

Bootstrapped	his	-0.58	-0.06	-0.49	-0.27	N/A	-0.22	-0.06	0.06	0.42	-0.03
Upper	his	-0.26	0.26	-0.16	0.00	N/A	0.12	0.23	0.37	0.96	0.26
Lower	his	-0.90	-0.38	-0.82	-0.54	N/A	-0.56	-0.35	-0.25	-0.12	-0.32
Bootstrapped	lys	-0.02	-0.05	-0.14	0.06	N/A	0.29	0.08	0.52	-0.28	0.36
Upper	lys	0.22	0.19	0.06	0.25	N/A	0.53	0.31	1.05	-0.08	0.61
Lower	lys	-0.26	-0.29	-0.34	-0.13	N/A	0.05	-0.15	-0.01	-0.48	0.11
**MeSE**	**Data set**	**Soln pKa**	**Avg pKa**	**Propka3**	**DeepKa**	**cpHMD**	**PKAI**	**PKAI+**	**DelPhiPKa**	**MCCE2**	**H++**
Bootstrapped	large	-0.05	0.03	0.07	-0.24	N/A	-0.27	-0.14	-0.29	0.16	-0.20
Upper	large	0.03	0.09	0.16	-0.14	N/A	-0.22	-0.09	-0.21	0.25	-0.12
Lower	large	-0.13	-0.03	-0.02	-0.34	N/A	-0.32	-0.19	-0.37	0.07	-0.28
Bootstrapped	small	-0.25	-0.18	-0.21	-0.28	-0.25	-0.32	-0.27	-0.17	0.00	-0.26
Upper	small	-0.02	0.06	-0.01	-0.15	0.01	-0.05	-0.08	0.08	0.30	-0.08
Lower	small	-0.48	-0.42	-0.41	-0.41	-0.51	-0.59	-0.46	-0.42	-0.30	-0.44
Bootstrapped	asp	0.33	0.11	0.05	-0.16	N/A	-0.21	-0.05	-0.02	0.20	-0.10
Upper	asp	0.46	0.24	0.15	0.00	N/A	-0.09	0.05	0.13	0.34	0.06
Lower	asp	0.20	-0.02	-0.05	-0.32	N/A	-0.33	-0.15	-0.17	0.06	-0.26
Bootstrapped	glu	-0.18	0.03	0.22	-0.35	N/A	-0.39	-0.21	-0.65	0.17	-0.39
Upper	glu	-0.13	0.08	0.31	-0.29	N/A	-0.22	-0.11	-0.56	0.28	-0.27
Lower	glu	-0.23	-0.02	0.13	-0.41	N/A	-0.56	-0.31	-0.74	0.06	-0.51
Bootstrapped	his	-0.61	-0.08	-0.44	-0.19	N/A	-0.11	-0.01	-0.01	0.70	-0.08
Upper	his	-0.16	0.37	-0.24	0.28	N/A	0.12	0.39	0.40	0.92	0.12
Lower	his	-1.06	-0.53	-0.64	-0.66	N/A	-0.34	-0.41	-0.42	0.48	-0.28
Bootstrapped	lys	-0.20	-0.23	-0.32	-0.06	N/A	0.04	-0.19	0.39	-0.38	0.10
Upper	lys	-0.01	-0.04	-0.13	0.15	N/A	0.23	-0.05	0.54	-0.27	0.36
Lower	lys	-0.39	-0.42	-0.51	-0.27	N/A	-0.15	-0.33	0.24	-0.49	-0.16

Table S6. Summary of classification accuracies of various pK_a predictors applied to different amino acid types.

Residue type	Soln pKa	Avg pKa	PROPKA3	DeepKa	PKAI	PKAI+	DelPhiPKa	MCCE2	H++
**Asp**	51.8%	51.8%	58.1%	55.3%	58.8%	55.3%	60.3%	56.8%	61.7%
**Glu**	64.9%	64.9%	53.4%	54.5%	48.8%	59.7%	42.6%	50.6%	51.1%
**His**	39.0%	41.5%	41.5%	60.9%	51.2%	56.1%	46.4%	36.6%	46.4%
**Lys**	46.9%	46.9%	51.0%	57.1%	61.1%	51.0%	55.1%	55.0%	61.2%

Table S7. Outlier analysis performed for all tested methods. The predicted value for each method is shown if it was more than 2 pK_a units away from that of the experiment. Blank signifies that the predicted value was within 2 pK_a units from the experiment.

PDB_ID Residue	Expt p*Ka*	Soln p*Ka*	Avg p*Ka*	PROPKA3	DeepKa	PKAI	PKAI+	DelPhiPKa	MCCE2	H++
1h4g HIS11	6.52					4.14			0	
1h4g HIS60	4.01	6.04	6.57				6.31	6.79	6.12	6.64
1ey0 HIS46	5.86					2.3			0.63	
3ssi HIS43	3.25	6.04	6.57	6.04			5.35	6.75		6.76
1lni HIS53	8.27	6.04		5.91						
9rnt HIS40	7.44								10.11	
9rnt HIS92	7.31			5.08		5.19				
1pnt HIS66	8.29	6.04								
1pnt HIS72	9.19	6.04	6.57	5.79	5.97		7.05	7.06		7.13
1ptd HIS92	5.4							7.84		
1l54 LYS102	6.5	10.54	10.51		8.82	9.82	10.12	11.06		10.73
2ovo LYS13	9.86							11.93		
1nzp LYS312	9.58							11.66		11.62
1l54 LYS102	6.5	10.54	10.51		8.82	9.82	10.12	11.06		10.84
1beo LYS94	9.4							11.61		
1mut LYS39	8.4	10.54	10.51		10.82	10.67	10.55	11.12		10.79
1fez LYS53	9.3					12.3		13.7		13.54
1ig5 LYS55	12.12							2.99		
1l98 GLU105	6					2.64	3.55	3.22		
1a2p GLU73	2.1		4.28	5.24						
1h4g GLU17	4.31					2.27				
1h4g GLU178	4.1					0.24		1.8	0	1.82
1h4g GLU184	6.5	4.07	4.28			4.04	4.1	3.72		4.21
1de3 GLU96	5.4					1.48	2.88	2.6	0.41	2.44
1ert GLU6	4.8								7.71	
1hng GLU41	6.73	4.07	4.28	4.42	4.22	3.83	3.96	3.44		3.52
1lni GLU74	3.47								5.5	
2igh GLU32	4.6								7.73	
1hv1 GLU78	4.6			7.57						
1hv1 GLU172	7.2	4.07	4.28			4.12	4.07	3.76		4.24
9rnt GLU46	3.62								5.94	
9rnt GLU58	3.96			7.3		1.6				
1hv0 GLU78	4.6				6.97					
1hv0 GLU172	7.2	4.07	4.28			4.58	4.22	3.62		4.24
1lse GLU35	6.2	4.07						3.63		3.95
1ig5 GLU17	3.62			5.88						
1cdc GLU29	4.42			0.75		0.59	1.93	1.44	0	1.39
1cdc GLU41	6.73	4.07	4.28		3.94	0.96	2.6	2.63	0	3.18
135l GLU35	6.06							3.65		4
1a2p ASP54	2.2			4.4						
1a2p ASP75	3.1			5.58						
2lzm ASP70	0.5	3.9	3.68	3.99	3.73	2.67	3.19	2.99	3.17	2.85
2rn2 ASP10	6.1	3.9	3.68					3.89		
2rn2 ASP70	2.6			4.86						
1beo ASP30	2.51								4.57	
1a91 ASP44	5.6				3.47					
1a91 ASP61	7.1	3.9	3.68	4.14	4.63	4.12	3.96	3.99	4.88	4.14
1ert ASP26	9.9	3.9	3.68	7.43	7.62	4.5	4.06	3.71		3.59
1fna ASP67	4.2							10.52		
1lni ASP79	7.37	3.9	3.68		4.77	4.55	4.15	4.11		4.74
2trx ASP26	7.5	3.9	3.68			3.38	3.53	3.34		3.24
9rnt ASP76	0.5	3.9	3.68	5.07	3.49	4.22	3.9	3.55	4.1	3.46
1goa ASP10	6.1	3.9	3.68				4.07	3.45		3.88
1goa ASP70	2.6			4.7						
1lse ASP48	1.6	3.9	3.68							
1lse ASP66	0.9	3.9	3.68				2.96			
1lse ASP101	4.09								6.22	
1trs ASP26	8.1	3.9	3.68			3.91	3.82	3.68	6.09	3.32

References

(1) Sabini, E.; Sulzenbacher, G.; Dauter, M.; Dauter, Z.; Jørgensen, P. L.; Schülein, M.; Dupont, C.; Davies, G. J.; Wilson, K. S. *Chem. Biol.* **1999**, *6*, 483.

(2) Loftin, I. R.; Franke, S.; Blackburn, N. J.; McEvoy, M. M. *Protein Sci.* **2007**, *16*, 2287.

(3) Zhang, M.; Van Etten, R. L.; Stauffacher, C. V. *Biochemistry* **1994**, *33*, 11097.

(4) Abeygunawardana, C.; Weber, D. J.; Gittis, A. G.; Frick, D. N.; Lin, J.; Miller, A.-F.; Bessman, M. J.; Mildvan, A. S. *Biochemistry* **1995**, *34*, 14997.
